# Tetraploid Ancestry Provided Atlantic Salmon With Two Paralogue Functional T Cell Receptor Beta Regions Whereof One Is Completely Novel

**DOI:** 10.3389/fimmu.2022.930312

**Published:** 2022-06-17

**Authors:** Unni Grimholt, Arvind Y. M. Sundaram, Cathrine Arnason Bøe, Maria K. Dahle, Morten Lukacs

**Affiliations:** ^1^ Fish Health Research Section, Norwegian Veterinary Institute, Oslo, Norway; ^2^ Department of Medical Genetics, Oslo University Hospital, Oslo, Norway

**Keywords:** adaptive immunity, T cell receptor beta genes, Atlantic salmon, TRB, whole genome duplication

## Abstract

Protective cellular immune responses have been difficult to study in fish, due to lack of basic understanding of their T cell populations, and tools to study them. Cellular immunity is thus mostly ignored in vaccination and infection studies compared to humoral responses. High throughput sequencing, as well as access to well assembled genomes, now advances studies of cellular responses. Here we have used such resources to describe organization of T cell receptor beta genes in Atlantic salmon. Salmonids experienced a unique whole genome duplication approximately 94 million years ago, which provided these species with many functional duplicate genes, where some duplicates have evolved new functions or sub-functions of the original gene copy. This is also the case for T cell receptor beta, where Atlantic salmon has retained two paralogue T cell receptor beta regions on chromosomes 01 and 09. Compared to catfish and zebrafish, the genomic organization in both regions is unique, each chromosomal region organized with dual variable- diversity- joining- constant genes in a head to head orientation. Sequence identity of the chromosomal constant sequences between TRB01 and TRB09 is suggestive of rapid diversification, with only 67 percent as opposed to the average 82-90 percent for other duplicated genes. Using virus challenged samples we find both regions expressing bona fide functional T cell receptor beta molecules. Adding the 292 variable T cell receptor alpha genes to the 100 variable TRB genes from 14 subgroups, Atlantic salmon has one of the most diverse T cell receptor alpha beta repertoire of any vertebrate studied so far. Perhaps salmonid cellular immunity is more advanced than we have imagined.

## Introduction

Interactions between Major histocompatibility complex (MHC) molecules and T cell receptors (TR) provide vertebrates with the ability to discriminate between self and non-self. This ability enables the organisms to recognize invading pathogens, mount an immune response, and eliminate the infection. Our knowledge about teleost MHC and TR has gradually increased during the last 30 years, but there are still many gaps in our understanding of their role in cellular adaptive immunity. As many teleosts are essential aquacultural species, understanding adaptive immunity is important to provide efficient strategies to counteract infectious diseases and improving sustainability of this industry.

MHC molecules are among the most polymorphic genes known to date with more than 4000 different amino acid sequences currently registered for humans (www.ebi.ac.uk/ipd/imgt/hla/). The main variation between alleles resides in the residues that anchor peptides (1). Such a diversity enable these molecules to bind and present a large variety of self peptides and encountered non-self peptides. MHC class I (MHCI) molecules acquire self and non-self peptides from the intracellular space and present these peptides on the cell surface. In contrast, MHC class II molecules (MHCII) acquire peptides from the extracellular space, for instance from phagocytosed bacteria.

The vast variation in MHC and peptide sequences needs to be matched by variation in T cell receptor sequences to ensure detection of all pathogen invaders. MHCII molecules primarily interact with TR on CD4+ helper T cells while MHCI molecules interact with TR on CD8+ T cells (2). The conventional alpha beta T cell receptor is composed of one alpha (TRA) and one beta (TRB) chain. Both TRA and TRB chains consist of extracellular variable and constant domains in addition to transmembrane and cytoplasmic domains. TRA and TRB chains are encoded in large genomic regions where exons encoding a multitude of variable genes are located closely together followed by few diversity, several joining and a few constant genes. TRA, or more precisely TRA/TR delta (TRD), results from this region encoding both the TRA as well as the TRD molecule, where the delta chain is part of the gamma delta T cell receptor molecule expressed on unconventional T cells (3). In humans, the TRB and gamma (TRG) regions are located 104 mega bases (Mb) apart on chromosome (chr.) 7, while the TRA/TRD region is located on chr.14 (4, 5).

To accomplish a functional T cell receptor, each T cell undergoes genomic rearrangement of both the alpha as well as the beta chain loci (5, 6). One variable gene (V) is combined with one joining (J) gene and spliced to one constant (C) gene resulting in one expressed T cell receptor alpha chain. The TR beta chain has an additional diversity (D) gene in between the variable and joining genes, further increasing the TRB variation. Once T cell receptor loci have been rearranged, and a T cell receptor can be expressed at the membrane, the T cell is educated in the thymus to eliminate any T cells that respond too strongly or too poorly to MHC/self-peptide molecules, thus avoiding autoimmunity.

Each TRA/TRB chain has three complementary-determining regions (CDRs), which describe the regions that interact with the MHC-peptide complex (7, 8). The CDR1 and CDR2 loops reside within the variable alpha and beta domains and primarily interact with the MHC molecule while CDR3 primarily binds to the peptide (9, 10). For TRA, the CDR3 loop resides in between the variable and joining genes while the diversity element of TRB adds to the CDR3 variation. Non- template nucleotide additions and deletions of single or several nucleotides at these CDR3 junctional boundaries provide an almost infinite diversity in the CDR3 loop (11). Combinations of different alpha and beta chains add another layer of diversity. In humans, each individual can produce 10^6^ or more different T cell alpha and beta receptors, which can respond to a myriad of different MHC-peptide combinations (12, 13).

T cells and T-cell responses have been studied in many teleosts including rainbow trout and Atlantic salmon. The first salmonid TRB molecules were described more than 25 years ago (14–16). Ten TRB variable (TRBV) gene subgroups were further described in trout (17). Further studies into tissue distribution, T cell subsets and T-cell responses followed [reviewed in (18)]. As in mammals, teleost T cells are educated in the thymus (19). Teleosts differ from mammals by lack of lymph nodes, but have other unique tissue structures with abundant T cells, such as interbranchial lymphoid tissue (ILT) located in the gills close to thymus (20). T cells are also abundant in other mucosal tissues such as the digestive tract and skin (21–23). Similar to what is known from mammals, cytotoxicity has been demonstrated for some teleost CD8+ T cells while T- helper cell functions have been shown for some teleost CD4+ T cells (24–27). Memory T cells are still not described in teleosts, but the outline of a memory response is shown by long-term protection provided by vaccination as well as protection being passed through adoptive transfer of leukocytes from vaccinated donor fish to naive histocompatible recipients (28).

Linking clonally expanded TR cells to MHC-peptide is the ultimate goal for any vaccination or challenge study, enabling an understanding of TR immunity thus opening for improved vaccine design. High throughput sequencing and available analysis tools have enabled detection of TR repertoire changes, also for non-model species, where germline data are not available (29). For some species, curated sequence databases with germline as well as expressed V-D-J-C gene data are available, providing the scientific community with standardized tools for TR repertoire studies and comparison of data between different studies (30).

Assembly of genomic TR regions has been problematic due to many variable and joining genes with high sequence identity, and this has hampered comparative studies of these regions. The first TR region to be published in teleosts was the alpha delta TR region in Tetraodon (31). Tetraodon TRA/TRD had limited diversity with only 13 variable alpha/delta genes, 12 joining alpha genes and one constant alpha gene. The second region to be published was the 900 kilo base (kb) Atlantic salmon TRA/TRD region, which was published in 2008 and relied on sequencing of eight overlapping bacterial artificial clones (32). They discovered an unprecedented 292 variable TRA/TRD genes and 123 joining genes, exemplifying why these regions are very difficult to assemble. This region was recently revisited, expanding the understanding of Atlantic salmon TRA/TRD genes (33). Partial TRB regions containing D-J-C genes from catfish and rainbow trout were published in 1997 and 2003 (14, 34). The first complete TRB region to be published was the 270 kb zebrafish T cell receptor beta region using the quite extensively sequenced zebrafish genome (35). This region had only 52 variable genes and 28 joining genes. Since then, T cell receptor alpha or beta regions have only been published from catfish, which showed an intermediate complexity (36). The 214 kb catfish TRB region encoded 112 TRB variable genes and 31 TRBJ genes while the much larger TRA/TRD region of 1285 kb encoded 140 TRA/TRD variable genes and 125 TRA joining genes.

Recently, a new Atlantic salmon genome showed an assembly that enabled characterization of the T cell receptor beta complex. Adding expressed amplicon data we here show how the unique salmonid fourth whole genome duplication (4WGD) event has affected the T cell receptor beta repertoire in Atlantic salmon.

## Materials and Methods

### Genome Mining

We used published sequences for rainbow trout and Atlantic salmon T cell receptor beta variable, diversity, joining and constant genes (14–17) in blastN and tblastN search against the Atlantic salmon NCBI genome (https://www.ncbi.nlm.nih.gov/genome/) GCA_905237065.2 to identify Atlantic salmon TRB sequences. Chromosomal regions with match to several of the ten rainbow trout variable subgroup sequences, single diversity gene sequence, nine joining gene sequences as well as the constant domain sequence from both rainbow trout and Atlantic salmon (14, 15, 17) were subjected to closer scrutiny, where we extracted all matching sequences and performed manual inspection as well as phylogenies to verify TRB origin. Extracted sequences were used to design primers aimed at amplifying all members of each variable subgroup for the TRB09 region. Later a reverse primer was designed in the constant domain sequence to specifically amplify TRB sequences from the TRB01 regions using the forward primes designed for the TRB09 region variable sequences.

### Animals Used for Expression Analysis

*Piscine* orthoreovirus 1 (PRV-1) infected Atlantic salmon used for expression analyses originate from a trial performed at the Aquaculture Research station at Kårvika, Troms, Norway in 2019, approved by the Norwegian Animal Research Authority, and performed in accordance with the recommendations of the current animal welfare regulations: FOR-1996-01-15-23 (Norway). The full trial is previously published and described (37). Specifically, animals were infected through cohabitation with PRV-1 injected shedder fish of the same size in a 1:1 relationship in the tank. The shedder fish had two weeks earlier been injected intraperitonially with 0.2 mL of a newly prepared batch of PRV-1 blood cell lysate.

### Preparation of Samples

Spleen samples from eight infected salmon were taken eight weeks after exposure to PRV-1 and stored in 0.5 mL of RNALater (Qiagen, Hilden, Germany) (37). Tissue samples from the spleen (25 mg) on RNALater (Qiagen) were transferred to 0.65 mL Qiazol lysis reagent (Qiagen) containing a 5 mm steel bead and homogenized in a TissueLyzer II (Qiagen) for 2 × 5 min at 25 hertz. Followed by chloroform addition, mixing and centrifugation, and the aqueous phase was collected. RNeasy Mini QIAcube Kit (Qiagen) was used according to manufacturer guidelines for automated RNA isolation. RNA concentrations were quantified using the Nanodrop ND-100 spectrophotometer (Thermo Fisher Scientific, Waltham, MA, USA). RNA was eluted in RNase-free water and stored at −80°C until further use. 400 ng total spleen RNA per sample was reverse transcribed to cDNA using the QuantiTect Reverse Transcription Kit with gDNA wipeout buffer (Qiagen).

### Library Preparation and Sequencing

cDNA from eight randomly selected PRV-1 infected animals was mixed in equal quantitites. We used 50 ng of this cDNA mix in 20 µl PCR reactions with KAPA HiFi HotStart ReadyMix (Roche, Mannheim, Germany), with each of the TRB variable primers against one reverse primer for the TRB09 region (**Table 1**). For the TRB01 region we used only selected forward primers and the unique reverse primer. The following program was used for amplification: 95°C for 2 min; 30 cycles of 98°C for 20 s, 60°C for 15 s, 72°C for 60 s; 72°C for 1 min. Products were verified on a 1% agarose gel, cleaned using 1.5 × PCR volume of Agencourt AMPure XP PCR purification kit (Beckman Coulter, Brea, CA, USA) according to manufacturer’s recommendation and dissolved in 30 µl TE.

**Table 1:** Primer sequences used in this study.

First Forward primers	
V1.F	ACACTCTTTCCCTACACGACGCTCTTCCGATCTGGTCTTGTTGAGGGGAGTGA
V2.F	ACACTCTTTCCCTACACGACGCTCTTCCGATCTCCAGGGAGAGTCGGCTAAGA
V3.F	ACACTCTTTCCCTACACGACGCTCTTCCGATCTAGTTCAACTSAMCTGCAGMC
V4.F	ACACTCTTTCCCTACACGACGCTCTTCCGATCTGCCTAATGCCTCTGTCACACT
V5.F	ACACTCTTTCCCTACACGACGCTCTTCCGATCTAGGTTCACCAGACTCCCTCT
V7.F	ACACTCTTTCCCTACACGACGCTCTTCCGATCTGTCTCCTGTCCTGTCTGTGTG
V8.F	ACACTCTTTCCCTACACGACGCTCTTCCGATCTGTTGCGTTTCGTCAGTCTCC
V9.F	ACACTCTTTCCCTACACGACGCTCTTCCGATCTGTTCCTGTGTGTCCCTCTCC
V10.F1	ACACTCTTTCCCTACACGACGCTCTTCCGATCTTGGGCAGTAAAGTCCTCCAG
V10.F2	ACACTCTTTCCCTACACGACGCTCTTCCGATCTTCCAGGTTCCCTCAGCACTA
V11/13.F	ACACTCTTTCCCTACACGACGCTCTTCCGATCTTCAACTCACCTGCAGCCATACT
V12.F	ACACTCTTTCCCTACACGACGCTCTTCCGATCTATCTCCGCTGTTCTCACACT
**First Reverse primers**	
TRB01.R	GTGACTGGAGTTCAGACGTGTGCTCTTCCGATCTGTTGTCGGTCCCAACTCCAT
TRB09.R	GTGACTGGAGTTCAGACGTGTGCTCTTCCGATCTGACTTGCCAGAAGACCGTGA
**Second forward and reverse primers with index**	
PCR2-F	AATGATACGGCGACCACCGAGATCTACACGGACGGACACTCTTTCCCTACACGAC
PCR2-R	CAAGCAGAAGACGGCATACGAGATCGTGATGTGACTGGAGTTCAGACGTG

All PCR products were mixed in one library and subjected to 10 cycles of PCR using the second set of primers with KAPA HiFi HotStart ReadyMix (**Table 1**, PCR2 primers). The following program was used for amplification: 95°C for 2 min; ten cycles of 98°C for 20 s, 58°C for 30 s, 72°C for 60 s; 72°C for 10 min. Products were purified using 1.0 × PCR volume of Agencourt AMPure XP PCR purification kit (Beckman Coulter) according to manufacturer’s recommendation and dissolved in 30 µl TE.

The fragment size in the final pool was determined using the High Sensitivity D5000 kit for TapeStation (Agilent, Santa Clara, CA, USA) and dsDNA quantified using the High Sensitivity assay on a Qubit™ fluorometer (Invitrogen, Carlsbad, CA, USA). The library was diluted to 0.4 µg DNA in 130 µl TE-1 buffer as recommended for Illumina Nano sequencing. Due to low diversity in the 3’ parts of the amplicon, Illumina sequencing was performed with 20-30% Illumina spike-in PhiX control and loading reduced to 60% of recommended final loading concentration.

Sequencing was performed on a MiSeq (Illumina, San Diego, CA, USA) using 500 cycle v2 Nano kits generating 250 base pairs (bp) paired end reads. Sequence data have been submitted to NCBI SRA under the BioProject accession number PRJNA814480.

### Bioinformatics

Analysis of constant gene sequences was conducted using CLC Workbench (Qiagen).

Sequence data from Illumina were analysed as described in (38). Briefly, raw sequence data was cleaned to remove/trim low quality data, adapter sequences and PhiX (Illumina spike-in) using BBDuk v34.56 (part of BBTools; https://jgi.doe.gov/data-and-tools/bbtools/bb-tools-user-guide/). Based on the primers used during the first PCR reaction, cleaned reads were demultiplexed using demultiplexer v1.9 (https://github.com/nsc-norway/triple_index-demultiplexing). Paired end reads were merged together using FLASH v1.2.11 (39) with default settings and the unique fragments were counted using fastx_collapser (part of FASTX Toolkit v0.0.13; http://hannonlab.cshl.edu/fastx_toolkit/). Transeq was used for identifying open reading frames (40).

### Phylogenies

The evolutionary histories were inferred using the Neighbor-Joining method (41). The percentage of replicate trees in which the associated taxa clustered together in the bootstrap test (1000 replicates) are shown next to the branches (42). The trees are drawn to scale, with branch lengths in the same units as those of the evolutionary distances used to infer the phylogenetic tree. All ambiguous positions were removed for each sequence pair. Evolutionary analyses were conducted in MEGA7 (43).

## Results and Discussion

Using available salmonid V-D-J-C sequences (14–17), we investigated the presence of similar sequences in the Atlantic salmon genome GCA_905237065.2. As expected, we found one region on chr.09 that showed complete identity to the TRB constant domain sequence published by (15). This region also contained sequences with high sequence identity to variable, diversity and joining genes published in trout. However, a huge surprise was the fact that we also found a region of chr.01 that matched many rainbow trout variable and joining sequences.

Ten salmonid TRBV subgroups (V1-V10) were already identified in trout (17) and we follow that nomenclature. We introduce TRB01 for genes originating from the chr.01 region and TRB09 for those from the chr.09 region. Constant domains (C) are then denoted TRB01C and TRB09C, variable domains (V) are TRB01V and TRB09V, diversity genes (D) are TRB01D and TRB09D while joining genes (J) are TRB01J and TRB09J. Additional numbering is done separately for the two transcriptional orientations within each region in a V-D-J-C direction starting with genes encoded by the forward strand and then starting fresh with numbering on the reverse strand adding R for reverse for each gene. Variable genes with internal stop codons are shown with a P for pseudogene extension while variable sequences with expressed support are shown using the extension F for functional. The remaining variable genes contain open reading frames and are potentially expressed in other animals or under different conditions.

Atlantic salmon TRB data generated here will be implemented in the IMGT database and thus be available to the scientific community. Nomenclature suggested here will be submitted for approval by the International Union of Immunological Societies (IUIS) Nomenclature Committee (NOM) Immunoglobulins (IG), T cell receptors (TR) and major histocompatibility (MH) SubCommittee (https://iuis.org/committees/nom/immunoglobulins-ig-T cell-receptors-tr-and-major-histocompatibility-mh-nomenclature-sub-committee/) (44) prior to being accepted as the official nomenclature.

### Genomic Organization of the Duplicate Atlantic Salmon TRB Regions

A unique and surprising finding was the two duplicate T cell receptor beta regions residing on two different chromosomes, where both regions contained all the expected V-D-J-C genes for functionality. Due to the fourth whole genome duplication (4WGD) that occurred in salmonids approximately 94 million years ago, many genes and regions exist in duplicate. This is also the case for the T cell receptor beta region with one TRB region on chr.01 and a duplicate region on chr.09. These regions on chr.01 (1-6.8 Mb) and chr.09 (43-48 Mb) are homology blocks originating from the 4WGD event (45).

The Atlantic salmon TRB sequence published by Hordvik et al. (15) matched the constant gene sequence on chr.09 (TRB09), while there is no published support for the TRB01 region. Other Atlantic salmon TR genes are encoded on chr.20 [TR gamma (46),] and chr.14 [TR alpha/delta (32),]. Further support for functional duplicate Atlantic salmon TR beta regions is provided in detail below.

**Figure 1:**
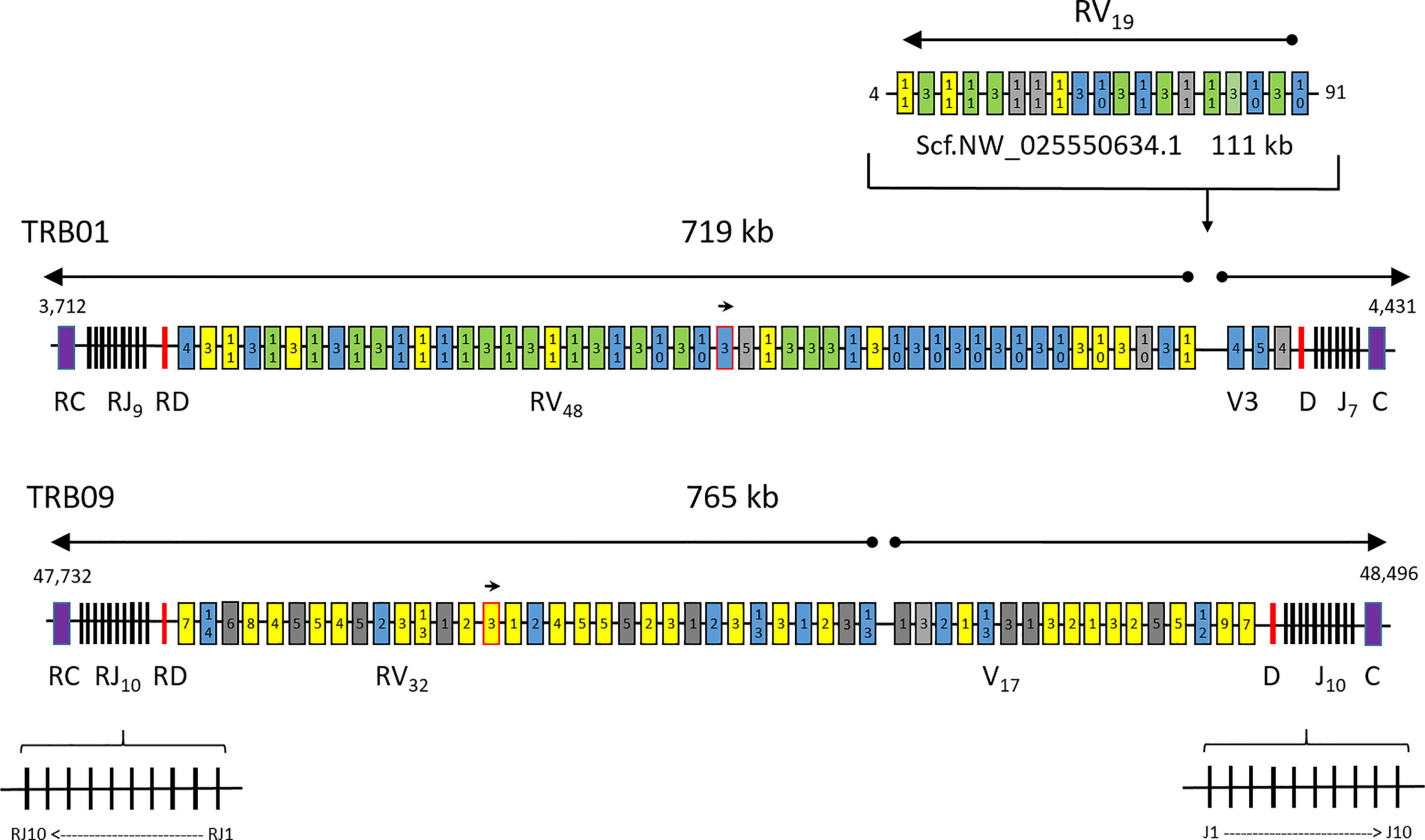
Atlantic salmon TRB chromosomal regions. Schematic overview of the T cell receptor beta loci present on chr.01, chr.09 and the unplaced scaffold with regional positions shown on each side. The regions of 719, 765 and 111 kb are not draw to scale. Constant genes (C) are shown as purple boxes, joining genes (J) as black lines, diversity genes (D) as red lines, variable genes (V) with expressed support as yellow boxes, variable genes with internal stop codons as grey boxes and bona fide variable genes without expressed support as blue boxes. Expressed sequences with complete identity to several variable genes are shown as green boxes. Variable gene subgroup is shown with numbers inside each box. The unplaced scaffold belongs in the chr.01 region, but may or may not represent another haplotype. A small arrow shows the TRBV gene in opposite orientation of transcription in the locus on both chromosomes while large arrows above the regions show transcriptional direction. The orientation of individual joining genes is only shown for the TRB09 region, but the same principle also applies for joining genes on chr.01.

The region on chr.01 (TRB01) comprises 719 kb while the duplicate region residing on chr.09 (TRB09) comprises 765 kb (**Figures 1**, **SF1.1**). Both regions contain two complete V-D-J-C sets in opposite transcriptional orientations organized in a head-to-head orientation, here defined as the forward and reverse transcriptional unit of TRB01 and TRB09. Within the TRB01 and the TRB09 regions, the constant domains have identical amino acid (aa) as well as nucleotide sequences, but there is only 67% aa identity between the TRB01C/RC and TRB09C/RC sequences (**SF1.2**). The TRB09C/RC domains are encoded by four exons while the TRB01C/RC domains are encoded by three exons.

**Figure 2:**
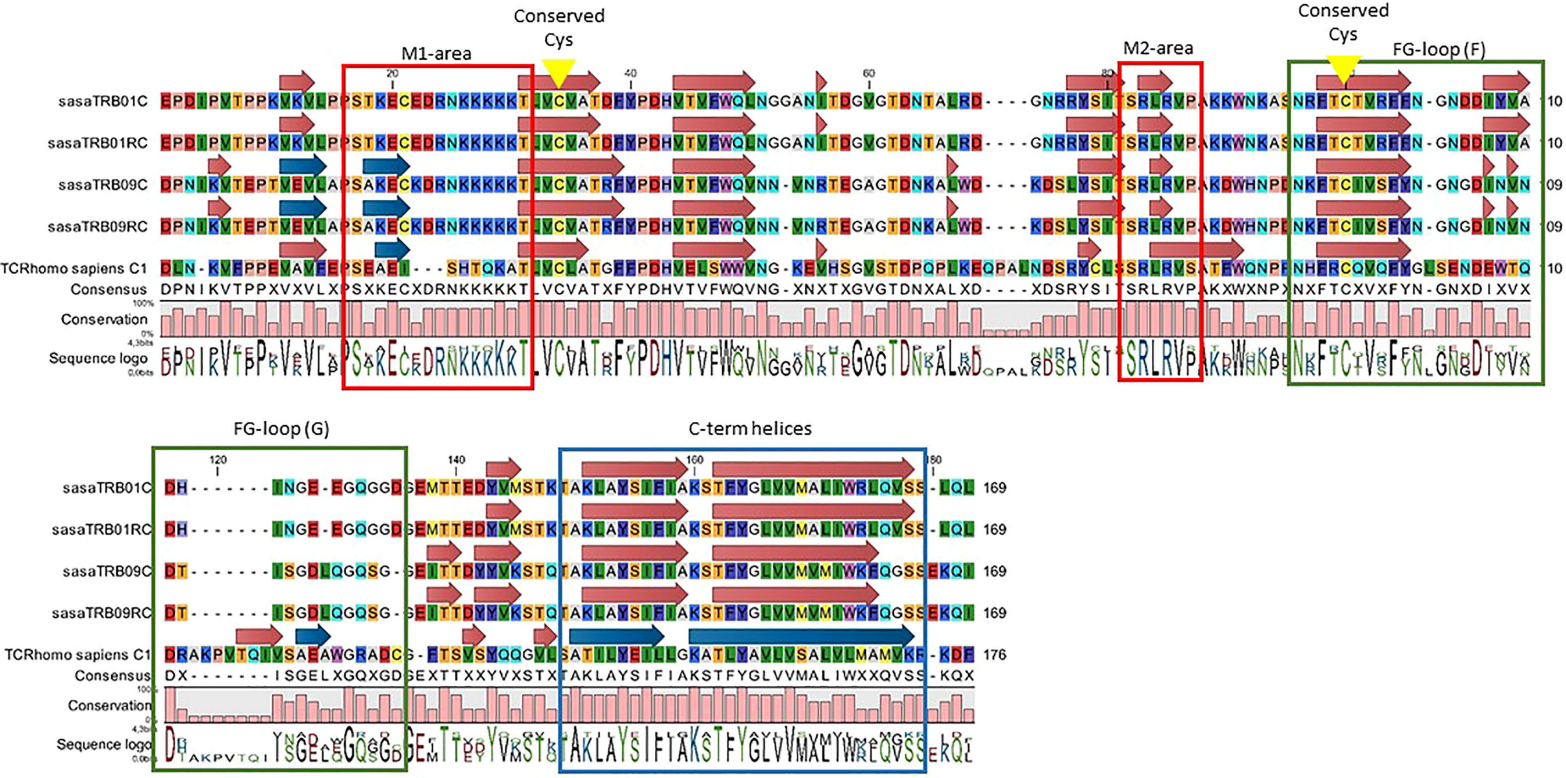
Alignment of deduced amino acid sequences from the TRBC regions from Atlantic salmon (TRB01C, TRB01RC, TRB09C, TRB09RC) with human TRBC1 (Ensembl ID ENST00000633705.1). Boxes denote regions with reported functions in signalling triggered by peptide-MHC complexes. Motifs M1 and M2 (red frames) reported in (47), FG loop (green frames) functions (48), and C-terminal helices (blue arrows and frame). Conserved cysteines are marked by yellow arrows.

Comparing the protein motifs of the TRB constant regions from Atlantic salmon with the human TRBC1 sequence (NCBI Accession # P01850.4) shows partial conservation (**Figure 2**). All four Atlantic salmon TRBC-regions are 7 aa shorter than the human TRBC1, and contain two larger gaps of 4-5 and 7 aa in addition to one 3 aa insert and some single amino acid gaps and inserts. The overall hydrophobicity profile is similar, except that all Atlantic salmon TRBC sequences contain a stretch of five lysine’s in the N-terminal part of the sequence that is lacking in human TRBC1. The cysteins involved in bridging TRA/TRB are conserved, whereas the two C-terminal helices shown to be involved in TRA interaction and strengthen antigen-dependent activation are not (49). Neither the elongated, solvent-exposed FG-loop of human TRBC1, in humans involved in antigen-triggered TCR signalling and T cell development, is conserved (48). In Atlantic salmon this region is completely disrupted by the 7 aa gap. A study on X-ray structures of the free and peptide-MHC-bound forms of the T cell receptor have previously identified two motifs of particular importance for the structural alterations involved in antigen-dependent signal transduction (M1 and M2). The first motif is completely disrupted by a 3 aa insertion and the specific Atlantic salmon lysine pentamer, but the second SSRLRV motif is completely conserved (47). Although amino acid identity between TRB01C and TRB09C is only 67%, constant domains in both regions share or lack similar features when compared to the human TRBC sequence. Thus, Atlantic salmon TRB01C/RC and TRB09C/RC should be expected to have quite similar functions.

**Figure 3:**
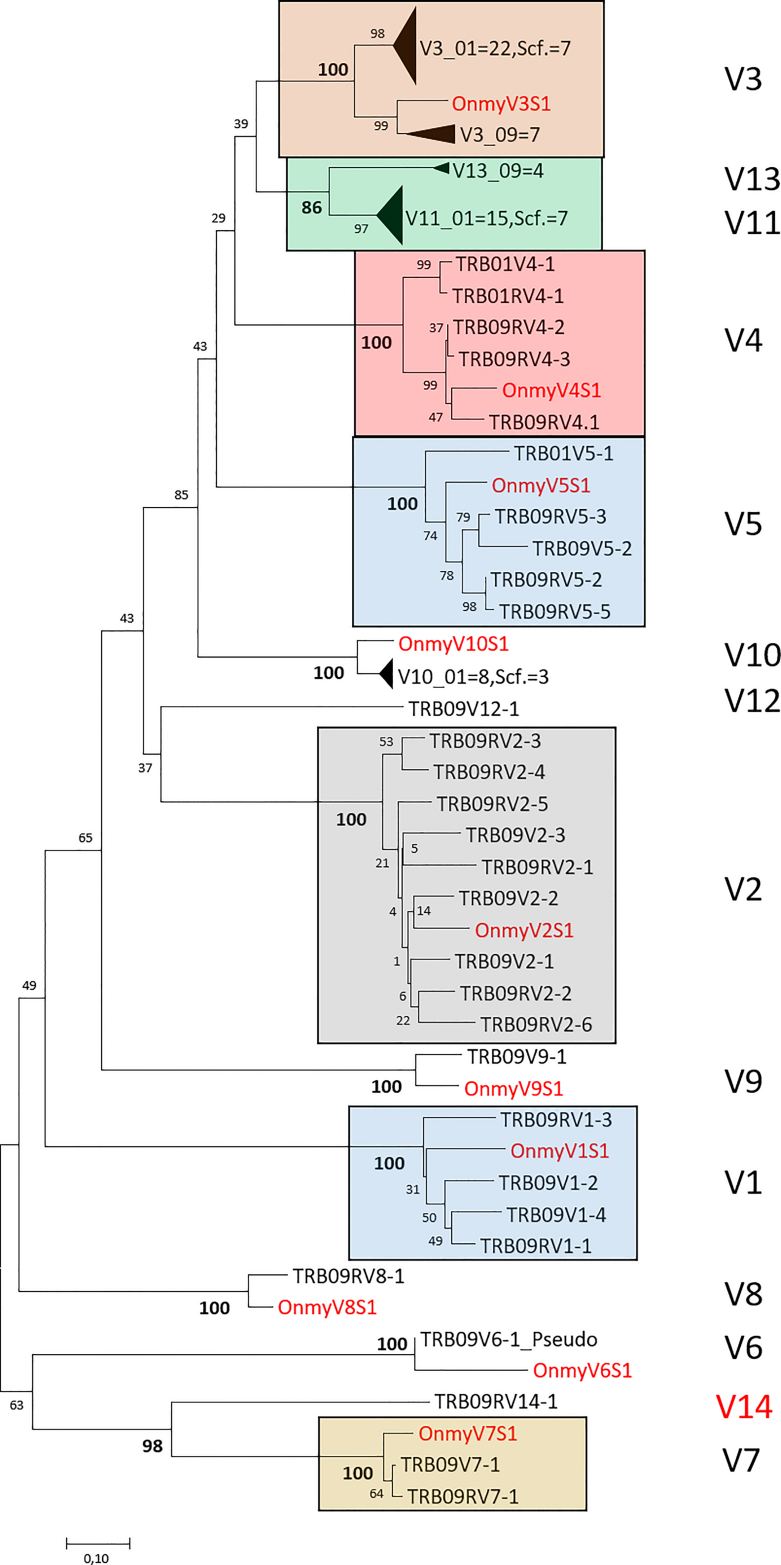
Phylogeny of deduced amino acid sequences for TRB variable (V) subgroup members from rainbow trout and Atlantic salmon. Large clusters are collapsed showing only number of V sequences originating from chr.01 (01), chr.09 (09) or the unplaced scaffold (scf.) where for instance V3_01 = 22, Scf. = 7 indicates that there are 22 V3 sequences from chr.01 and 7 V3 sequences from the unplaced scaffold in this collapsed cluster. Only one representative rainbow trout (Onmy) sequence is used per variable subgroup (17). The Atlantic salmon TRB09V6 is the only pseudogene sequence included in the tree. The optimal tree with the sum of branch length = 9.56583678 is shown. The evolutionary distances were computed using the Poisson correction method (50) and are in the units of the number of amino acid substitutions per site. The analysis involved 113 amino acid sequences with a total of 113 positions in the final dataset. Rainbow trout sequences are shown using red font and large V clusters are shown using colored boxes with subgroup category on the right hand side. GenBank accession numbers for rainbow trout variable sequences are: V1S1 (U18123), V2S1 (U18124), V3S1 (U18125) (16), V4S1 (AY135385), V5S1 (AY135386), V6S1 (AY135387), V7S1 (AY135389), V8S1 (AY135390), V9S1 (AY135391), V10S1 (AY135392) (17).

**Figure 4:**
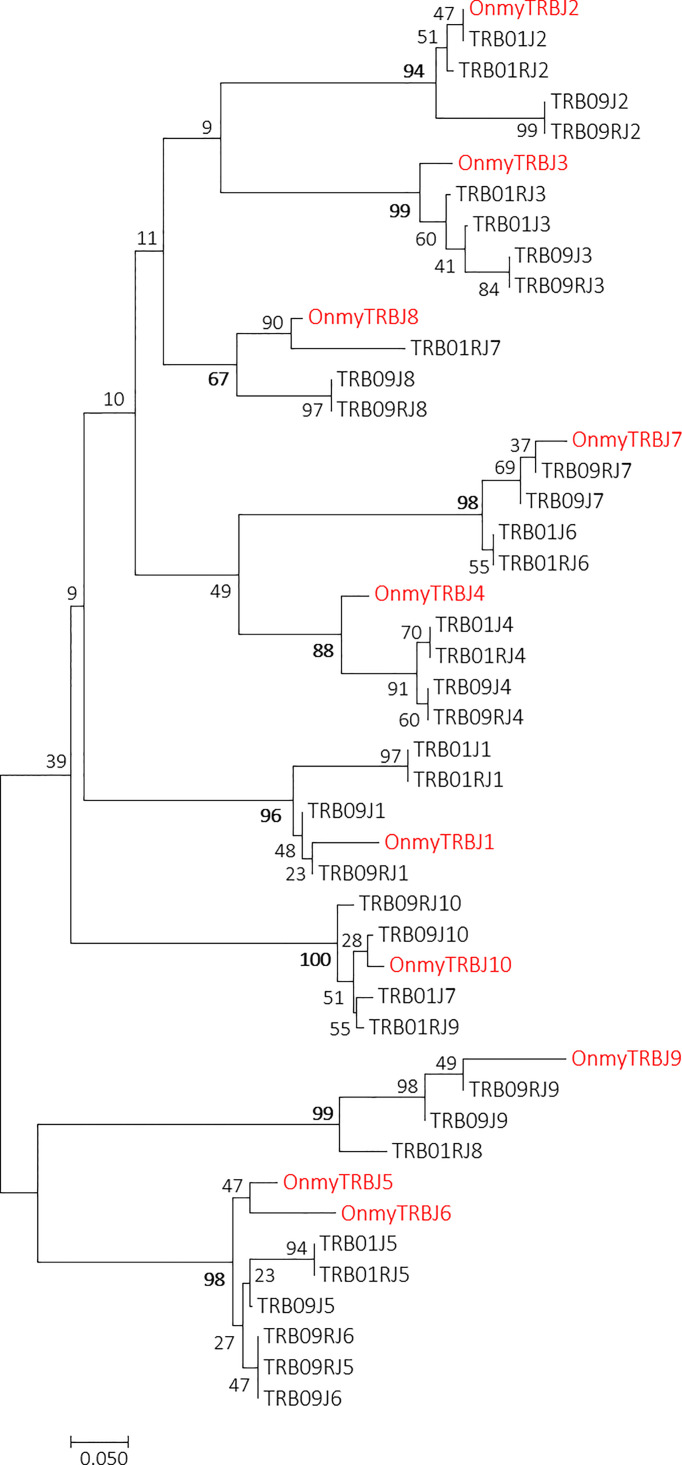
Phylogeny of rainbow trout and Atlantic salmon TRBJ nucleotide sequences. GenBank accession number for the rainbow trout sequences is U97590 (14). The optimal tree with the sum of branch length = 3.25637896 is shown. The evolutionary distances were computed using the Maximum Composite Likelihood method (51) and are in the units of the number of base substitutions per site. The analysis involved 46 nucleotide sequences. Codon positions included were 1st+2nd+3rd+Noncoding. There were a total of 55 positions in the final dataset. Rainbow trout sequences are shown using red font.

Phylogenetic trees of all rainbow trout and Atlantic salmon joining and variable gene sequences support the two regions as both encoding bona fide TRB genes. Most Atlantic salmon TRBV sequences from both regions cluster with corresponding trout TRBV subgroup sequences with high bootstrap values (**Figure 3**). A similar convincing clustering is seen for rainbow trout and Atlantic salmon TRBJ sequences (**Figure 4**). Genomic location of the rainbow trout sequences is currently unknown so further comparisons must wait comparative data.

In the TRB01 region, there are 51 variable genes where 48 are encoded in the reverse transcriptional unit and only three in the forward transcriptional unit (**Figure 1**; **Table 2**; **SF1.1**; **SF1.3**). Three of these 51 TRBV genes are pseudogenes. This region lacks variable genes belonging to V1, V2, V6, V7, V8 and V9 subgroups (17). On the other hand, there are abundant numbers of V3 and V10 genes in addition to a new subgroup here defined as V11, counting 22, 9 and 15 members respectively. There are also three TRB01V4 and two TRB01V5 subgroup genes in this region.

**Table 2:** Number of variable subgroup members per region.

Variable subgroup	Chr.01	Scaffold	Chr.09	Total
V1	–	–	8 (4)	8 (4)
V2	–	–	9	9
V3	22	7	10 (3)	39 (3)
V4	3 (1)	–	3	6 (1)
V5	2 (1)	–	8 (4)	10 (5)
V6	–	–	(1)	(1)
V7	–	–	3	3
V8	–	–	1	1
V9	–	–	1	1
V10	9 (1)	3	–	12 (1)
V11	15	9 (3)	–	24 (3)
V12	–	–	1	1
V13			4	4
Total	51 (3)	19 (3)	49 (12)	119 (18)

Scaffold sequence is NW_025550634.1. Number of pseudogenes are in parenthesis.

In the TRB09 region, we found a total of 49 variable genes, 32 encoded in the reverse transcriptional unit and 17 in the forward transcriptional unit (**Figure 1**; **SF1.1** and **SF1.3**). Twelve of these genes had internal stop codons and were thus defined as pseudogenes. The number of variable genes and pseudogenes per category in each of the duplicate regions are summarized in **Table 2**. We did not find any match to the rainbow trout V10 gene in this region and only single V8 and V9 genes. A single gene matching the trout V6 sequence was a pseudogene. Adding to the ten variable subgroups defined in trout we also found sequences here defined as V12, V13 and V14 subgroups, where V12 and V14 were single copy, while the V13 subgroup was represented by four members.

The V11 gene sequences identified on chr.01 cluster with convincing bootstrap values to sequences defined as V13 on chr.09 (**SF2**), and are most likely paralogues originating from the 4WGD. Despite their presumed common ancestry, IMGT nomenclature rules requires a sequence identity of 75% at the nucleotide level between gene sequences within a subgroup. A similar situation is seen for the TRB09V7-1/TRB09RV7-1 and TRB09RV14-1 sequences, which are clearly related, but share less than 75% sequence identity and thus belong to different subgroups. The remaining variable subgroups all comply with IMGT nomenclature rules for their assigned members.

In the middle of the reverse transcriptional unit on chr.01, there is a single gene that is encoded by the forward strand (**SF1.1**; TRB01RV3-12). This is matched by a single gene on chr.09 that is also encoded by the forward strand in the middle of the reverse transcriptional unit (**SF1.1**; TRB09RV3-5). Such an organization of a single TRBV3 subgroup gene on both chromosomes suggests this translocation occurred prior to the whole genome duplication event 94 MYA. It will be interesting to see if this organization also exists in other salmonids.

An additional 19 variable genes are found on an unlinked scaffold (NW_025550634.1) with seven V3, three V10 and nine V11 subgroup genes (**Figure 1**; **SF1.1**; **SF1.3**). As their genomic location is currently unknown, they received an extension of S as exemplified by Scf.TRBV3S1. Based on phylogenies, these scaffold sequences belong to the TRB01 cluster as scaffold V3, V10 and V11 sequences form clusters with TRB01V sequences while the TRB09V sequences form separate clusters (**SF2**). If these variable sequences represent a haplotypic variant of the TRB01 region in this diploid animal, which has not been excluded from the assembly, or a true extension of the TRB01 region remains to be established.

We found leader sequences for all but one variable sequence i.e. TRB09V11-3 and some pseudogenes (**SF1.1**, **SF1.4**). Leader sequences group according to variable subgroups with the exception of TRBV4 and TRBV5 subgroup members that showed considerable leader sequence diversity. Variable subgroup 3 and 11/13 leader sequences share 11 of 16 amino acids supporting the weak phylogenetic clustering seen for their variable gene sequences (**SF2**). We have not investigated if leader sequence variation also reflects promoter differences or expression differences.

Ten trout TRBJ genes cluster with all the joining gene sequences identified in the TRB01 and TRB09 regions [ (14), **Figure 4**; **SF1.5**]. In the salmon TRB09 region, ten salmon J genes reside in both transcriptional orientations where some are completely identical duplicates organised in an inverted orientation (**Figure 1**). TRB09J2, -J3, -J4, -J6, and -J8 have complete sequence identity to their inverted counterpart i.e. TRB09RJ2, -RJ3, -RJ4, -RJ6 and -RJ8. The TRB01 region has a similar organization with nine genes encoded by the forward strand, but only seven genes encoded in the reverse orientation. Here the TRB01J1, -J4, -J5 and -J6 are completely identical to the reverse J genes TRB01RJ1, -RJ4, -RJ5 and -RJ6. The TRB09J8 and TRB09J9 genes have been lost on the forward strand of chr.01, but are retained on the reverse strand where TRB09J8/RJ8 cluster with TRB01RJ7 and TRB09J9/RJ9 cluster with TRB01RJ8. Further comparisons between Atlantic salmon and rainbow trout joining genes need to await the genomic organization of those in rainbow trout.

A diversity element resides in duplicate in each region in between the joining and variable genes. There is 100% sequence identity between the duplicate TRB01D/RD and TRB09D/RD diversity sequences and also to the core diversity sequence found in rainbow trout [ (14), **SF1.6**].

### Expressed Support for Dual TRB Regions

To evaluate functionality of the duplicate Atlantic salmon TRB regions, we amplified variable subgroup members with unique reverse primers located within the TRB01C and TRB09C constant genes (**Table 1**). To ensure a strong and diverse expression of the receptor we used a mixture of cDNA from spleen of eight Atlantic salmon animals sampled eight weeks after PRV challenge. At this time point the animals had developed a cytotoxic T cell driven heart inflammation (37). We used two Nano Illumina runs where run statistics for each variable subgroup amplicons are shown in **SF1.7**. Unique combined and collapsed sequences per variable subgroup are provided in supplementary file 3 (**SF3**). Our primers amplified members from their intended variable subgroups with the exception of V3 and V11/V13 primers where amplicons contained a mixture of V3 and V11/V13 gene sequences.

There was a large variation in the number of transcripts amplified by the different forward and reverse primers (**SF1.7**). This may reflect true differences between expression levels for the various variable subgroups or it could originate from differences in primer amplification efficiencies. Testing primer efficiencies is not viable with primers amplifying either single or multiple genes and we did not have access to samples with known expression of the variable gene subgroups. For instance, lack of amplification of the V12 gene could indicate that this gene was not expressed in our eight chosen animals, or alternatively that this gene is only expressed under certain conditions. We did find an expressed V12 match in the NCBI database (GBRB01000905.1) supporting its functionality. For the TRB01 region, most reads were identified for the V3/V11 subgroup, with 3862 unique collapsed sequences each supported by 20 reads or more (**SF3**). We did not test the expression levels of the TRB01V4 and V5 genes and the TRB01V10 subgroup only provided few reads. A larger set of animals exposed to many different conditions need to be tested before the functional status of each variable gene can be defined.

As our primary goal was to acquire functional support for the two genomic regions, we focused on amplicons with open reading frames thus ignoring sequences containing internal stop codons. Although our expressed sequences display 100% sequence identity to many of the genomic genes, we may have missed out on some sequence variation in regions not amplified by our primers as our forward primers all reside within the 5’ end of the variable genes.

For the TRB09V specific amplification we found expressed support for two out of eight TRB09V1, five out of nine V2, seven out of ten TRB09V3, all three TRB09V4, four out of eight TRB09V5, both TRB09V7, one out of four TRB09V13 and the single TRB09V8 and TRB09V9 genes i.e. 26 of 49 variable domains had expressed support (**Figure 1**; **SF1.8**; **SF3**). We did not acquire any fragments for the primers V2.F1, V10.F1 and V12.F (**Table 1**). As the genomic organization showed no V10 subgroup members in the TRB09 region, lack of fragments for the V10.F1 primer was expected. The genomic TRB09RV3-2_P sequence had an internal stop codon in the genomic sequences, but was supported by bona fide sequences in our tested animals (**SF1.8**,w **SF3**). This discrepancy could be caused by genomic sequencing errors or it could represent true haplotypic variation in pseudogene status. We also found many variable gene sequences with no match in the investigated genome (**SF3**), which most likely represents haplotypic or allelic variants present in the eight tested animals.

**Figure 5:**
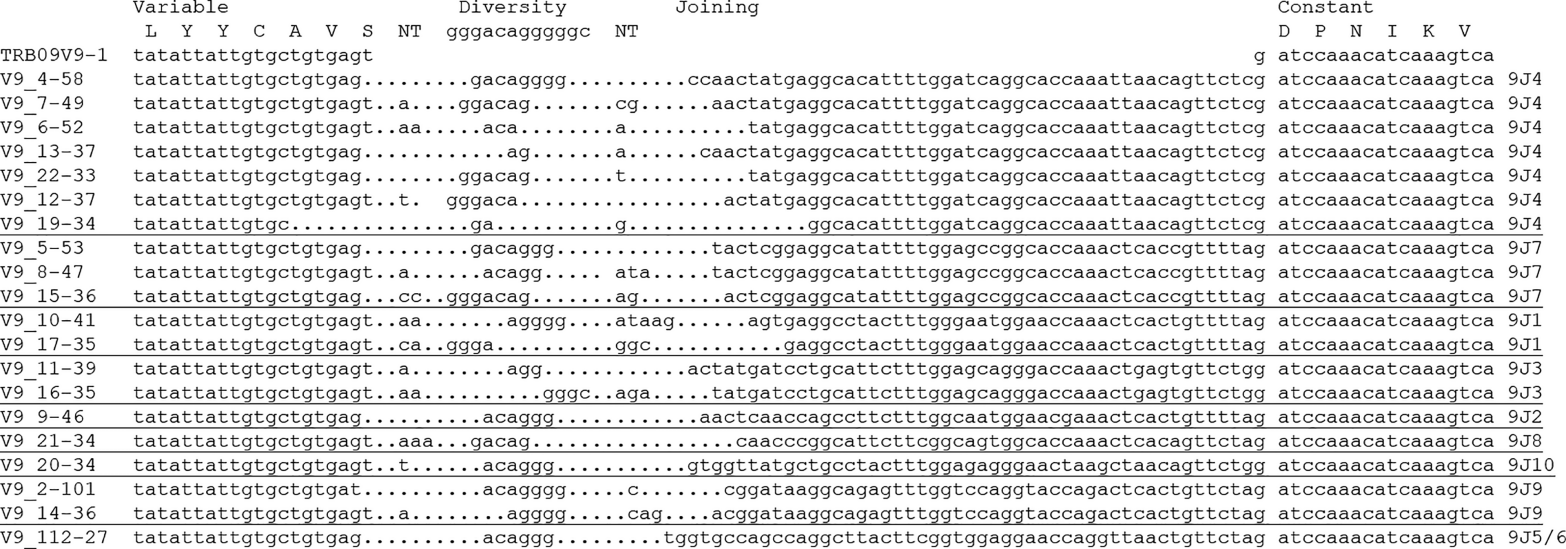
Alignment of CDR3 region for selected expressed TRB09V9 sequences. Variable, diversity, joining and constant regions are shown above the alignment. Non-genome nucleotide insertions are shown in the regions denoted NT. Dots indicate missing sequence. Individual TRB09 joining elements used are shown on the right hand side where we cannot decipher between 9J5/9J6 as they are completely identical. Names of expressed reads, for instance V9_4-58, indicate Variable subgroup 9, collapsed read number 4 supported by 58 sequences with 100% nucleotide sequence identity.

We found expressed support for all of the joining genes in the TRB09 region and we did not find any joining gene sequences not encoded by this region (**Figure 5**; **SF3**). We assume that the joining genes used were the ones in the same transcriptional orientation, but with complete sequence identity between joining genes in both transcriptional directions this is difficult to ascertain. A complete identity between the TRB09J6, TRB09RJ5 and TRB09RJ6 genes also makes it impossible to decipher which of these genes are used. Some variable domains were found to combine with all joining genes in their transcriptional direction while others were only found with one or a few joining genes. This is more a question of how many transcripts of a given variable domain was sequenced than actual variation between how variable genes used joining gene sequences. A more thorough study is needed to detect frequency differences in use of joining genes for the individual variable genes.

An added variation was seen in the variable-diversity and diversity-joining junctions where single or multiple nucleotides are inserted or deleted in the V-D and D-J junctions of the CDR3 region (**Figure 5**). Such junctional changes are common for T cell receptors and greatly increases TRB ability to recognize a multitude of peptides (9, 35, 52).

Although the variable TRB01 and unplaced scaffold (NW_025550634.1) gene sequence phylogenies suggest that the scaffold sequence originates from the TRB01 region (**SF2**), added support came from the expressed sequences. The unique TRB01RJ7 and TRB01RJ8 sequences were used by many of the expressed scaffold sequences, which then provides evidence for both origin as well as orientation of this scaffold (**Figure 1**; **SF1.9**). As exemplified by expressed sequences matching scf.TRBRV11S9, all nine TRB01 joining genes are used. If this scaffold represents an addition to the TRB01 region or a different haplotype remains to be established, since the sequenced genome originates from a normal diploid animal. Many TRB01 and scaffold variable genes have completely identical amino acid as well as nucleotide sequences (**SF1.10**; **SF2**), making it difficult to match transcribed sequence to unique genomic gene. Such examples are for instance TRB01RV3-15, TRB01RV3-17, TRB01RV3-18, TRB01RV3-21, scf.TRBRV3S3, scf.TRBRV3S4, scf.TRBRV3S6 and scf.TRBRV3S7 having completely identical amino acid as well as nucleotide sequences. Due to the complete sequence identity of the TRB01 and scaffold sequences we only found unique match to 11 of 51 variable genes in the TRB01 region and only 3 of 19 genes in the scaffold region (**Figure 1**; **SF3**). Such identical genes on both the scaffold as well as the TRB01 region insinuates that the scaffold is indeed a haplotype version of the TRB01 region.

### Functional Implications

A duplicate functional TRB region in Atlantic salmon is surprising. Why this has not been shown for any other salmonid most likely relies on the problems in assembling regions with multiple highly similar gene sequences. We have unsuccessfully tried to characterize the TRB region in older versions of the Atlantic salmon. Now, a new generation of Atlantic salmon genomes emerge using improved sequencing technologies such as long-range nanopore, thus increasing the scaffold numbers and N50 scaffold length from 839,389 scaffolds and N50 of 592,762 bp (Sally, NCBI genome accession number GCA_000233375.4), to 4,222 scaffolds and N50 scaffold length of 28,058,890 bp (NCBI genome accession number GCA_905237065.2).

Constant domains from both TRB01 and TRB09 (**SF1.2**) are also present in other salmonid genomes. We found transcribed match to both TRB01 as well as TRB09 constant domains in chinook salmon, rainbow trout, brown trout and brook trout (**SF1.11**). Preliminary data show that a similar genomic TRB organization is also found for other salmonids (manuscript in prep.), where the salmonid tetraploidy may have allowed these salmonids to choose different regional rearrangements. Chromosomal rearrangements is exemplified by the North American Atlantic salmon genome GCA_021399835.1. According to the information provided by NCBI (https://www.ncbi.nlm.nih.gov/assembly/GCA_021399835.1), there has been a rearrangement of chr.01 in North American Atlantic salmon where the p arm containing the TRB01 region has translocated to chr.23 (**SF1.12**).

**Figure 6:**
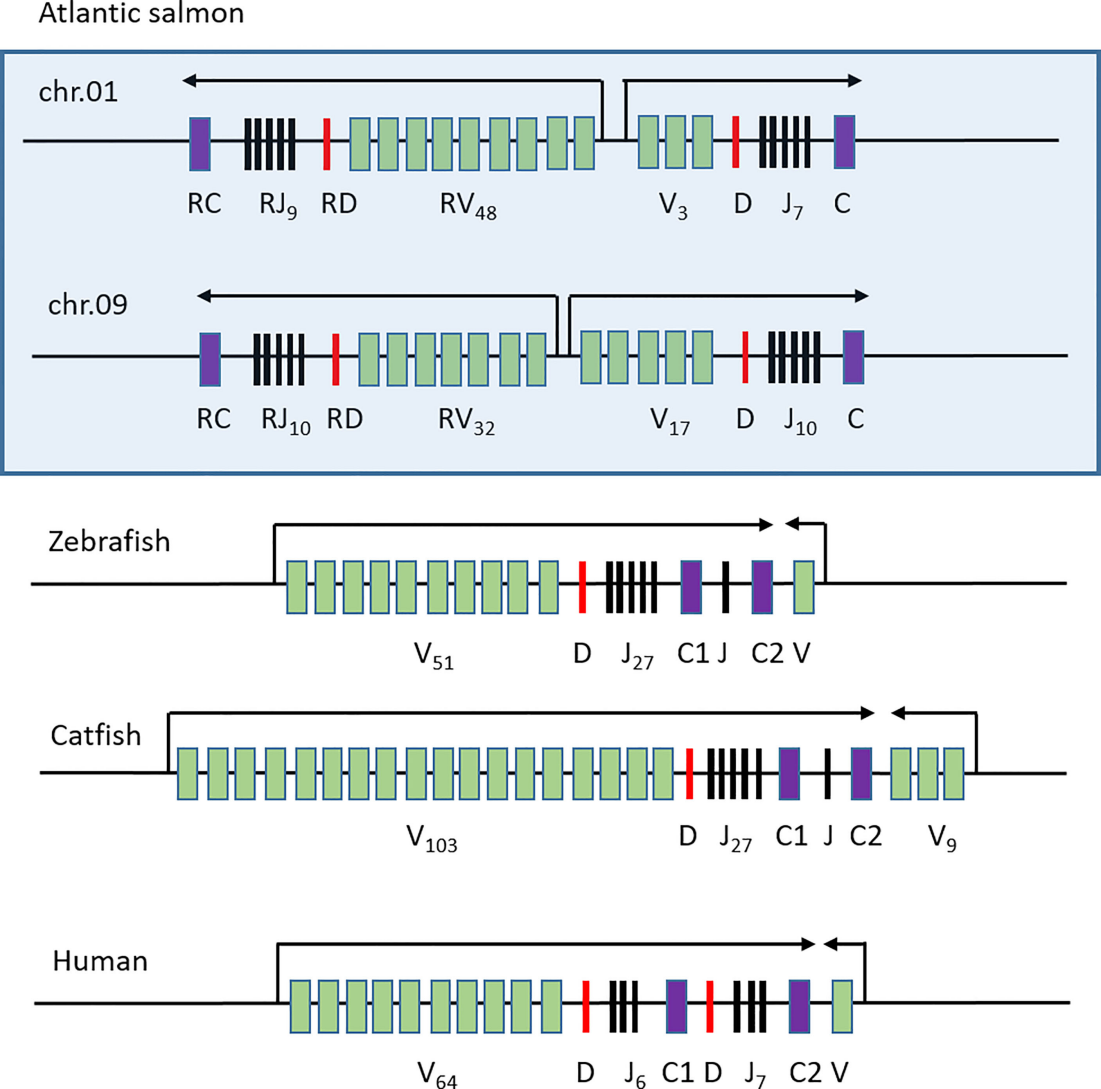
Comparative genomic organization of TRB regions. Genomic organization of the Atlantic salmon TRB01 and TRB09 regions (boxed) compared to those of zebrafish (35), catfish (36) and human (52). Constant genes are shown as purple boxes, joining genes as black lines, diversity genes as red lines and variable genes as green boxes. Transcriptional orientation is shown above with arrows and number of individual genes are shown as subscript.

The genomic organization of the TRB regions in Atlantic salmon differs from that found in zebrafish and catfish (**Figure 6**). These teleosts have one main transcriptional orientation with variable, diversity, joining and dual constant domains followed by one or a few variable domains in the reverse orientation (35, 36). Humans also have an organization of the TRB region, which resembles that of zebrafish and catfish (52). Both zebrafish and catfish are ostariophysian species so the fact that they have similar TRB gene organizations is not surprising (53). Atlantic salmon is a salmonid which separated from the ostariophysians approximately 250 million years ago. The additional unique whole genome duplication has enabled a different organization of this salmon TRB region.

The Atlantic salmon TRA/TRD region was shown to have an unprecedented 292 variable alpha/delta genes and 123 joining genes, which allows for a very diverse T cell receptor alpha chain (32, 33). Adding the variable and joining genes in the duplicate TRB regions provides Atlantic salmon with the most diverse TRA/TRB opportunity of any vertebrate studied so far accommodating for a vast MHC-peptide diversity. Perhaps salmonid cellular immunity is more advanced than we have imagined.

The unique salmonid whole genome duplication event provided these species with the opportunity to acquire both whole duplicate functional regions as seen here, but also a myriad of duplicate functional genes as for example seen for Atlantic salmon genes involved in the antigen presentation pathway (54). Here Atlantic salmon has retained some functional copies from each of the 2WGD, teleost specific 3WGD as well as the unique salmonid 4WGD. Having a tetraploid past has enabled Atlantic salmon to acquire new or sub-functions of the duplicate genes and this may also be the case for the TRB regions. Only 67% sequence identity between the constant domains of chr.01 and chr.09 shows a rapid diversification of this TRB01 region as opposed to the 82-90% identity observed between other duplicate genes (45, 54, 55). Variable subgroup 11 and 13 members on chr.01 and 09 also support a more rapid evolution of the chr.01 region. These V11 and V13 subgroup genes are clearly duplicates showing strong clustering in phylogenies (**SF2**), but only have amino acid sequence identity of 70-76%. Presence of many variable genes with complete sequence identity in the TRB01 region also supports this region as changing more rapidly than the TRB09 region i.e. many recent gene duplications.

Although speculative, the duplicate TRB regions could have evolved into specialised MHCI and MHCII restricted regions. Alternative speculations would be that these two regions have specialised functions in different tissues e.g. mucosal immunity versus systemic immunity or are expressed on different subsets of T cells. The functional implications of a duplicate functional TRB region needs to be addressed in future studies.

## Conclusion

The unique 4WGD of salmonids has provided these organisms with a unique opportunity to acquire genes with new or subfunctions for the duplicates. This has provided at least Atlantic salmon with duplicate T cell receptor beta regions where each region contains some common and some unique variable domain subgroups. This could allow each region to serve some common and some unique biological roles. Adding the tremendous T cell receptor alpha diversity makes Atlantic salmon the species with highest TR diversity among vertebrates. Having access to curated TR alpha and beta genes alongside advances in high throughput sequencing now allows us for the first time to perform in-depth studies of adaptive immune responses in Atlantic salmon.

## Data Availability Statement

The datasets presented in this study can be found in online repositories. The names of the repository/repositories and accession number(s) can be found below: NCBI SRA under the BioProject accession number PRJNA814480.

## Ethics Statement

The animal study was reviewed and approved by Norwegian Animal Research Authority.

## Author Contributions

UG designed the study, analysed the data and wrote the initial manuscript. MD provided the samples and performed an analysis of the constant domain. AS performed the bioinformatics. CB performed the Illumina sequencing. ML amplified all PCR fragments and made the sequenced libraries. All authors contributed to the article and approved the submitted version.

## Funding

This work has been carried out as part of the Norwegian Veterinary Institute strategic program “BIODIRECT: Biomarkers and bioassays for future research and diagnostics”, which is funded via institutional base funding from the Norwegian Research Council. Additional funding comes from the NRC 274635 and NRC 280847 projects.

## Conflict of Interest

The authors declare that the research was conducted in the absence of any commercial or financial relationships that could be construed as a potential conflict of interest.

## Publisher’s Note

All claims expressed in this article are solely those of the authors and do not necessarily represent those of their affiliated organizations, or those of the publisher, the editors and the reviewers. Any product that may be evaluated in this article, or claim that may be made by its manufacturer, is not guaranteed or endorsed by the publisher.
