# Supplementary material for: Tetraploid Ancestry Provided Atlantic Salmon With Two Paralogue Functional T Cell Receptor Beta Regions Whereof One Is Completely Novel
Source: Front Immunol. 2022 Jun 17;13:930312. doi: 10.3389/fimmu.2022.930312 (PMC9247247; doi:10.3389/fimmu.2022.930312)
Supplement: Supplementary file 1 [file DataSheet_1.pdf]

Supplementary file 1 (SF1). Relevant sequences and supporting information

SF1	Table of Content	Page
1.1	Location of Atlantic salmon T cell receptor beta L-V-D-J-C gene sequences	1
1.2	Alignment and sequence identity for deduced constant domain amino acid sequences from selected species	5
1.3	Alignment of deduced Atlantic salmon TRB variable domains from TRB01, TRB09 and scaffold region	6
1.4	Alignment of deduced Atlantic salmon leader sequences from TRB01, TRB09 and scaffold regions	10
1.5	Alignment of rainbow trout and Atlantic salmon TRB joining sequences	12
1.6	Alignment of diversity sequences from rainbow trout and A. salmon TRB01 and TRB09 regions	13
1.7	Nano Illumina statistics for sequencing of expressed Atlantic salmon TRB	14
1.8	Expressed support for genomic A. salmon TRB01, TRB09 and scaffold variable sequences	14
1.9	Linking scaffold NW_025550634.1 to chromosome 01	18
1.10	Alignment of selected Atlantic salmon variable TRB01 and scaffold nucleotide sequences showing 100 % sequence identity	19
1.11	Expressed support for TRB01 and TRB09 constant domain from other salmonids	20
1.12	Location of TRB01C sequences in North American Atlantic salmon	23

Rainbow trout sequences used in this document originate from de Partula et al.1995, Guerra & Charlemagne 1997, Boudinot et al. 2002 and. See main text for references.

1.1 Location of Atlantic salmon T cell receptor beta L-V-D-J-C gene sequences

Sequences with a P extension are pseudogenes with internal stop codons while extension F shown genes with expressed support. Remaining sequences are open reading frame sequences that may be expressed in other animals or under other conditions. Genomic location of leader (L-PART1 exon), variable (V-exon), Joining (J), diversity (D) and constant (C) genes are given.

T cell receptor beta (TRB) genes on Atlantic salmon chr. 01 (GCA_905237065.2; NC_059442.1) and unplaced scaffold (NW_025550634.1):

Genomic coordinates of chr.01 genes in 5’-3 transcriptional unit		
Gene Name	L-PART1 exon	V-exon
TRB01V4-1	4,392,054-4,392,131	4,392,591-4,392,899
TRB01V5-1	4,393,340-4,393,414	4,393,455-4,393,769
TRB01V4-2_P	4,405,432-4,405,509	4,405,841-4,406,074
TRB01D		4,424,601-4,424,648
TRB01J1		4,427,697-4,427,759

TRB01J2		4,427,871-4,427,942
TRB01J3		4,428,075-4,428,167
TRB01J4		4,428,278-4,428,376
TRB01J5		4,428,605-4,428,685
TBR01J6		4,429,094-4,429,165
TRB01J7		4,429,852-4,429,926
TRB01C		4,431,095-4,431,982
Genomic coordinates of chr.01 genes in 3'-5 transcriptional unit		
Gene name	L-PART1 exon (complement)	V-EXON (complement)
TRB01RV11-1_F	4,383,875-4,383,828	4,383,700-4,383,398
TRB01RV3-1	4,380,694–4,380,647	4,380,542-4,380,234
TRB01RV10-1_P	No match	4,371,083-4,370,820
TRB01RV3-2_F	4,370,064-4,369,996	4,369,912-4,369,604
TRB01RV10-2_F	4,363,203-4,363,141	4,363,042-4,362,746
TRB01RV3-3_F	4,361,990-4,361,943	4,361,838-4,361,530
TRB01RV10-3	4,354,450-4,354,388	4,354,289-4,353,993
TRB01RV3-4	4,353,237-4,353,190	4,353,085-4,352,777
TRB01RV10-4	4,342,885-4,342,823	4,342,724-4,342,428
TRB01RV3-5	4,341,672-4,341,625	4,341,520-4,341,212
TRB01RV10-5	4,332,638-4,332,576	4,332,477-4,332,181
TRB01RV3-6	4,331,425-4,331,378	4,331,273-4,330,965
TRB01RV10-6	4,323,975-4,323,913	4,323,814-4,323,518
TRB01RV3-7	4,322,762-4,322,715	4,322,610-4,322,302
TRB01RV10-7	4,313,432-4,313,370	4,313,271-4,312,975
TRB01RV3-8_F	4,310,781-4,310,734	4,310,629-4,310,321
TRB01RV11-2	4,301,433-4,301,386	4,301,152-4,300,850
TRB01RV3-9	4,296,620-4,296,573	4,296,468-4,296,160
TRB01RV3-10	4,293,779-4,293,732	4,293,627-4,293,319
TRB01RV3-11	4,290,937-4,290,890	4,290,785-4,290,477
TRB01RV11-3_F	4,288,716-4,288,669	4,288,479-4,288,195
TRB01RV5-1_P	No match	4,280,952-4,280,680
TRB01RV3-12 (FWD)	4,266,119-4,266,166	4,266,271-4,266,579
TRB01RV10-8	3,893,282–3,893,220	3,893,121-3,892,825
TRB01RV3-13	3,892,239-3,892,192	3,892,087-3,891,779
TRB01RV10-9	3,890,422-38,903,60	3,890,261-3,889,965
TRB01RV3-14	3,889,379-3,889,332	3,889,227-3,888,919
TRB01RV11-4	3,887,706-3,887,659	3,887,437-3,887,153
TRB01RV3-15	3,882,592-3,882,545	3,882,439-3,882,131
TRB01RV11-5	3,875,665-3,875,591	3,875,421-3,875,119
TRB01RV11-6_F	3,870,997-3,870,950	3,870,782-3,870,498
TRB01RV3-16	3,865,938-3,865,891	3,865,785-3,865,477
TRB01RV11-7	3,856,523-3,856,476	3,856,310-3,856,026
TRB01RV3-17	3,851,488-3,851,441	3,851,335-3,851,027
TRB01RV11-8	3,844,565-3,844,491	3,844,323-3,844,021
TRB01RV11-9	3,839,770-3,839,723	3,839,501-3,839,217
TRB01RV11-10_F	3,834,878-3,834,831	3,834,663-3,834,379
TRB01RV11-11	3,828,008-3,827,961	3,827,812-3,827,510
TRB01RV3-18	3,822,954-3,822,907	3,822,801-3,822,493
TRB01RV11-12	3,816,026-3,815,952	3,815,782-3,815,480
TRB01RV3-19	3,811,581-3,811,534	3,811,428-3,811,120
TRB01RV11-13	3,804,613-3,804,548	3,804,368-3,804,066
TRB01RV3-20_F	3,800,173-3,800,126	3,800,020-3,799,712
TRB01RV11-14	3,792,869-3,792,795	3,792,617-3,792,315
TRB01RV3-21	3,788,415-3,788,368	3,788,269-3,787,961

TRB01RV11-15_F	3,781,324-3,781,277	3,781,124-3,780,837
TRB01RV3-22_F	3,776,312-3,776,265	3,776,160-3,775,852
TRB01RV4-1	3,751,641-3,751,582	3,751,402-3,751,094
TRB01RD		3,725,134-3,725,087
TRB01RJ1		3,716,367-3,716,305
TRB01RJ2		3,716,193-3,716,122
TRB01RJ3		3,715,989-3,715,897
TRB01RJ4		3,715,786-3,715,688
TRB01RJ5		3,715,459-3,715,379
TRB01RJ6		3,714,942-3,714,871
TRB01RJ7		3,714,127-3,714,062
TRB01RJ8		3,713,777-3,713,706
TRB01RJ9		3,713,350-3,713,276
TRB01RC		3,712,213-3,711,729
Genomic coordinates of unplaced scaffold NW_025550634.1 genes in 3'-5 transcriptional unit		
Gene Name	L-PART1 (complement)	V-EXON (complement)
SCF.TRBRV10S1	91,017-90,955	90,856-90,560
SCF.TRBRV3S1	89,974-89,927	89,822-89,514
SCF.TRBRV10S2	88,157–88,095	87,996-87,700
SCF.TRBRV3S2	87,114-87,067	86,962-86,654
SCF.TRBRV11S1	85,736-85,689	85,468-85,184
SCF.TRBRV11S2_P	81,070-81,023	80,874-80,617
SCF.TRBRV3S3	76,013-75,966	75,860-75,552
SCF.TRBRV11S3	69,087-69,013	68,845-68,543
SCF.TRBRV3S4	58,745-58,698	58,592-58,284
SCF.TRBRV10S3	52,093-52,031	51,932-51,636
SCF.TRBRV3S5	51,049-50,981	50,897-50,589
SCF.TRBRV11S4_F	48,873-48,799	48,404-48,102
SCF.TRBRV11S5_P	43,298-43,251	43,028-42,747
SCF.TRBRV11S6_P	36,608-36,557	36,410-36,159
SCF.TRBRV3S6	31,566-31,519	31,413-31,105
SCF.TRBRV11S7	24,598-24,524	24,354-24,052
SCF.TRBRV11S8_F	19,678-19,631	19,460-19,179
SCF.TRBRV3S7	14,626-14,579	14,473-14,165
SCF.TRBRV11S9_F	5,101 -5054	4,885-4,604

Location of Atlantic salmon T cell receptor beta (TRB) genes on chr.09
(GCA_905237065. 2, NC_059450.1)

Genomic coordinates of chr.09 genes in 5'-3 transcriptional unit		
Gene names	L-PART1	V-EXON
TRB09V1-1_P	48,398,140-48,398,202	48,398,388-48,398,678
TRB09V3-1_P	48,400,807-48,400,854	48,401,027-48,401,263
TRB09V2-1	48,406,946-48,406,999	48,407,156-48,407,431
TRB09V1-2_F	48,411,891-48,411,953	48,412,140-48,412,433
TRB09V13-1	48,412,907-48,412,981	48,413,348-48,413,629
TRB09V3-2_P	48,414,812-48,414,859	48,414,916-48,415,264
TRB09V1-3_P	48,427,834-48,427,893	48,428,036-48,428,329
TRB09V3-3_F	48,429,905-48,429,952	48,430,060-48,430,362
TRB09V2-2_F	48,433,151-48,433,204	48,433,355-48,433,633
TRB09V1-4_F	48,435,685-48,435,747	48,435,934-48,436,227
TRB09V3-4_F	48,437,952-48,437,999	48,438,107-48,438,409
TRB09V2-3_F	48,442,933-48,442,986	48,443,190-48,443,468

TRB09V5-1_P	48,448,032-48,448,094	48,448,283-48,448,504
TRB09V5-2_F	48,452,611-48,452,661	48,452,779–48,453,072
TRB09V12-1	48,460,217-48,460,285	48,460,492-48,460,794
TRB09V9-1_F	48,463,790-48,463,864	48,464,539-48,464,814
TRB09V7-1_F	48,472,720-48,472,780	48,472,987-48,473,280
TRB09D		48,488,415-48,488,462
TRB09J1		48,488,957-48,489,049
TRB09J2		48,489,152-48,489,229
TRB09J3		48,489,332-48,489,444
TRB09J4		48,489,549-48,489,653
TRB09J5		48,490,070-48,490,165
TRB09J6		48,490,326-48,490,397
TRB09J7		48,490,727-48,490,810
TRB09J8		48,492,038-48,492,320
TRB09J9		48,492,584-48,492,664
TRB09J10		48,492,919-48,493,023
TRB09C		48,494,763-48,496,175
Genomic coordinates of chr.09 genes in 3’-5 transcriptional unit		
Gene names	L-PART1 (complement)	V-EXON (complement)
TRB09RV13-1	No match	48,318,267-48,317,986
TRB09RV3-1_P	48,316,433-48,316,386	48,316,219-48,315,998
TRB09RV2-1_F	48,314,588-48,314,535	48,314,398-48,314,123
TRB09RV1-1	48,296,987-48,296,925	48,296,738-48,296,445
TRB09RV3-2_F	48,294,560-48,294,513	48,294,405-48,294,103
TRB09RV13-2	48,285,590-48,285,528	48,285,012-48,284,731
TRB09RV3-3_F	48,283,847-48,283,800	48,283,692-48,283,390
TRB09RV2-2	48,279,401-48,279,348	48,279,198-48,278,926
TRB09RV1-2_P	48,266,910-48,266,848	48,266,662-48,266,369
TRB09RV3-4_F	48,264,135-48,264,088	48,263,980-48,263,678
TRB09RV2-3_F	48,257,742-48,257,689	48,257,533-48,257,261
TRB09RV5-1_P	No match	48,254,285-48,253,989
TRB09RV5-2_F	48,253,657-48,253,595	48,253,477-48,253,178
TRB09RV5-3_F	48,251,419-48,251,369	48,251,251-48,250,952
TRB09RV4-1_F	48,250,682-48,250,620	48,250,444-48,250,154
TRB09RV2-4	47,982,533-47,982,486	47,982,309-47,982,031
TRB09RV1-3_F	47,976,588-47,976,526	47,976,339-47,976,049
TRB09RV3-5_F (FWD)	47,972,414-47,972,461	47,972,569-47,972,871
TRB09RV2-5_F	47,969,418-47,969,365	47,969,214-47,968,942
TRB09RV1-4_P	No match	47,957,200-47,956,910
TRB09RV13-3_F	47,956,502-47,956,428	47,956,210-47,955,929
TRB09RV3-6_F	47,954,424-47,954,377	47,954,269-47,953,967
TRB09RV2-6	47,951,659-47,951,606	47,951,453-47,951,175
TRB09RV5-4_P	47,948,085-47,948,023	47,947,836-47,947,615
TRB09RV4-2_F	47,947,356-47,947,294	47,947,108-47,946,818
TRB09RV5-5_F	47,946,487-47,946,425	47,946,307-47,946,008
TRB09RV5-6_P	47,944,264-47,944,214	47,944,096-47,943,797
TRB09RV4-3_F	47,943,532-47,943,470	47,943,281-47,942,991
TRB09RV8-1_F	47,930,266-47,930,199	47,930,028-47,929,738
TRB09RV6-1_P	No match	47,926,422-47,926,127
TRB09RV14-1	47,925,199-47,925,128	47,925,050-47,924,763
TRB09RV7-1_F	47,919,547-47,919,501	47,919,280-47,918,987
TRB09RD		47,738,970-47,738,923
TRB09RJ1		47,738,469-47,738,377
TRB09RJ2		47,738,274-47,738,197

TRB09RJ3		47,738,094-47,737,981
TRB09RJ4		47,737,876-47,737,772
TRB09RJ5		47,737,355-47,737,257
TRB09RJ6		47,737,099-47,737,028
TRB09RJ7		47,736,699-47,736,616
TRB09RJ8		47,735,321-47,735,039
TRB09RJ9		47,734,774-47,734,694
TRB09RJ10		47,734,452-47,734,348
TRB09RC		47,732,468-47,731,039

(FWD) indicates 5'-3' transcription of gene.

1.2 Data on deduced constant domain amino acid sequences from selected species

Alignment of selected deduced amino acid sequences for TRB Constant domain. Dots indicate identities and dashes are missing residues. Numbering according to SasaTRB01C sequence. Transmembrane domain is underlined.

```
                       *        20         *        40         *        60
SasaTRB01C  : EPDIPVTPPKVKVLPPSTKECEDRNKKKKKTLVCVATDFYPDHVTVFWQLNGGANITDGVGT :  62
SasaTRB01RC : .............................................................. :  62
Satr1       : .....................................................V........ :  62
Onmy19      : --............................................................ :  60
Onts1       : --.......T......................................K............. :  60
SasaTRB09C  : D.N.K..E.T.E..A..A...K...............R...........V.N-V.R.E.A.. :  61
SasaTRB09RC : D.N.K..E.T.E..A..A...K...............R...........V.N-V.R.E.A.. :  61
Satr9       : D.N.K..E.T...A..A...K...N...........R...........V.N-V.R.E.A.. :  61
Onmy25      : D.N.K..E.T...A..A...................R...........V.N-V.R.E.A.. :  61
Onts9       : D.N.K..E.T...A..A...................R...........V.N-V.R.E.A.. :  61
Onki9       : D.N.K..E.T...A..A..............................K............. :  62
IcpuTCRB1   : D..MKLQA.T.T..NV.E..VCT---.ENV.....KG.....K.Y.TVDE-V.R.ID.S. :  58
IcpuTCRB2   : D.NMEIQA.N.T..NV.E..VCT---.ENV.....KG.....K.Y.KVDE-V.R.TD.S. :  58
hsTCRBC1-2  : D-LNK.F..E.A.FE..EA.ISH---TQ.A....L..G.F....ELS.WV..-KEVHS..S. :  58
hsTCRBC2-2  : D-LKN.F....A.FE..EA.ISH---TQ.A....L..G......ELS.WV..-KEVHS..S. :  58

                       *        80         *       100         *
SasaTRB01C  : DNTALRD----GNRRYSITSRLRVPAKKWNKASNRFTCTVRFFNGNDDIYVADH-------- : 112
SasaTRB01RC : .......----...........................................-------- : 112
Satr1       : .......----...........................................-------- : 112
Onmy19      : .......----E...............T..T..............T..V.....-------- : 110
Onts1       : .......----E...............T..T..............T........-------- : 110
SasaTRB09C  : ..K..W.----KDSL............D.HNPD.K...I.S.Y...G..N.N.T-------- : 111
SasaTRB09RC : ..K..W.----KDSL............D.HNPD.K...I.S.Y...G..N.N.T-------- : 111
Satr9       : ..K..W.----KDSL............D.QNPD.....I.S.Y...GY.N.KHT-------- : 111
Onmy25      : ..R..W.----KDGL...........NE.H.PE.....I.S.YD.T.N.R.N.T-------- : 111
Onts9       : ..R..W.----KDGL...........NE.H.PE.....N.S.Y..T.N.Q.N.T-------- : 111
IcpuTCRB1   : .EA.VQG----SDKY.T.S...NIDY.TEWTRGKT...I.N...-GTG.NYK.S-------- : 107
IcpuTCRB2   : .EA.IQG----SDKY.T.S...NIDY.TEWTRGKT...I.N...NMT..DYQ.S-------- : 108
hsTCRBC1-2  : .PQP.KEQPALNDS..CLS.....S.TF.QNPR.H.R.Q.Q.YGLSENDEWTQDRAKPVTQI : 120
hsTCRBC2-2  : .PQP.KEQPALNDS..CLS.....S.TF.QNPR.H.R.Q.Q.YGLSENDEWTQDRAKPVTQI : 120

                                              Transmembrane domain Cytoplasmic region
                  Intron 1   Intron 2                            Intron 3
                      120        *         140        *         160
SasaTRB01C  : INGEE GQGGDGEMTT EDYVMSTKTAKLAYSIFIAK-STFYGLVVMALIWRL-QVSS-LQL- : 169
SasaTRB01RC : ..... .......... ....................-...............-....-...- : 169
Satr1       : .....-..........-....................-..V............-....-...- : 169
Onmy19      : M....-.--A..G...-.Y..K..Q............-.............KF-.R..DK.M- : 166
Onts1       : .D...-.--...G...-.Y..K..Q.......F....-.............KF-.R..DK.M- : 166
SasaTRB09C  : .S.DL QGQSG..I.. DY..K..Q............-.........VM..KF .G..EK.I- : 169
SasaTRB09RC : .S.DL QGQSG..I.. DY..K..Q............-.........VM..KF .G..EK.I- : 169
Satr9       : .S.DL-QGQSG..I..-DY..K..R............-.........VM..KF-.G..EK.I- : 169
Onmy25      : .S.DL-QGQSG..I..-DY..K..Q............-.........VM..KF-.G..EK.I- : 169
Onts9       : .R.DL-QGQSG..I..-DY..K..Q............-.........VM..KF-.G..EK.I- : 169
IcpuTCRB1   : .T.PK-LTIDEDNYE.---..R.V..TM.R.GM.V..-.IA..IFI.YIVR.Q-GFM.K---- : 160
IcpuTCRB2   : .T.PK-LTIDEDNYE.---..R.V..TM.R.GM.V..-.IA..IFI.YIVR.Q-GFM.K---- : 161
hsTCRBC1-2  : VSA.A-WGRA.CGF.S-VS.QQGVLS.TIL.E.LLG.-A.L.AVL.S..VLMA-M.KRKDF-- : 177
hsTCRBC2-2  : VSA.A-WGRA.CGF.S-.S.QQGVLS.TIL.E.LLG.-A.L.AVL.S..VLMA-M.KRKDSRG : 179
```

Percent sequence identity for selected TCR beta constant domain amino acid sequences:

	1	2	3	4	5	6	7	8	9	10	11	12	13	14
1: IcpuTCRB1	100	93	43	43	43	41	41	40	40	41	39	41	26	26
2: IcpuTCRB2	93	100	42	42	40	40	40	40	40	41	39	41	25	25
3: SasaTRB09C	43	42	100	100	95	92	91	67	67	67	69	70	34	34
4: SasaTRB09RC	43	42	100	100	95	92	91	67	67	67	69	70	34	34
5: Satr9	43	40	95	95	100	90	89	67	67	67	68	68	35	34
6: Onmy25	41	40	92	92	90	100	98	68	68	68	70	71	33	33
7: Onts9	41	40	91	91	89	98	100	68	68	68	71	72	33	33
8: SasaTRB01C	40	40	67	67	67	68	68	100	100	99	90	90	34	36
9: SasaTRB01RC	40	40	67	67	67	68	68	100	100	99	90	90	34	36
10: Satr1	41	41	67	67	67	68	68	99	99	100	89	88	34	36
11: Onmy19	39	39	69	69	68	70	71	90	90	89	100	96	35	37
12: Onts1	41	41	70	70	68	71	72	90	90	88	96	100	34	35
13: hsTCRBC1-201	26	25	34	34	35	33	33	34	34	34	35	34	100	97
14: hsTCRBC2-201	26	25	34	34	34	33	33	36	36	36	37	35	97	100

TRBC sequence references and genomic locations are as follows: *Salmo trutta* (Satr1) TSA: GFIS01070448.1 chr.3 NC_042989.1:41.909.413 and 41.146.671 and (Satr9) TSA: GFIS01070449.1 chr.25 NC_042981.1:3.820.220 and 3.360.394; *Oncorhynchus mykiss* (Onmy1) BX869299.3 chr.19 NC_048583: 63.760.837-63.764.495 and (Onmy9) TSA: GBTD01144039.1 chr.25 NC_048589.1: 43.177.117 and 43.979.157; *Oncorhynchus tshawytscha* (Onts1) TSA: GGDU01175447.1 LG11 NC_056439.1:53.196.803 and 53.760.293 (Onts9) TSA: GGDU01676935.1 LG08 NC_056436.1:4.215.890 and 5.493.023; IcpuTCRB1 (*Ictalurus punctatus*) U39193 AAA99773.1 and icpuTCRB2 AAB02655.1 TCRB2 (Wilson et al.1998); Human (hs) TCRBC1-201 ENST00000633705.1 Chr.7: 142.791.694-142.793.368 and hsTCRBC2-201 ENST00000466254.1 chr.7:142.801.041-142.802.748.

Data on orthology of Atlantic salmon duplicate TRB01 and TRB09 regions originate from the reference «Lien S, Koop BF, Sandve SR, Miller JR, Kent MP, Nome T, et al. The Atlantic Salmon Genome Provides Insights into Rediploidization. *Nature* (2016) 533(7602):200-5. Epub 2016/04/19. doi: 10.1038/nature17164.» where their supplementary table 6 shows the homology block to be confined to:

Homeolog block	Chr. X	Start	End	Chr. Y	Start	End
1p-9qa_1	ssa01	0	6 843 616	ssa09	43 662 329	48 507 357

1.3 Alignment of deduced TRB variable domain amino acid sequences from TRB01, TRB09 and scaffold regions

```
                            Framework 1                CDR1     Framework 2
09V1-1_P      : ----GSEVTQIPTILWGLKES-DA-P--MNCSHTKGLSYFQMYWYRQLPG-E-GMKQVV* :  49
09V1-2_F      : GLVEGSEVTQIPTILWRLKES-DA-P--MNCSHTKGSGYYQMYWYRQLPG-E-GMKQVAF :  54
09V1-3_P      : ----GSEVTQIPTILWGPKES-DA-Q--MNCSHTKGSSYFQMY*YRQLPG-E-GMKQVAF :  49
09V1-4_F      : GLVEGSEVTQIPTILWEMKDS-DA-L--MNCSHTKGATYFQMYWYRQLPG-E-GMKQVVY :  54
09RV1-1       : VLVEGSEVTQIPTILWKLKES-DA-P--MNCSHTKGSSYYQMYWYRQLPG-E-GMKQVVY :  54
09RV1-2_P     : ----GSEVTQIPTILWGPKES-DA-P--MNCSHTKGSGYYQMYWYRQLPG-E-GMKQVAF :  50
09RV1-3_F     : GLVEGSEVTQIPTILWGLKDR-DA-P--MNCSHTKGSTYNQMYWYRQLPG-K-GMKQVVY :  54
09RV1-4_P     : ----GSEVTQIPTILWGLKES-DA-P--MNCSHTKGSSYNQMYWYRQLPG-E-GMKQVVY :  50
09V2-1        : GSSLSNQVYQSPASLYKNQGE-LA-K--MECSHSI-SGYDRILWYKQSNY-R-ELVLLGY :  53
09V2-2_F      : GSYLSSQVYQNPASLYKNQGE-SA-K--MECSHSI-SSYNRILWYKQSNF-R-ELVLLGY :  53
09V2-3_F      : GSSLNSQVHQRPAALYKNQGE-SA-K--MECSHSI-SGYNYVLWYKQSNY-R-ELVFLGY :  53
09RV2-1_F     : GSSLSNQIYQSPASLYKNQGE-SA-K--IKCSHSI-SSYNVILWYKQSNY-R-ELVFLGY :  53
09RV2-2       : GSSFSSQVYQSPVALYKNQGK-LA-K--MECSHSI-SSYDRILWYKQSNY-R-ELVFLGY :  53
09RV2-3_F     : GSSLSSQVQQRPAALYKNQGE-SA-K--IVCSHSI-SGYNVILWYKQSNY-R-ELVLLGY :  53
09RV2-4       : GSSLSNQVQQRPAALYNNQGE-SD-K--MKCSHSI-SGYNVILWYKQSNY-R-ELVLLGY :  53
09RV2-5_F     : GSSLSSQVYQTPASLYKKQGK-SA-K--MECSHSI-SSYNRILWYKQSNY-R-ELLLLGY :  53
09RV2-6       : GSYLSSQVYQSPASLYKNQGE-LA-K--MECSHSI-SGYNQILWYKQFNY-R-ELVFLGY :  53
01RV3-1       : GLSQTDKVQQTPTAILKGPED-NV-Q--LNCSHTI-PTYNRILWYQQSAQNT-ALKLIGY :  54
01RV3-2_F     : GLSQTDKVQQTPTAILKGPED-NV-Q--LNCSHTI-PSYNTILWYQQSAQNT-ALKLIGY :  54
01RV3-3_F     : GLSQTDKVQQTPTAILKGPED-NV-Q--LNCSHTI-PNYNTILWYQQSAQNT-ALKLIGY :  54
```

```
01RV3-4      : GLSQTDKVQQTPTAILKGPED-NV-Q--LNCSHTI-PSYNIILWYQQSAQNT-ALKLIGY :  54
01RV3-5      : GLSQTDKVQQTPTAILKGPED-NV-Q--LNCSHTI-PSYNIILWYQQSAQNT-ALKLIGY :  54
01RV3-6      : GLSQTDQVQQTPTAILKGPED-NV-Q--LNCSHTI-PSYNTILWYQQSAQNT-ALKLIGY :  54
01RV3-7      : GLSQTDQVQQTPTAILKGPED-NV-Q--LNCSHTI-PSYNTILWYQQSAQNT-ALKLIGY :  54
01RV3-8_F    : GLSQADKVQQTPTAILKGPED-NV-Q--LNCSHTI-PNYNRILWYQQSAQNT-ALKLIGY :  54
01RV3-9      : GLSQTDKVQQTPTAILKGPED-NV-Q--LNCSHTI-PSYNIILWYQQSAQNT-ALKLIGY :  54
01RV3-10     : GLSQTDKVQQTPTAILKGPED-NV-Q--LNCSHTI-PSYNIILWYQQSAQNT-ALKLIGY :  54
01RV3-11     : GLSQTDKVQQTPTAILKGPED-NV-Q--LNCSHTI-PSYNIILWYQQSAQNT-ALKLIGY :  54
01RV3-12     : GLSQTDKVQQTPTAILKGPED-NV-Q--LNCSHTI-PTYNIILWYQQSAQNT-ALKLIGY :  54
01RV3-13     : GLSQTDKVQQTPTAILKGPED-NV-Q--LNCSHTI-PNYDTILWYQQSAQNT-ALKLIGY :  54
01RV3-14     : GLSQTDKVQQTPTAILKGPED-NV-Q--LNCSHTI-PNYDTILWYQQSAQNT-ALKLIGY :  54
01RV3-15     : GLSQIDKVQQTPTAILKGPED-NV-Q--LNCSHTI-PNYDTILWYQQSAQNT-ALKLIGY :  54
01RV3-16     : GLSQIDKVQQTPTAILKGPED-NV-Q--LNCSHTI-PNYDTILWYQQSAQNT-ALKLIGY :  54
01RV3-17     : GLSQIDKVQQTPTAILKGPED-NV-Q--LNCSHTI-PNYDTILWYQQSAQNT-ALKLIGY :  54
01RV3-18     : GLSQIDKVQQTPTAILKGPED-NV-Q--LNCSHTI-PNYDTILWYQQSAQNT-ALKLIGY :  54
01RV3-19     : GLSQIDKVQQTPTAILKGPED-NV-Q--LNCSHTI-PNYDTILWYQQSAQNT-ALKLIGY :  54
01RV3-20_F   : GLSQIDKVQQTPTAILKGPED-NV-Q--LNCSHTI-PSYNIILWYQQSAQNT-ALKLIGY :  54
01RV3-21     : GLSQIDKVQQTPTAILKGPED-NV-Q--LNCSHTI-PNYDTILWYQQSAQNT-ALKLIGY :  54
01RV3-22_F   : GLSQTDKVQQTPTAILNGPED-NV-Q--LNCSHTI-PTYNRILWYQQSAQNT-ALKLIGY :  54
09V3-1_P_F   : GLSQTDKVHQTPTAILKRPED-KV*Q--LTCSHTV-SRYDMILWYQQSAQNT-ALKLIGY :  54
09V3-2_P     : GLSQTDKVHQTPTAILKGPED-KV-Q--LTCKHTV-SSYNMILWYQQSAQNT*TLKLIGY :  54
09V3-3_F     : GLSQTDKVHQTPTAILKRPED-KV-Q--LTCSHTD-SRYDMILWYQLSAQNT-ALKLIGY :  54
09V3-4_F     : GLSPTDKVHQTPTAILKRPEN-KV-Q--LTCRHTV-SSYDMILWYQQAAQNS-ALKLIGY :  54
09RV3-1_P    : GLSQTDKVHQTPTAILKGPHL-QT-HC-FKLQHDT-VVPTVS*YCCTNSQPK-TLKLIGY :  54
09RV3-2_F    : GLSQTDKVHQTPTAILKRPED-KV-H--LTCSHTV-SSYNMILWYQQSPQNN-ALKLIGY :  54
09RV3-3_F    : GLSQTDKVHQTPTAILKGPED-KV-Q--LTCSHTD-TSYYMILWYQQSAQNT-ALKLIGY :  54
09RV3-4_F    : GLSQTDKVHQTPTAILKRPED-KV-N--LTCSHTV-SSYYMILWYQQSAQNT-ALKLIGY :  54
09RV3-5_F    : GLSQTDKVHQTPTAILKGPED-KV-Q--LTCSHTD-PRYDMILWYQQSAQNT-ALKLIGY :  54
09RV3-6_F    : GLSQTDKVHQTPTAILKGPED-KV-Q--LTCSHTV-SRYDMILWYQQSAQNT-ALKLIGY :  54
SCF.RV3S1    : GLSQTDKVQQTPTAILKGPED-NV-Q--LNCSHTI-PNYDTILWYQQSAQNT-ALKLIGY :  54
SCF.RV3S2    : GLSQTDKVQQTPTAILKGPED-NV-Q--LNCSHTI-PSYNIILWYQQSAQNT-ALKLIGY :  54
SCF.RV3S3    : GLSQIDKVQQTPTAILKGPED-NV-Q--LNCSHTI-PNYDTILWYQQSAQNT-ALKLIGY :  54
SCF.RV3S4    : GLSQIDKVQQTPTAILKGPED-NV-Q--LNCSHTI-PNYDTILWYQQSAQNT-ALKLIGY :  54
SCF.RV3S5    : GLSQTDKVQQTPTAILKGPED-NV-Q--LNCSHTI-PSYNIILWYQQSAQNT-ALKLIGY :  54
SCF.RV3S6    : GLSQIDKVQQTPTAILKGPED-NV-Q--LNCSHTI-PNYDTILWYQQSAQNT-ALKLIGY :  54
SCF.RV3S7    : GLSQIDKVQQTPTAILKGPED-NV-Q--LNCSHTI-PNYDTILWYQQSAQNT-ALKLIGY :  54
01V4-1       : GVSLSKVVYQSPSTLLVEPNA-SV-SVPLSCSHKI-PSYNTILWYQRPVGDT-ALKLIAY :  56
01V4-2_P     : GVSLSKVVYQSPSTLLVEPNA-SV-SVPLSCSHKI-PSYNTILWYQRPVGDT-ALKLIAY :  56
01RV4-1      : GVSLSKVVYQSPSTLLVKPNA-PV-SVPLSCSHKI-PSYNTILWYQRPVGDT-ALKLIAY :  56
09RV4-1_F    : CLSFSKSVYQSPSTLLVKPNA-SV-T--LNCSHKI-PSYNTILWSQRPVEDT-ALNLIAY :  54
09RV4-2_F    : GVSLSKSVYQSPSTLLVKPNA-SV-T--LNCSHKI-PSYDTILWYQRSVEDT-ALNLIAY :  54
09RV4-3_F    : GVSLSKSVYQSPSTLLVKPNA-SV-T--LNCSHKI-PSYDTILWYQRSVEDT-ALNLIAY :  54
01V5-1       : GLSLSVEVHQTPSEVFSRPGE-NV-Q--LFCHHNK-SDYRVILWYQKLQGET-ALKLIGY :  54
01RV5-1_P    : GLSLSVEVHQTPSEVFSRPGE-NV-Q--LFCHHNK-SDYRVILWYQKLQGET-ALKLIGY :  54
09V5-1_P     : -----------------------------------SENRVML*YQKSQGET-ALKLIGY :  22
09V5-2_F     : GLCLCVEVHQTPSAVFSRPGE-NI-Q--LFCHHQK-TDYTQMLWYQKSQGET-ALKLIGY :  54
09RV5-1_P    : ----SLEVYQTPSEVLLKHGE-NV-Q--IFCHHER-TNYRLMQWFQQSLVEK-QLKLIAY :  50
09RV5-2_F    : GLSLSVEVHQTPSAVFSRPGE-NI-Q--LFCHHQK-TDYRVMLWYQKSQGET-ALKLIGY :  54
09RV5-3_F    : GLCLCVEVHQTPSAVFSRPGE-NI-Q--LFCHHQK-TDYRMMLWYQKSQGET-ALKLIGY :  54
09RV5-4_P    : -----------------------------------SENRVML*YQKSQGEK-ALKLIGY :  22
09RV5-5_F    : GLSLSVEVHQTPSAVFSRPGE-NI-Q--LFCHHQK-TDYRVMLWYQKSQGET-ALKLIGY :  54
09RV5-6_P    : -----VEVHQTPSAVFSRPGE-NI-Q--LFCHHQK-TDYRMML*YQKSQGET-ALKLIGY :  48
09V6-1_P     : ---------------------------------K-LWYHNMLWYQQVRYGG-PVEFLIK :  24
09V7-1_F     : DSCGGVTVKQSPVLSVCREGDVSV-T--LQCYHDD-SSYYYMFWYRQRDN---NMKMLTY :  53
09RV7-1_F    : DFCGGVTVKQSPVLSVCREGDVSV-T--LQCYHDD-SSYYYMFWYRQRDN---NMKMLTY :  53
09RV14-1     : ESTSGVNFIQHPANLLSIEGE-SG-T--LECEHDD-AEFYRMFWYKQNTN-G-NMELVAY :  53
09RV8-1_F    : GHVKCVAFRQSPS-LTVKDGR-EA-E--IHCSHDD-SNLSVMLWYQQRQASP-GMTLIGY :  53
09V9-1_F     : GSCVSLSVHQSPRYLTKRPGT-TL-K--MTCSHHD-KKYDKIYWYRQIHG-Q-KLQLIGF :  53
01RV10-1_P   : ----------*PSALLESPNV-TV-I--LKCSHTI-SSYYTILWYQQSIADT-NLKLIGF :  43
01RV10-2_F   : GSSLGSKVLQVPSALLESPNV-TV-I--LKCSHTI-SSYYTILWYQQSIADT-NLKLIGF :  54
01RV10-3     : GSSLGSKVLQVPSALLESPNV-TV-I--LKCSHTI-SSYYTILWYQQSIADS-NLKLIGF :  54
01RV10-4     : GSSLGSKVLQVPSALLESPNV-TV-I--LKCSHTI-SSYYTILWYQQSIADS-NLKLIGF :  54
01RV10-5     : GSSLGSKVLQVPSALLESPNV-TV-I--LKCSHTI-SSYYTILWYQQSIADS-NLKLIGF :  54
01RV10-6     : GSSLGSKVLQVPSALLESPNV-TV-I--LKCSHTI-SSYYTILWYQQSIADS-NLKLIGF :  54
01RV10-7     : GSSLGSKVLQVPSALLESPNV-TV-I--LKCSHTI-SSYYTILWYQQSIADS-NLKLIGF :  54
01RV10-8     : GSSLGSKVLKVPSALLESPNV-TV-I--LKCSHTI-SSYYTILWYQQSIADS-NLKLIGF :  54
01RV10-9     : GSSLGSKVLQVPSALLESPNV-TV-I--LKCSHTI-SSYYTILWYQQSIADT-NLKLIGF :  54
SCF.RV10S1   : GSSLGSKVLKVPSALLESPNV-TV-I--LKCSHTI-SSYYTILWYQQSIADS-NLKLIGF :  54
```

```
SCF.RV10S2    : GSSLGSKVLQVPSALLESPNV-TV-I--LKCSHTI-SSYYTILWYQQSIADT-NLKLIGF :  54
SCF.RV10S3    : GSSLGSKVLQVPSALLESPNV-TV-I--LKCSHTI-SSYYTILWYQQSIADT-NLKLIGF :  54
01RV11-1_F    : GQSLSSKVHQTPIAILKGSKD-NV-Q--LTCNHTF-QNYNMILWYQQSTGDN-AMNLIGY :  54
01RV11-2      : GQSLSSKVHQTPTAILKGSKD-NV-Q--LTCNHTI-QNYNVILWYQQSTGDN-AMNLIGY :  54
01RV11-3_F    : GQSLSSKVHQTPIAILKGSKD-NV-Q--LTCNHTI-QNYNMILWYQQSTGGT-AMNLIGY :  54
01RV11-4      : GQSLSSKVHQTPTAILKGSKD-NV-Q--LTCNHTI-QGYTMILWYQQSTGDT-AVNLIGY :  54
01RV11-5      : GQSLSSKVHQTPIAVLKGSKD-NV-Q--LTCNHTI-QSYYVILWYQQSTGDT-AVNLIGY :  54
01RV11-6_F    : GQSLSSKVHQTPIAVLKGSKD-NV-Q--LTCNHTI-QNYNVILWYQQSTGDT-AMNLIGY :  54
01RV11-7      : GQSLSSKVHQTPIAVLKGSKD-NV-Q--LTCNHTI-QGYTMILWYQQSTGDT-AMNLIGY :  54
01RV11-8      : GQSLSSKVHQTPIAVLKGSKD-NV-Q--LTCNHTI-QNYIMILWYQQSTGDT-AVNLIGY :  54
01RV11-9      : GQSLSSKVHQTPIAVLKGSKD-NV-Q--LTCNHTI-QGYTMILWYQQSTGDT-AVNLIGY :  54
01RV11-10_F   : GQSLSSKVHQTPTAILKGSKD-NV-Q--LTCNHTI-QNYNVILWYQQSTGDT-AMNLIGY :  54
01RV11-11     : GQSLSSKVHQTPTAILKGSKD-NV-Q--LTCNHTI-QGYTMILWYQQSTGDT-AMNLIGY :  54
01RV11-12     : GQSLSSKVHQTPTAILKGSKD-NV-Q--LTCNHTI-QNYIMILWYQQSTGDT-AVNLIGY :  54
01RV11-13     : GQSLSSKVHQTPTAILKGSKD-NV-Q--LTCNHTI-QNYIMILWYQQSTGDT-AVNLIGY :  54
01RV11-14     : GQSLSSKVHQTPTAILKGSKD-NV-Q--LTCNHTI-QNYIMILWYQQSTGDT-AVNLIGY :  54
01RV11-15_F   : GQSLSTKVHQTPTAILKGSKD-NV-Q--LTCNHTI-QNYNMILWYQQSTGDT-AMNLIGY :  54
09V13-1       : GQLFSSMVHQRPVALTEGPGG-NV-Q--RTCSHTI-PNYYVILWYKQSAGDT-AMKLIGY :  54
09RV13-1      : GQLFSSMVHQRPVALTEGPGG-NF-Q--LTCSHTI-PNYYVILWYKQSAGDT-AMKLIGY :  54
09RV13-2      : GQLFSSMVHQRPVALTEGPGG-NV-Q--LTCSHTI-PNYYMILWYKQSAGDT-AMKLIGY :  54
09RV13-3_F    : GQLFSSMVHQRPVALTEGPGG-NV-P--LTCSHTI-PNYYMILWYKQSAGDT-AMKLIGY :  54
SCF.RV11S1    : GQSLSSKVHQTPTAILKGSKD-NV-Q--LTCNHTI-QGYTMILWYQQSTGDT-AVNLIGY :  54
SCF.RV11S2_P  : GQSLSSKVHQTPIAVLKGSKD-NV-Q--LTCNHTI-QGYTMILWYQQSTGDT-AMNLIGY :  54
SCF.RV11S3    : GQSLSSKVHQTPIAVLKGSKD-NV-Q--LTCNHTI-QSYYVILWYQQSTGDT-AVNLIGY :  54
SCF.RV11S4_F  : GQSLSSNVHQTPIAVLKGSKD-NV-Q--LTCNHTI-QNYYVILWYQQSTGDT-AVNLIGY :  54
SCF.RV11S5_P  : GQSLSSKVHQTPTAILKGSKD-NV-Q--LTCNHTI-QG*TMILWYQQSTGDT-AVNLIGY :  53
SCF.RV11S6_P  : GQSLSSKVHQTPIAVLKGSKD-NV-Q--LTCNHTI-QNYNMILWYQQSTGDT-AMNLIGY :  54
SCF.RV11S7    : GQSLSSKVHQTPIAVLKGSKD-NV-Q--LTCNHTI-QSYYVILWYQQSTGDT-AVNLIGY :  54
SCF.RV11S8_F  : GQSLSSKVHQTPIAVLKGSKD-NV-Q--LTCNHTI-QNYNVILWYQQSTGDT-AMNLIGY :  54
SCF.RV11S9_F  : GQSLSSKVHQTPIAVLKGSKD-NV-Q--LTCNHTI-QNYNMILWYQQSTGDT-AMNLIGY :  54
09V12-1       : GLGLGTRVVQTPAALVTTPGD-SA-D--LRCSHTI-KDYDVILWYRQSHSQDRQLQLLGY :  55

                CDR2                     Framework 3                  CDR3

09V1-1_P      : TSTYNKPDY-GDFSED-KYPIVKAVA-----ESGSFTVKKLE-AGDSGMYFC-AVS :  96
09V1-2_F      : TIPNSKPEYSGDFSKD-KFSAKKTVA-----ESGSFTVKKLE-TGDSGMYFC-AVS : 102
09V1-3_P      : TTSNSKPEYSGDFSED-KFSAKKTVV-----ESGSFTVKKLE-AGDSGMYFC-AVS :  97
09V1-4_F      : TTSSSKPEYSGDFSED-KFSAKKTVV-----ESGSFTVKKLE-AGDSGMYFC-AVS : 102
09RV1-1       : TTSSSKPEYSWDFSED-KFSAKKTVA-----ESGSFTVKKLG-AGDSGMYFC-AVS : 102
09RV1-2_P     : TIPNSKPEYSGDFSKD-KFSAKKTVA-----ESG*FTVKKLE-TGDSGMYFC-AVS :  97
09RV1-3_F     : TSSYSRPDY-GDFSED-KYPIVKAVA-----ESGSFTVKKLE-AGDSGMYFC-AVS : 101
09RV1-4_P     : TSTYNKPDY-*DFSED-KYSIVKAVA-----ESGSFTVKKSE-TGDSGMYFC-AVS :  96
09V2-1        : LYGKT-GYPEA------GFGIEGDANA---GGTSTLTINQLT-PNSSAVYYC-AAS :  97
09V2-2_F      : MIATA-GYPEA------GFGIEGDANA---GGTSTLTINQLT-PNSSAVYYC-AAS :  97
09V2-3_F      : MQLKT-GFPEA------GFAIEGDANA---GGTSTLTINQLT-PNSSAVYYC-AAS :  97
09RV2-1_F     : MTAKT-ANPEV------GFGIEGDASA---GGTSTLTINQLT-PNSSAVYYC-AAS :  97
09RV2-2       : MTAKI-GFPEA------GFDIEGDANA---GGTSTLTINQLT-PNSSAVYYC-AAS :  97
09RV2-3_F     : MIATA-GYPEA------GFDIEGDANA---GGTSTLTINQLT-PNSSAVYYC-AAS :  97
09RV2-4       : MIGTS-GYPEA------GFDIEGDANA---GGTSTLTINQLT-PNSSAVYYC-AAS :  97
09RV2-5_F     : MVGKT-GYPEA------GFGIEGDANA---GGTSTLTINQLT-PNSSAVYYC-AAS :  97
09RV2-6       : MTGTS-GFPEA------GFDIEGDANT---GGTSTLTINQLT-PNSSAVYYC-AAS :  97
01RV3-1       : VFLTS-PTVEDSFKE--HFTVSGDAAT---GKMAYLHIPKLRGTEDSAVYFC-AAT : 103
01RV3-2_F     : VFLTS-PAVEDSFKE--RFTVSGDAAT---GKMAYLHIPKLRGTEDSAVYFC-AAT : 103
01RV3-3_F     : VFLTS-PAVEDSFKE--RFTVSGDAAT---GKMAYLHIPKLRGTEDSGVYFC-AAS : 103
01RV3-4       : VLLTS-PTVEDSFKE--RFTVSGDAAT---GKMAYLHIPKLRGTEDSAVYFC-AAT : 103
01RV3-5       : VLLTS-PTVEDSFKE--RFTVSGDAAT---GKMAYLHIPKLRGTEDSAVYFC-AAT : 103
01RV3-6       : VLLTS-PAVEDSFKE--RFTVSGDAAT---GKMAYLHIPKLRGTEDSAVYFC-AAT : 103
01RV3-7       : VLLTS-PAVEDSFKE--RFTVSGDAAT---GKMAYLHIPKLRGTEDSAVYFC-AAT : 103
01RV3-8_F     : VLLTS-PAVEDSFKE--RFTVSGDAAT---GKMAYLHIPKLRGTEDSAVYFC-AAT : 103
01RV3-9       : VFLTS-PTVEDSFKE--RFTVSGDAAT---GKMAYLHIPKLRGTEDSAVYFC-AAT : 103
01RV3-10      : VFLTS-PTVEDSFKE--RFTVSGDAAT---GKMAYLHIPKLRGTEDSAVYFC-AAT : 103
01RV3-11      : VFLTS-PTVEDSFKE--RFTVSGDAAT---GKMAYLHIPKLRGTEDSAVYFC-AAT : 103
01RV3-12      : VFLTS-PTVEDSFKE--RFTVSGDAAT---GKMAYLHIPNLRGTEDSAVYFC-AAT : 103
01RV3-13      : VLLTS-PTMEDSFKE--RFTVSGDAAT---GKMAYLHIPNLRGTEDSAVYFC-AAS : 103
01RV3-14      : VLLTS-PTMEDSFKE--RFTVSGDAAT---GKMAYLHIPNLRGTEDSAVYFC-AAS : 103
01RV3-15      : VRFTS-PTVEDSFKE--RFTVSGDAAT---GKMAYLHVPKLRGTEDSAVYFC-AAS : 103
01RV3-16      : VRFTS-PTVEDSFKE--RFTVSGDAAT---GKMAYLHIPKLRGTEDFAVYFC-AAS : 103
```

01RV3-17	:	VRFTS-PTVEDSFKE--RFTVSGDAAT---GKMAYLHVPKLRGTEDSAVYFC-AAS	:	103
01RV3-18	:	VRFTS-PTVEDSFKE--RFTVSGDAAT---GKMAYLHVPKLRGTEDSAVYFC-AAS	:	103
01RV3-19	:	VRFTS-PTVEDSFKE--RFTVSGDAAT---GKMAYLHIPKLRGTEDSAVYFC-AAT	:	103
01RV3-20_F	:	VRFTS-PTVEDSFKE--RFTVSGDAAT---GKMAYLHIPKLRGTEDSAVYFC-AAS	:	103
01RV3-21	:	VRFTS-PTVEDSFKE--RFTVSGDAAT---GKMAYLHIPKLRGTEDSAVYFC-AAT	:	103
01RV3-22_F	:	VQFTS-PTVEDSFKE--RFTVSGDAAT---GKMAYLHIPKLRGTEDSAVYFC-AAT	:	103
09V3-1_PF	:	VRFTS-PTVEDSFKG--RFNVSGDAAA---NKMAYLHFPKLTEAEDSAVYFC-AAS	:	103
09V3-2_P	:	VSNTI-PTVEDSFKG--RFNVSGDGAA---NKMAYLHFPKVTEAEDSAVYFC-ADS	:	103
09V3-3_F	:	VWNTS-PTVEDSFKG--RFNVSGEGAA---NKMAYLHFPKVTEAEDSAVYFC-AAS	:	103
09V3-4_F	:	VWNTS-PTVEDSFKG--RFNVSGDGAA---NKMAYLHFPKVTEAEDSAVYFC-AAS	:	103
09RV3-1_P	:	VWNTS-PTVEDSFKG--RFNVSGDGAA---NKMAYLHFPKVTEAEDSSVYFC-AAS	:	103
09RV3-2_F	:	VWNTS-PTVEDSFKG--RFNVSGDAAA---NKMAYLHFPKLTEAEDSAVYFC-AAS	:	103
09RV3-3_F	:	VRFTS-PTVEDSFKG--RFNVSGDGAA---NKMAYLHFPKLTEPEDSAVYFC-AAS	:	103
09RV3-4_F	:	VRYTS-PMVEDSFKG--RFNVSGDGAA---NKMAYLHFPKLTEAEDSSVYFC-AAS	:	103
09RV3-5_F	:	VRFTS-PTVEDSFKG--RFNVSGDGAA---SKMAYLHCPKLTEAEDSAVYIS-VLL	:	103
09RV3-6_F	:	VRFTS-PTVEDSFKG--RFNVSGDGAA---NKMAYLHCPKLTEAEDSSVYFC-AAS	:	103
SCF.RV3S1	:	VLLTS-PTMEDSFKE--RFTVSGDAAT---GKMAYLHIPNLRGTEDSAVYFC-AAS	:	103
SCF.RV3S2	:	VQFTS-PTVEDSFKE--RFTVSGDAAT---GKMAYLHIPNLRGTEDSAVYFC-AAS	:	103
SCF.RV3S3	:	VRFTS-PTVEDSFKE--RFTVSGDAAT---GKMAYLHVPKLRGTEDSAVYFC-AAS	:	103
SCF.RV3S4	:	VRFTS-PTVEDSFKE--RFTVSGDAAT---GKMAYLHIPKLRGTEDSAVYFC-AAS	:	103
SCF.RV3S5	:	VQFTS-PTVEDSFKE--RFTVSGDAAT---GKMAYLHIPNLRGTEDSAVYFC-AAS	:	103
SCF.RV3S6	:	VRFTS-PTVEDSFKE--RFTVSGDAAT---GKMAYLHVPKLRGTEDSAVYFC-AAS	:	103
SCF.RV3S7	:	VRFTS-PTVEDSFKE--RFTVSGDAAT---GKMAYLHVPKLRGTEDSAVYFC-AAS	:	103
01V4-1	:	VYITS-QTVEPSYKG--YFDVKGDGR-----NEAFLHLLKLRQAEDSGEYFC-AAS	:	103
01V4-2_P	:	VYITS-QTVEPSYKG--YFDVKGDGR-----NEAF-HLPPSLLP*-----------	:	91
01RV4-1	:	VYYTS-QTVEPSYKG--YFDVKGDGR-----NEAFLHLLKLRQAEDSGEYFC-AAS	:	103
09RV4-1_F	:	AYYKT-PTVEPSYKV--YFDVKGDGE-----NEAFLHILKLRQAEDSGEYFC-AAS	:	101
09RV4-2_F	:	AYYKT-PTVEPSYKG--YFDVKGDGE-----NEAFLHILKLRQAEDSGEYFC-AAS	:	101
09RV4-3_F	:	AYYKT-PTVEPSYKG--YFDVKGDGE-----NKAFLHILKLRQAEDSGEYFC-AAS	:	101
01V5-1	:	LYYQN-PTIESSYGK--HFNMSGDLGG-DRPKNVSLNIFNFQHPEHSAMYFC-AAS	:	105
01RV5-1_P	:	LYYQN-PTIESSYGK--HFNMSGDLGG-DRPKNVSLNIFNIPNTP-----LCISVL	:	101
09V5-1_P	:	LYHKN-PTIESSYEE--HFNMSGDLGG-AGPKNVSLNMFNLRQTQHSAVYFC-AAS	:	73
09V5-2_F	:	LNYNS-KTIESSYEN--NFNMSGDLSG-DGPKNASLNMFNLRQPQHSAVYLC-AAR	:	105
09RV5-1_P	:	LYFKS-QT--TYIEE--HLNVSGDLSQ-NDPKNVSLNMFNLKGPDHSAV*FC-AAS	:	98
09RV5-2_F	:	LNFNS-QTIESSYEK--HFNMSGDLGG-DGPKNASLNMFNLRQTQNSAVYFC-AAS	:	105
09RV5-3_F	:	LNYNS-PTIESSYEK--HFKMSGDLGG-NGPKNASLNMFNLRQPQHSAVYFC-AAS	:	105
09RV5-4_P	:	LNVNS-PTIESSYEE--HFNMSGDLGG-AGPKNASLNMFNLRQPQYSAVYFC-AAS	:	73
09RV5-5_F	:	LNFNS-QIIESSYEK--HFNMSGDLGG-DGPKNASLNMFNLRQTQNSAVYFC-AAS	:	105
09RV5-6_P	:	LNYNS-QTIESSYEE--HFNMSGDLGG-AGPKNASLNMFNLRQPQHSAVYLC-AAS	:	99
09V6-1_P	:	EYEKT----QGRYKE--NLDTSGNRFILQVMESI*LQISELV-VEDSGVYYC-AAS	:	71
09V7-1_F	:	SLGQGVWEIEPPFEKDVHYTMSRPEL-----TRSTLEIKNLA-VGDGAVYYC-ASS	:	102
09RV7-1_F	:	SLGQGVWEIEPPFEKDVHYTMSRPEL-----TRSTLEIKNLE-VGDGAVYYC-ASS	:	102
09RV14-1	:	SMGTRATDIEPIFDKA-NYNMIHDEQ-----LKSSLQIKNVK-SVDSAVYYC-AAS	:	101
09RV8-1_F	:	SYSTTEPNYESLFEE--RFRLKIQGD-----LKGTLVISKLT-LADSAVYFC-AAS	:	100
09V9-1_F	:	LSFKE-AV---DVEE--NFNISGDAE-----DEGFLGSLAVR-IEDTGLYYC-AVS	:	96
01RV10-1_P	:	VFYKN-PTVADQYKE--HFEIRGDGE-----IEASLQL--LSGPENSAVYYC-AAS	:	88
01RV10-2_F	:	VFYKN-PTVADQYKE--HFEIRGDGE-----IEASLQL--LSGPENSAVYYC-AAS	:	99
01RV10-3	:	VFYKN-PTVADQYKE--HFEIRGDGE-----IEASLQL--LSGPENSAVYYC-AAS	:	99
01RV10-4	:	VFYKN-PTVADQYKE--HFEIRGDGE-----IEASLQL--LSGPENSAVYYC-AAS	:	99
01RV10-5	:	VFYKN-PTVADQYKE--HFEIRGDGE-----IEASLQL--LSGPENSAVYYC-AAS	:	99
01RV10-6	:	VFYKN-PTVADQYKE--HFEIRGDGE-----IEASLQL--LSGPENSAVYYC-AAS	:	99
01RV10-7	:	VFYKN-PTVADQYKE--HFEIRGDGE-----IEASLQL--LSGPENSAVYYC-AAS	:	99
01RV10-8	:	VFYKN-PTVEDQFKE--HFEIRGDGE-----IEASLQL--LSGPENSAVYYC-AAS	:	99
01RV10-9	:	MFYKN-PTVEDQFKE--HFEIRGDGE-----IEASLQL--LSGPENSAVYYC-AAS	:	99
SCF.RV10S1	:	VFYKN-PTVEDQFKE--HFEIRGDGE-----IEASLQL--LSGPENSAVYYC-AAS	:	99
SCF.RV10S2	:	MFYKN-PTVEDQFKE--HFEIRGDGE-----IEASLQL--LSGPENSAVYYC-AAS	:	99
SCF.RV10S3	:	MFYKN-PTVEDQFKE--HFEIRGDGE-----IEASLQL--LSGPENSAVYYC-AAS	:	99
01RV11-1_F	:	AYYKS-ITMEKLFEK--HFSVSGDGL-----KEAYLHLLSLRATEDSAVYYC-AAS	:	101
01RV11-2	:	AFTKS-ITMEKSFEK--HFSVSGDGS-----KEAYLHLLSLRAPEDSAVYYC-AAS	:	101
01RV11-3_F	:	AYTTS-ITMEKLFEK--HFNVSGDGL-----KEAYLHLLSLRAPEDSAVYYC-AAS	:	101
01RV11-4	:	AYTTS-ITMEKSFEK--HFSVSGDGS-----KEAYLHLLSLRAPEDSAVYYC-AAS	:	101
01RV11-5	:	AQYKS-ITMEKSFEK--HFSVSGDGS-----KEAYLHLLSLRAPEDSAVYYC-AAS	:	101
01RV11-6_F	:	AYTTS-ITMEKSFEK--HFNVSGDGS-----KEAYLHLLSVRAPEDSAVYYC-AAS	:	101
01RV11-7	:	AYYKS-ITMEKSFEK--HFNVSGDGS-----KEAYLHLLSVRAPEDSAVYYC-AAS	:	101
01RV11-8	:	AQYKS-ITMEKSFEK--HFSVSGDGS-----KEAYLHLLSLRAPEDSAVYYC-AAS	:	101
01RV11-9	:	AYTTS-ITMEKSFEK--HFSVSGDGS-----KEAYLHLLSLRAPEDSAVYYC-AAS	:	101
01RV11-10_F	:	AYYKS-ITMEKSFEK--HFNVSGDGS-----KEAYLHLLSVRAPEDSAVYYC-AAS	:	101
01RV11-11	:	AYYKS-ITMEKSFEK--HFNVSGDGS-----KEAYLHLLSVRAPEDSAVYYC-AAS	:	101

```
01RV11-12     : AQYKS-ITMEKSFEK--HFSVSGDGS-----KEAYLHLLSLRAPEDSAVYYC-AAS : 101
01RV11-13     : AQYKS-ITMEKSFEK--HFSVSGDGS-----KEAYLHLLSLRAPEDSAVYYC-AAS : 101
01RV11-14     : AQYKS-ITMEKSFEK--HFSVSGDGS-----KEAYLHLLSLRAPEDSAVYYC-AAS : 101
01RV11-15_F   : AYTTS-ITMEKSFEK--HFNVSGDGR-----KEAYLHLLSLRAPEDSAVYYC-AAS : 101
09V13-1       : AYTKL-ITMEKLFEK--HFNVSGDSG-----KEAYLHLVSLRGPEHSAVYYC-AAS : 101
09RV13-1      : AYTKS-ITMEKSFEK--HFNVSGDGE-----KEAYLHRVSLRGPEHSAVYYC-AAS : 101
09RV13-2      : AYTKS-ITMEKSFEK--HFNVSGDGG-----KEAYLHRVSLRGPEHSAVYYC-AAS : 101
09RV13-3_F    : AYTKS-ITMEKSFEK--HFNVSGDGE-----KEAYLHLVSLRGPEHSAVYYC-VAS : 101
SCF.RV11S1    : AYTTS-ITMEKSFEK--HFSVSGDGS-----KEAYLHLLSLRAPEDSAVYYC-AAS : 101
SCF.RV11S2_P  : AYYKS-ITMEKSFEK--HFNVSGDGS-----KRLIF------------IY*-----  :  84
SCF.RV11S3    : AQYKS-ITMEKSFEK--HFSVSGDGS-----KEAYLHLLSLRAPEDSAVYYC-AAS : 101
SCF.RV11S4_F  : AQYKS-ITMEKSFEK--HFNVIGDGS-----KEAYLHLLSLRAPEDSAVYYC-AAS : 101
SCF.RV11S5_P  : AYTTS-ITMEKSFEK--HFSVSGDGS-----KEAYLHLLSLRAPEDSAVYYC-AAS : 100
SCF.RV11S6_P  : AYYKS-ITMEKSFEK--HFNVSGDGS-----KRLIF------------IY*-----  :  84
SCF.RV11S7    : AQYKS-ITMEKSFEK--HFSVSGDGS-----KEAYLHLLSLRAPEDSAVYYC-AAS : 101
SCF.RV11S8_F  : AYTTS-ITMEKSFEK--HFNVSGDGS-----KEAYLHLLSVRAPEDSAVYYC-AAS : 101
SCF.RV11S9_F  : AFTKS-ITMEKSFEK--HFNVSGDGS-----KEAYLHLLSLRAPEDSAVYYC-AAS : 101
09V12-1       : LYTDN-KNPESSFKD--KIKLRGNAE-----QYCDLSVSNLT-QEDSAVYFC-AAR : 101
```

TRB is omitted from the sequence names. Residues are colour coded according to physiochemical properties. P defines pseudogene with internal stop codon and F defines sequence with expressed support. Remaining sequences can be defined as open reading frame (O) sequences.

1.4 Alignment of deduced tentative leader sequences from TRB01, TRB09 and scaffold regions

These leader sequences are tentative as they mostly lack expressed support.

```
                      *        20
TRB01RL3-22 : -MIKLLIHVAT-LPLWVT--- : 16 variable subgroup 3
TRB01RL3-21 : -..........-......--- : 16
TRB01RL3-20 : -..........-......--- : 16
TRB01RL3-19 : -..........-......--- : 16
TRB01RL3-18 : -..........-......--- : 16
TRB01RL3-17 : -..........-......--- : 16
TRB01RL3-16 : -..........-......--- : 16
TRB01RL3-15 : -..........-......--- : 16
TRB01RL3-14 : -..........-......--- : 16
TRB01RL3-13 : -..........-......--- : 16
TRB01RL3-12 : -..........-......--- : 16
TRB01RL3-11 : -..........-......--- : 16
TRB01RL3-10 : -..........-......--- : 16
TRB01RL3-9  : -..........-......--- : 16
TRB01RL3-8  : -..........-......--- : 16
TRB01RL3-7  : -..........-......--- : 16
TRB01RL3-6  : -..........-......--- : 16
TRB01RL3-5  : -..........-......--- : 16
TRB01RL3-4  : -..........-......--- : 16
TRB01RL3-3  : -..........-......--- : 16
TRB01RL3-2  : -..........-......--- : 16
TRB01RL3-1  : -..........-......--- : 16
TRB09RL3-6  : -..........-......--- : 16
TRB09RL3-5  : -..........-......--- : 16
TRB09RL3-4  : -..........-......--- : 16
TRB09RL3-3  : -..........-......--- : 16
TRB09RL3-2  : -..........-......--- : 16
TRB09RL3-1  : -..........-......--- : 16
TRB09L3-4   : -..........-......--- : 16
TRB09L3-3   : -..........-......--- : 16
TRB09L3-2   : -..........-......--- : 16
TRB09L3-1   : -..........-......--- : 16
SCF.RL3S1   : -..........-......--- : 16
SCF.RL3S2   : -..........-......--- : 16
SCF.RL3S3   : -..........-......--- : 16
SCF.RL3S4   : -..........-......--- : 16
SCF.RL3S5   : -..........-......--- : 16
SCF.RL3S6   : -..........-......--- : 16
SCF.RL3S7   : -..........-......--- : 16
```

```
TRB09L13-1  : -.FRV...L.A-......--- : 16 variable subgroup 11
TRB09L13-3  : -.FRV...L.A-......--- : 16
TRB09L13-2  : -.FRV...LLA-......--- : 16
SCF.RL11S1  : -..RV...L.A-......--- : 16
SCF.RL11S2  : -..RV...L.A-......--- : 16
SCF.RL11S5  : -..RV...L.A-......--- : 16
SCF.RL11S6  : -..RV...L.A-......--- : 16
SCF.RL11S8  : -..RV...L.A-......--- : 16
SCF.RL11S9  : -..RV...L.A-......--- : 16
TRB01L11-1  : -..RV...L.A-......--- : 16
TRB01L11-2  : -..RV...L.A-......--- : 16
TRB01L11-3  : -..RV...L.A-......--- : 16
TRB01L11-4  : -..RV...L.A-......--- : 16
TRB01L11-6  : -..RV...L.A-......--- : 16
TRB01L11-7  : -..RV...L.A-......--- : 16
TRB01L11-9  : -..RV...L.A-......--- : 16
TRB01L11-1  : -..RV...L.A-......--- : 16
TRB01L11-1  : -..RV...L.A-......--- : 16
TRB01L11-1  : -..RV...L.A-......--- : 16
TRB01L11-1  : -..RV...L.V-.L....--- : 16
TRB01L11-1  : -..RV...L.V-.L....--- : 16
TRB01L11-1  : -..RV...L.V-.L....--- : 16
TRB01L11-8  : -..RV...L.V-.L....--- : 16
TRB01L11-5  : -..RV...L.V-.L....--- : 16
SCF.RL11S7  : -..RV...L.V-.L....--- : 16
SCF.RL11S4  : -..RV...L.V-.L....--- : 16
SCF.RL11S3  : -..RV...L.V-.L....--- : 16
TRB09L2-1   : -..RI..SITMGYTA.AAG-- : 18 variable subgroup 2
TRB09L2-3   : -V.RI..SITMGYTA.AAG-- : 18
TRB09RL2-6  : -..RI..SITMGYTA.AAG-- : 18
TRB09RL2-5  : -..RI..SITMGYTA.AAG-- : 18
TRB09L2-2   : -..RI..SITMGYTA.AAG-- : 18
TRB09RL2-2  : -..RII.SITMGYTA.AAG-- : 18
TRB09RL2-1  : -..RI..SITMGYTA.ALG-- : 18
TRB09RL2-4  : -..RI..SITMGYTA.A---- : 16
TRB09RL2-3  : -..RI..AITMGYTA.AAG-- : 18
TRB09RL4-1  : -..PP..IITV-PLF.I.--- : 16 variable subgroup 4 and 5
TRB09RL4-2  : -..PP..IITV-PLF.I.--- : 16
TRB09RL4-3  : -..PP..IITV-PLF.I.--- : 16
TRB01L4-1   : -.Y.P..IITV-PLF.I.--- : 16
TRB01L4-2   : -.Y.P..IITV-PLF.I.--- : 16
TRB01RL4-1  : -.C...FIFTV-.LF.IA--- : 16
TRB01L5-1   : -.Y.HFFIIIV-.H..L.--- : 16
TRB09RL5-3  : -.Y.P..IYT---LF.L.--- : 14
TRB09RL5-6  : -.Y.P..IYT---LF.L.--- : 14
TRB09L5-2   : -.Y.P..IYT---LF.L.--- : 14
TRB09L5-1   : MFSSR.NMIRII.LC.LS--- : 18
TRB09RL5-2  : MFSSR.NMIRII.LC.LS--- : 18
TRB09RL5-4  : MFSSR.NMIRII.LC.LS--- : 18
TRB09RL5-5  : MFSSR.NMIRII.LC.LS--- : 18
SCF.RL10S1  : -..RCFLTFTI-SLF.L.--- : 16 variable subgroup 10
SCF.RL10S2  : -..RCFLTFTI-SLF.L.--- : 16
SCF.RL10S3  : -..RCFLTFTI-SLF.L.--- : 16
TRB01L10-2  : -..RCFLTFTI-SLF.L.--- : 16
TRB01L10-3  : -..RCFLTFTI-SLF.L.--- : 16
TRB01L10-4  : -..RCFLTFTI-SLF.L.--- : 16
TRB01L10-5  : -..RCFLTFTI-SLF.L.--- : 16
TRB01L10-6  : -..RCFLTFTI-SLF.L.--- : 16
TRB01L10-7  : -..RCFLTFTI-SLF.L.--- : 16
TRB01L10-8  : -..RCFLTFTI-SLF.L.--- : 16
TRB01L10-9  : -..RCFLTFTI-SLF.L.--- : 16
TRB09L9-1   : -..RV.LTATV-SL..L.--- : 16 variable subgroup 9
TRB09RL1-2  : MFTF.FT-.TVS.LCAA---- : 16 variable subgroup 1
TRB09RL1-3  : MFTF.FT-.TVS.LCAA---- : 16
TRB09RL1-1  : MFTF.FT-.TVS.LCAA---- : 16
TRB09L1-4   : MFTF.FT-.TVS.LCAA---- : 16
TRB09L1-3   : MFTF.FT-.TVS.LCAA---- : 16
TRB09L1-1   : MFTF.FT-.TVS.LCAA---- : 16
TRB09L1-2   : MFTFIFT-.TVS.LCAA---- : 16
TRB09L12-1  : MILA----.CLVSLC-L.--- : 13 variable subgroup 12
TRB09L7-1   : -.SVSMKLLCVT.A.LL.ASK : 20 variable subgroup 7
TRB09RL7-1  : -.SVRMKLLCVT.A.LL.ASK : 20
TRB09RL14-1 : -----MKLYCVLVLFLL.T-- : 14 variable subgroup 14
TRB09RL8-1  : -.SPTTYGLGLFFI.FPC--- : 17 variable subgroup 8
```

1.5 Alignment of rainbow trout TRB joining nucleotide sequences against Atlantic salmon TRBJ from TRB01 and TRB09 regions

```
                      *        20         *        40         *
TRB09J5    : ctagtggt-gccagccaggcttacttcggtggaggaaccaggttaactgttctag : 54
TRB09RJ5   : ..g.....-.............................................. : 54
OnmyTRBJ5  : ...a....-.t.g.......................................... : 54
TRB01J5    : ...a.a..-.........................................c.... : 54
TRB01RJ5   : ...a.a..-.........................................c.... : 54
TRB09J10   : -ctc....-.gttatgct..c.....t..a.ag.....t.a.c....a.....g. : 53
TRB09RJ10  : -ctc....-.gttatgct..c.....t..a.a......t.a.c....a.....g. : 53
OnmyTRBJ10 : -ctc....-..ttatgct..c.....t..a.ag.n...t.a.c....a.....g. : 53
OnmyTRBJ1  : ---acac.-.ga..tg....c.....t..gaat...g...aac.c.g....t... : 51
TRB09J1    : ---acac.-.ga..tg....c.....t..gaat.......aac.c......t... : 51
TRB09RJ1   : ---acac.-.ga..tg....c.....t..gaat.......aac.c......t... : 51
TRB01J1    : ---acac.-.ga..tgt.c.c.....t..aaat.....a.a.c.c......t... : 51
TRB01RJ1   : ---acac.-.ga..tgt.c.c.....t..aaat.....a.a.c.c......t... : 51
OnmyTRBJ2  : ---acttcaaa.caa.ca..c.t...t..caat.......aac.c......t... : 52
TRB01J2    : ---acttcaaa.caa.ca..c.t...t..caat.......aac.c......t... : 52
TRB01RJ2   : ---a.ttcaaa.caa.ca..c.t...t..caat.......aac.c......t... : 52
TRB09J2    : ---actcaaa.tcaa.ca..c.t...t..caat.....g.aac.c......t... : 52
TRB09RJ2   : ---actcaaa.tcaa.ca..c.t...t..caat.....g.aac.c......t... : 52
OnmyTRBJ8  : -----aac-a...a......g........caac..c....aac.c..a.....g. : 49
TRB01RJ7   : -----aac-ct..a....c.g......a.ca.c..c....aac.c..a.....g. : 49
TRB09J8    : -----aac-aa..a..c...a.t......ca.t..c....aac.c..a....... : 49
TRB09RJ8   : -----aac-aa..a..c...a.t......ca.t..c....aac.c..a....... : 49
TRB09J6    : ..g.....-.............................................. : 54
TRB09RJ6   : ..g.....-.............................................. : 54
OnmyTRBJ6  : ..g..t..-.t.g........g.................c............... : 54
OnmyTRBJ7  : -----ctcgta.tcgg....a..tn.tn.a.cc..cn...aac.c..c...t... : 50
TRB09J7    : -----ctcgta.tcgg....a..t..t..a.cc..c....aac.c..c...t... : 50
TRB09RJ7   : -----ctcgta.tcgg....a..t..t..a.cc..c....aac.c..c...t... : 50
TRB01J6    : -----ctcgta.tcgg.a..a.....t..a.cc..c....aac.c..c...t... : 50
TRB01RJ6   : -----ctcgta.tcgg.a..a.....t..a.cc..c....aac.c..c...t... : 50
TRB01J7    : -ctc....-.tttat.ct..c.....t..a.ag.....t.a.c....a.....g- : 52
TRB01RJ9   : -ctc....-..ttat.ct..c.....t..a.ag.....t.a.c....a.....g- : 52
OnmyTRBJ4  : ------cc-aa.tatg....c..t..t..aca...c....aa.....a.....c. : 48
TRB01J4    : ------cc-aa.tatg....ac.t..t...tc...c....aa.....a.....c. : 48
TRB01RJ4   : ------cc-aa.tatg....ac.t..t...tc...c....aa.....a.....c. : 48
TRB09J4    : ------cc-aa.tatg....ac.t..t..atc...c....aa.....a.....c. : 48
TRB09RJ4   : ------cc-aa.tatg....ac.t..t..atc...c....aa.....a.....c. : 48
OnmyTRBJ3  : ---acaac-tatgat.ct..a.t...t..a.cg.......aac.g........g. : 51
TRB01J3    : ---acaac-tatgat.ct..g.t...t..a.c...g....aac.g........g. : 51
TRB01RJ3   : ---acaac-tat.at.ct..g.t...t..a.c...g....aac.g........g. : 51
TRB09J3    : ---acaac-tatgat.ct..a.t...t..a.c...g....aac.g.g......g. : 51
TRB09RJ3   : ---acaac-tatgat.ct..a.t...t..a.c...g....aac.g.g......g. : 51
OnmyTRBJ9  : -----.taca.ggata....ag.g..t...cc...tt....ac.c....------ : 44
TRB09J9    : -----.taca.ggata....ag.g..t...cc...t.....ac.c.......... : 50
TRB09RJ9   : -----.taca.ggata....ag.g..t...cc...t.....ac.c.......... : 50
TRB01RJ8   : -----.aacc.ggaga....ag.g..t...ca.........ac.c.......... : 50
```

Genbank accession number for Rainbow trout (Onmy) joining sequences is U97590.

Similar alignment for the deduced Atlantic salmon and rainbow trout TRBJ amino acid sequences is as follows:

```
                      *
TRB09J1    : -TGSEAYFGNGTKLTVL : 16
TRB09RJ1   : -................ : 16
TRB01J1    : -...VP........... : 16
TRB01RJ1   : -...VP........... : 16
omTRBJ1*01 : -..........A..S.. : 16
TRB01J2    : TSNQP.F.......... : 17
omTRBJ2*01 : TSNQP.F.......... : 17
```

```
TRB01RJ2   : ISNQP.F.......... : 17
TRB09J2    : TQTQP.F.......... : 17
TRB09RJ2   : TQTQP.F.......... : 17
TRB01J3    : -NYDP.F..A....... : 16
omTRBJ3*01 : -NYDP.F..A....... : 16
TRB09J3    : -NYDP.F..A....S.. : 16
TRB09RJ3   : -NYDP.F..A....S.. : 16
TRB01RJ3   : -NYNP.F..A....... : 16
TRB09J8    : -NNNP.F..S....... : 16
TRB09RJ8   : -NNNP.F..S....... : 16
omTRBJ8*01 : -NTNQ............ : 16
TRB01J4    : --NY..H..S....... : 15
TRB01RJ4   : --NY..H..S....... : 15
TRB09J4    : --NY..H..S....... : 15
TRB09RJ4   : --NY..H..S....... : 15
omTRBJ4*01 : --NY.....Q....... : 15
TRB01J7    : SGVYP....E....... : 17
TRB01RJ9   : SGAYP....E....... : 17
TRB09J10   : SG.YA....E....... : 17
TRB09RJ10  : SG.YA....E....... : 17
omTRBJ10*0 : SGAYA....EX...... : 17
TRB01J6    : -SY......A....... : 16
TRB01RJ6   : -SY......A....... : 16
TRB09J7    : -SY......A....... : 16
TRB09RJ7   : -SY......A....... : 16
TRB01RJ7   : -NLNQP..SS....... : 16
TRB01J5    : NSA.Q....G..R.... : 17
TRB01RJ5   : NSA.Q....G..R.... : 17
TRB09J5    : SGA.Q....G..R.... : 17
TRB09RJ5   : GGA.Q....G..R.... : 17
TRB09J6    : GGA.Q....G..R.... : 17
TRB09RJ6   : GGA.Q....G..R.... : 17
omTRBJ5*01 : NGVGQ....G..R.... : 17
omTRBJ6*01 : GCVGQ.D..G..R.... : 17
omTRBJ7*01 : -SY....XXA.X..... : 16
TRB01RJ8   : -NPEK.E..Q..R.... : 16
TRB09J9    : -YTDK.E..P..R.... : 16
TRB09RJ9   : -YTDK.E..P..R.... : 16
omTRBJ9*01 : -YTDK.E..P.SR..-- : 14
```

1.6 Alignment of genomic diversity sequences from rainbow trout and A. salmon TRB01 and TRB09 regions

```
TRB01D        tgatgctttaacactgtGGGGACAGGGGGccacggtgatatattcaca
TRB01RD       tgatgctttaacactgtGGGGACAGGGGGccacggtgatatattcaca
TRB09D        tgatgctttcacactgtGGGGACAGGGGGccacggtgatatatttaca
TRB09RD       tgatgctttcacactgtGGGGACAGGGGGccacggtgatatatttaca
OnmyTRBD      ------------------GGGACAGGGGGC------------------
                                 ************
```

Upper case shows the consensus core diversity sequence. Genbank accession number for rainbow trout TRBD sequence is U97590.

1.7 Nano Illumina run statistics for each primer pair

Run 1				After Flash	Collapsed
SAMPLE #	Forward	Reverse	Number of	Combined_pairs	
	V1.F	TRB09.R	70959	5121	801
	V3.F	TRB09.R	71237	69547	30941
	V4.F	TRB09.R	39164	38411	10841
	V5.F	TRB09.R	57424	4148	1235
	V7.F	TRB09.R	25468	23289	6448
	V8.F	TRB09.R	8858	6359	1939
	V9.F	TRB09.R	35225	11779	3834
	V11/13.F	TRB09.R	73273	70897	22071
Undetermined			152831		
Run 2				After Flash	
SAMPLE #	Forward	Reverse	Number of	Combined pairs	Collapsed
	V2.F	TRB09.R	4332	4276	2811
	V3.F	TRB01.R	19932	9753	6907
	V5.F	TRB01.R	2965	57	20
	V10.F1	TRB01.R	3627	81	34
	V11/13.F	TRB01.R	391387	320550	128403
Undetermined			182539		

For collapsed sequences, see SF3.

1.8 Expressed support for genomic TRB01, TRB09 and scaffold variable sequences

Selected translated expressed support for genomic TRB01, TRB09 and scaffold variable sequences. Each collapsed sequence can be found in SF3 sorted per primer combination where numbers refer to sequence number, e.g. sequence number 50 with 16 supporting combined reads (50-16). Joining, diversity and constant domains are not included in the below nomenclature.

Run1. Selected translated expressed support for genomic TRB09 variable sequences

```
>TRB09V1-2 sequence 50-16
GLVEGSEVTQIPTILWRLKESDAPMNCSHTKGSGYYQMYWYRQLPGEGMKQVAFTIPNSK
PEYSGDFSKDKFSAKKTVAESGSFTVKKLETGDSGMYFCAVSEFDRGGTKLTVLDPNIKV
TEPTVEVLAPSAKECKDRNKKKKKTLVCVATRFYPDHVTVFWQV
>TRB09V1-4 sequence 38-18
GLVEGSEVTQIPTILWEMKDSDALMNCSHTKGATYFQMYWYRQLPGEGMKQVVYTTSSSK
PEYSGDFSEDKFSAKKTVVESGSFTVKKLEAGDSGMYFCAVRHIFGSGTKLTVLDPNIKV
TEPTVEVLAPSAKECKDRNKKKKKTLVCVATRFYPDHVTVFWQV
```

>TRB09RV1-3 sequence 47-16
GLVEGSEVTQIPTILWGLKDRDAPMNCSHTKGSTYNQMYWYRQLPGKGMKQVVYTSSYSR
PDYGDFSEDKYPIVKAVAESGSFTVKKLEAGDSGMYFCAVSQFGAGTKLTVLDPNIKVTE
PTVEVLAPSAKECKDRNKKKKKTLVCVATRFYPDHVTVFWQV
>TRB09V3-1_P sequence 93-18
VQLTCSHTVSRYDMILWYQQSAQNTALKLIGYVRFTSPTVEDSFKGRFNVSGDAAANKMA
YLHFPKLTEAEDSAVYFCAASLGQSSGASQAYFGGGTRLTVLDPNIKVTEPTVEVLAPSA
KECKDRNKKKKKTLVCVATRFYPDHVTVFWQV
>TRB09V3-3 sequence 102-17
VQLTCSHTDSRYDMILWYQLSAQNTALKLIGYVWNTSPTVEDSFKGRFNVSGEGAANKMA
YLHFPKVTEAEDSAVYFCAASRTSSYSEAYFGAGTKLTVLDPNIKVTEPTVEVLAPSAKE
CKDRNKKKKKTLVCVATRFYPDHVTVFWQV
>TRB09V3-4 sequence 89-18
VQLTCRHTVSSYDMILWYQQAAQNSALKLIGYVWNTSPTVEDSFKGRFNVSGDGAANKMA
YLHFPKVTEAEDSAVYFCAASKATVYTGSEAYFGNGTKLTVLDPNIKVTEPTVEVLAPSA
KECKDRNKKKKKTLVCVATRFYPDHVTVFWQV
>TRB09RV3-2 sequence 60-22
VQLTCSHTVSSYNMILWYQQSPQNNALKLIGYVWNTSPTVEDSFKGRFNVSGDAAANKMA
YLHFPKLTEAEDSAVYFCAASRDRATDKAEFGPGTRLTVLDPNIKVTEPTVEVLAPSAKE
CKDRNKKKKKTLVCVATRFYPDHVTVFWQV
>TRB09RV3-3 sequence 191-14
VQLTCSHTDTSYYMILWYQQSAQNTALKLIGYVRFTSPTVEDSFKGRFNVSGDGAANKMA
YLHFPKLTEPEDSAVYFCAARGPDKAEFGPGTRLTVLDPNIKVTEPTVEVLAPSAKECKD
RNKKKKKTLVCVATRFYPDHVTVFWQV
>TRB09RV3-4 sequence 231-13
VQLTCSHTVSSYYMILWYQQSAQNTALKLIGYVRYTSPMVEDSFKGRFNVSGDGAANKMA
YLHFPKLTEAEDSSVYFCAASDRGNNPAFFGSGTKLTVLDPNIKVTEPTVEVLAPSAKEC
KDRNKKKKKTLVCVATRFYPDHVTVFWQV
>TRB09RV3-5 sequence 20-88
VQLTCSHTDPRYDMILWYQQSAQNTALKLIGYVRFTSPTVEDSFKGRFNVSGDGAASKMA
YLHCPKLTEAEDSAVYFCAARDSSYSEAYFGAGTKLTVLDPNIKVTEPTVEVLAPSAKEC
KDRNKKKKKTLVCVATRFYPDHVTVFWQV
>TRB09RV3-6 sequence 66-20
VQLTCSHTVSRYDMILWYQQSAQNTALKLIGYVRFTSPTVEDSFKGRFNVSGDGAANKMA
YLHCPKLTEAEDSSVYFCAASNQGDNYEAHFGSGTKLTVLDPNIKVTEPTVEVLAPSAKE
CKDRNKKKKKTLVCVATRFYPDHVTVFWQV
>TRB09RV4-1 sequence 160-35
PNASVTLNCSHKIPSYNTILWSQRPVEDTALNLIAYAYYKTPTVEPSYKVYFDVKGDGEN
EAFLHILKLRQAEDSGEYFCAAKDRGRRQASGASQAYFGGGTRLTVLDPNIKVTEPTVEV
LAPSAKECKDRNKKKKKTLVCVATRFYPDHVTVFWQV
>TRB09RV4-2 sequence 14-59
PNASVTLNCSHKIPSYDTILWYQRSVEDTALNLIAYAYYKTPTVEPSYKGYFDVKGDGEN
EAFLHILKLRQAEDSGEYFCAASLGTGGSYEAHFGSGTKLTVLDPNIKVTEPTVEVLAPS
AKECKDRNKKKKKTLVCVATRFYPDHVTVFWQV
>TRB09RV4-3 sequence 10-74
PNASVTLNCSHKIPSYDTILWYQRSVEDTALNLIAYAYYKTPTVEPSYKGYFDVKGDGEN
KAFLHILKLRQAEDSGEYFCAAVDREYSEAYFGAGTKLTVLDPNIKVTEPTVEVLAPSAK
ECKDRNKKKKKTLVCVATRFYPDHVTVFWQV
>TRB09V5-2 sequence 26-32
VHQTPSAVFSRPGENIQLFCHHQKTDYTQMLWYQKSQGETALKLIGYLNYNSKTIESSYE
NNFNMSGDLSGDGPKNASLNMFNLRQPQHSAVYLCAARGYEAHFGSGTKLTVLDPNIKVT
EPTVEVLAPSAKECKDRNKKKKKTLVCVATRFYPDHVTVFWQV
>TRB09RV5-2 sequence 11-37
VHQTPSAVFSRPGENIQLFCHHQKTDYRVMLWYQKSQGETALKLIGYLNFNSQTIESSYE
KHFNMSGDLGGDGPKNASLNMFNLRQTQNSAVYFCAASKHPAFFGAGTKLSVLDPNIKVT
EPTVEVLAPSAKECKDRNKKKKKTLVCVATRFYPDHVTVFWQV
>TRB09RV5-3 sequence 8-38
VHQTPSAVFSRPGENIQLFCHHQKTDYRMMLWYQKSQGETALKLIGYLNYNSPTIESSYE
KHFKMSGDLGGNGPKNASLNMFNLRQPQHSAVYFCAASTHPAFFGSGTKLTVLDPNIKVT
EPTVEVLAPSAKECKDRNKKKKKTLVCVATRFYPDHVTVFWQV
>TRB09RV5-5 sequence 6-41
VHQTPSAVFSRPGENIQLFCHHQKTDYRVMLWYQKSQGETALKLIGYLNFNSQIIESSYE
KHFNMSGDLGGDGPKNASLNMFNLRQTQNSAVYFCAAKDPAFFGAGTKLSVLDPNIKVTE
PTVEVLAPSAKECKDRNKKKKKTLVCVATRFYPDHVTVFWQV
>TRB09V7-1 sequence 52-43
SPVLSVCREGDVSVTLQCYHDDSSYYYMFWYRQRDNNMKMLTYSLGQGVWEIEPPFEKDV
HYTMSRPELTRSTLEIKNLAVGDGAVYYCASSTTAYEAHFGSGTKLTVLDPNIKVTEPTV
EVLAPSAKECKDRNKKKKKTLVCVATRFYPDHVTVFWQV
>TRB09RV7-1 sequence 30-47
SPVLSVCREGDVSVTLQCYHDDSSYYYMFWYRQRDNNMKMLTYSLGQGVWEIEPPFEKDV
HYTMSRPELTRSTLEIKNLEVGDGAVYYCASDGTQTQPAFFGNGTKLTVLDPNIKVTEPT
VEVLAPSAKECKDRNKKKKKTLVCVATRFYPDHVTVFWQV
>TRB09RV8-1 sequence 51-34
VAFRQSPSLTVKDGREAEIHCSHDDSNLSVMLWYQQRQASPGMTLIGYSYSTTEPNYESL
FEERFRLKIQGDLKGTLVISKLTLADSAVYFCAASRQGRYSEAYFGAGTKLTVLDPNIKV
TEPTVEVLAPSAKECKDRNKKKKKTLVCVATRFYPDHVTVFWQV
>TRB09V9-1 sequence 2-101

SCVSLSVHQSPRYLTKRPGTTLKMTCSHHDKKYDKIYWYRQIHGQKLQLIGFLSFKEAVD
VEENFNISGDAEDEGFLGSLAVRIEDTGLYYCAVIQGPDKAEFGPGTRLTVLDPNIKVTE
PTVEVLAPSAKECKDRNKKKKKTLVCVATRFYPDHVTVFWQV
>TRB09RV13-3 sequence 5-329
QLTCSHTIPNYYMILWYKQSAGDTAMKLIGYAYTKSITMEKSFEKHFNVSGDGEKEAYLH
LVSLRGPEHSAVYYCVASQGQGGYAAYFGEGTKLTVLDPNIKVTEPTVEVLAPSAKECKD
RNKKKKKTLVCVATRFYPDHVTVFWQV

Run2. Selected translated expressed support for genomic TRB01, TRB09 and scaffold variable sequences

Chr.09 sequences:

>TRB09V2-2 sequence 22-6
QGESAKMECSHSISSYNRILWYKQSNFRELVLLGYMIATAGYPEAGFGIEGDANAGGTST
LTINQLTPNSSAVYYCAASLGGTQPAFFGNGTKLTVLDPNIKVTEPTVEVLAPSAKECKD
RNKKKKKTLVCVATRFYPDHVTVFWQV
>TRB09V2-3 sequence 35-5
QGESAKMECSHSISGYNYVLWYKQSNYRELVFLGYMQLKTGFPEAGFAIEGDANAGGTST
LTINQLTPNSSAVYYCAALDSGYAAYFGEGTKLTVLDPNIKVTEPTVEVLAPSAKECKDR
NKKKKKTLVCVATRFYPDHVTVFWQV
>TRB09RV2-1 sequence 18-6
QGESAKIKCSHSISSYNVILWYKQSNYRELVFLGYMTAKTANPEVGFGIEGDASAGGTST
LTINQLTPNSSAVYYCAGQSSYSEAYFGAGTKLTVLDPNIKVTEPTVEVLAPSAKECKDR
NKKKKKTLVCVATRFYPDHVTVFWQV
>TRB09RV2-3 sequence 15-6
QGESAKIVCSHSISGYNVILWYKQSNYRELVLLGYMIATAGYPEAGFDIEGDANAGGTST
LTINQLTPNSSAVYYCAASPAASQTQPAFFGNGTKLTVLDPNIKVTEPTVEVLAPSAKEC
KDRNKKKKKTLVCVATRFYPDHVTVFWQV
>TRB09RV2-5 sequence 7-7
QGESAKMECSHSISSYNRILWYKQSNYRELLLLGYMVGKTGYPEAGFGIEGDANAGGTST
LTINQLTPNSSAVYYCAGRQGNNPAFFGSGTKLTVLDPNIKVTEPTVEVLAPSAKECKDR
NKKKKKTLVCVATRFYPDHVTVFWQV

Expressed deduced translated TRB09 sequences used in Fig.4 supporting usage of all joining sequences:

>2-101
SCVSLSVHQSPRYLTKRPGTTLKMTCSHHDKKYDKIYWYRQIHGQKLQLIGFLSFKEAVD
VEENFNISGDAEDEGFLGSLAVRIEDTGLYYCAVIQGPDKAEFGPGTRLTVLDPNIKVTE
PTVEVLAPSAKECKDRNKKKKKTLVCVATRFYPDHVTVFWQV
>4-58
SCVSLSVHQSPRYLTKRPGTTLKMTCSHHDKKYDKIYWYRQIHGQKLQLIGFLSFKEAVD
VEENFNISGDAEDEGFLGSLAVRIEDTGLYYCAVRTGANYEAHFGSGTKLTVLDPNIKVT
EPTVEVLAPSAKECKDRNKKKKKTLVCVATRFYPDHVTVFWQV
>5-53
SCVSLSVHQSPRYLTKRPGTTLKMTCSHHDKKYDKIYWYRQIHGQKLQLIGFLSFKEAVD
VEENFNISGDAEDEGFLGSLAVRIEDTGLYYCAVRTGYSEAYFGAGTKLTVLDPNIKVTE
PTVEVLAPSAKECKDRNKKKKKTLVCVATRFYPDHVTVFWQV
>6-52
SCVSLSVHQSPRYLTKRPGTTLKMTCSHHDKKYDKIYWYRQIHGQKLQLIGFLSFKEAVD
VEENFNISGDAEDEGFLGSLAVRIEDTGLYYCAVSKQYEAHFGSGTKLTVLDPNIKVTEP
TVEVLAPSAKECKDRNKKKKKTLVCVATRFYPDHVTVFWQV
>7-49
SCVSLSVHQSPRYLTKRPGTTLKMTCSHHDKKYDKIYWYRQIHGQKLQLIGFLSFKEAVD
VEENFNISGDAEDEGFLGSLAVRIEDTGLYYCAVSRTANYEAHFGSGTKLTVLDPNIKVT
EPTVEVLAPSAKECKDRNKKKKKTLVCVATRFYPDHVTVFWQV
>8-47
SCVSLSVHQSPRYLTKRPGTTLKMTCSHHDKKYDKIYWYRQIHGQKLQLIGFLSFKEAVD
VEENFNISGDAEDEGFLGSLAVRIEDTGLYYCAVSNRIYSEAYFGAGTKLTVLDPNIKVT
EPTVEVLAPSAKECKDRNKKKKKTLVCVATRFYPDHVTVFWQV
>11-39
SCVSLSVHQSPRYLTKRPGTTLKMTCSHHDKKYDKIYWYRQIHGQKLQLIGFLSFKEAVD
VEENFNISGDAEDEGFLGSLAVRIEDTGLYYCAVSKDYDPAFFGAGTKLSVLDPNIKVTE
PTVEVLAPSAKECKDRNKKKKKTLVCVATRFYPDHVTVFWQV
>12-37
SCVSLSVHQSPRYLTKRPGTTLKMTCSHHDKKYDKIYWYRQIHGQKLQLIGFLSFKEAVD
VEENFNISGDAEDEGFLGSLAVRIEDTGLYYCAVSWDNYEAHFGSGTKLTVLDPNIKVTE
PTVEVLAPSAKECKDRNKKKKKTLVCVATRFYPDHVTVFWQV
>13-37
SCVSLSVHQSPRYLTKRPGTTLKMTCSHHDKKYDKIYWYRQIHGQKLQLIGFLSFKEAVD
VEENFNISGDAEDEGFLGSLAVRIEDTGLYYCAVRDNYEAHFGSGTKLTVLDPNIKVTEP
TVEVLAPSAKECKDRNKKKKKTLVCVATRFYPDHVTVFWQV
>14-36
SCVSLSVHQSPRYLTKRPGTTLKMTCSHHDKKYDKIYWYRQIHGQKLQLIGFLSFKEAVD
VEENFNISGDAEDEGFLGSLAVRIEDTGLYYCAVSKGQTDKAEFGPGTRLTVLDPNIKVT

EPTVEVLAPSAKECKDRNKKKKKTLVCVATRFYPDHVTVFWQV
>15-36
SCVSLSVHQSPRYLTKRPGTTLKMTCSHHDKKYDKIYWYRQIHGQKLQLIGFLSFKEAVD
VEENFNISGDAEDEGFLGSLAVRIEDTGLYYCAVSRDRDSEAYFGAGTKLTVLDPNIKVT
EPTVEVLAPSAKECKDRNKKKKKTLVCVATRFYPDHVTVFWQV
>16-35
SCVSLSVHQSPRYLTKRPGTTLKMTCSHHDKKYDKIYWYRQIHGQKLQLIGFLSFKEAVD
VEENFNISGDAEDEGFLGSLAVRIEDTGLYYCAVSKGRYDPAFFGAGTKLSVLDPNIKVT
EPTVEVLAPSAKECKDRNKKKKKTLVCVATRFYPDHVTVFWQV
>17-35
SCVSLSVHQSPRYLTKRPGTTLKMTCSHHDKKYDKIYWYRQIHGQKLQLIGFLSFKEAVD
VEENFNISGDAEDEGFLGSLAVRIEDTGLYYCAVSQGGEAYFGNGTKLTVLDPNIKVTEP
TVEVLAPSAKECKDRNKKKKKTLVCVATRFYPDHVTVFWQV
>19-34
SCVSLSVHQSPRYLTKRPGTTLKMTCSHHDKKYDKIYWYRQIHGQKLQLIGFLSFKEAVD
VEENFNISGDAEDEGFLGSLAVRIEDTGLYYCARAHFGSGTKLTVLDPNIKVTEPTVEVL
APSAKECKDRNKKKKKTLVCVATRFYPDHVTVFWQV
>20-34
SCVSLSVHQSPRYLTKRPGTTLKMTCSHHDKKYDKIYWYRQIHGQKLQLIGFLSFKEAVD
VEENFNISGDAEDEGFLGSLAVRIEDTGLYYCAVSYRGGYAAYFGEGTKLTVLDPNIKVT
EPTVEVLAPSAKECKDRNKKKKKTLVCVATRFYPDHVTVFWQV
>21-34
SCVSLSVHQSPRYLTKRPGTTLKMTCSHHDKKYDKIYWYRQIHGQKLQLIGFLSFKEAVD
VEENFNISGDAEDEGFLGSLAVRIEDTGLYYCAVSKDSNPAFFGSGTKLTVLDPNIKVTE
PTVEVLAPSAKECKDRNKKKKKTLVCVATRFYPDHVTVFWQV
>22-33
SCVSLSVHQSPRYLTKRPGTTLKMTCSHHDKKYDKIYWYRQIHGQKLQLIGFLSFKEAVD
VEENFNISGDAEDEGFLGSLAVRIEDTGLYYCAVRDSYEAHFGSGTKLTVLDPNIKVTEP
TVEVLAPSAKECKDRNKKKKKTLVCVATRFYPDHVTVFWQV
>112-27
SCVSLSVHQSPRYLTKRPGTTLKMTCSHHDKKYDKIYWYRQIHGQKLQLIGFLSFKEAVD
VEENFNISGDAEDEGFLGSLAVRIEDTGLYYCAVRQGGASQAYFGGGTRLTVLDPNIKVT
EPTVEVLAPSAKECKDRNKKKKKTLVCVATRFYPDHVTVFWQV

Chr.01 sequences:

>TRB01RV3-2 sequence 29-8
VQLNCSHTIPSYNTILWYQQSAQNTALKLIGYVFLTSPAVEDSFKERFTVSGDAATGKMA
YLHIPKLRGTEDSAVYFCAATRTANYEAHFGSGTKLTVLEPDIPVTPPKVKVLPPSTKEC
EDRNKKKKKTLVCVATDFYPDHVTVFWQLNGGANITDGVGTDN
>TRB01RV3-3 sequence 49-7
VQLNCSHTIPNYNTILWYQQSAQNTALKLIGYVFLTSPAVEDSFKERFTVSGDAATGKMA
YLHIPKLRGTEDSGVYFCAASRGRDPAFFGAGTKLTVLEPDIPVTPPKVKVLPPSTKECE
DRNKKKKKTLVCVATDFYPDHVTVFWQLNGGANITDGVGTDN
>TRB01RV3-8 sequence 50-7
VQLTCSHTIPNYNRILWYQQSAQNTALKLIGYVLLTSPAVEDSFKERFTVSGDAATGKMA
YLHIPKLRGTEDSAVYFCAATTGDLNQPYFSSGTKLTVLEPDIPVTPPKVKVLPPSTKEC
EDRNKKKKKTLVCVATDFYPDHVTVFWQLNGGANITDGVGTDN
>TRB01RV3-20 sequence 44-7
VQLNCSHTIPSYNIILWYQQSAQNTALKLIGYVRFTSPTVEDSFKERFTVSGDAATGKMA
YLHIPKLRGTEDSAVYFCAANNYNPAFFGAGTKLTVLEPDIPVTPPKVKVLPPSTKECED
RNKKKKKTLVCVATDFYPDHVTVFWQLNGGANITDGVGTDN
>TRB01RV3-22 sequence 1661-35
QLTCSHTIPTYNRILWYQQSAQNTALKLIGYVQFTSPTVEDSFKERFTVSGDAATGKMAY
LHIPKLRGTEDSAVYFCAATGTSNYEAHFGSGTKLTVLEPDIPVTPPKVKVLPPSTKECE
DRNKKKKKTLVCVATDFYPDHVTVFWQLNGGANITDGVGTDN
>TRB01RV10-2 sequence 8-1
QVPSALLESPNVTVILKCSHTISSYYTILWYQQSIADTNLKLIGFVFYKNPTVADQYKEH
FEIRGDGEIEASLQLLSGPENSAVYYCAAKGDRNYEAHFGSGTKLTVLEPDIPVTPPK
>TRB01RV11-1 sequence 63-98
QLTCSHTFQNYNMILWYQQSTGDNAMNLIGYAYYKSITMEKLFEKHFSVSGDGLKEAYLH
LLSLRATEDSAVYYCAASQKGEAHFGSGTKLTVLEPDIPVTPPKVKVLPPSTKECEDRNK
KKKKTLVCVATDFYPDHVTVFWQLNGGANITDGVGTDN
>TRB01RV11-3 sequence 124-75
QLTCSHTIQNYNMILWYQQSTGGTAMNLIGYAYTTSITMEKLFEKHFNVSGDGLKEAYLH
LLSLRAPEDSAVYYCAASQGSYSEAYFGAGTKLTVLEPDIPVTPPKVKVLPPSTKECEDR
NKKKKKTLVCVATDFYPDHVTVFWQLNGGANITDGVGTDN
>TRB01RV11-6 sequence 15-212
QLTCSHTIQNYNVILWYQQSTGDTAMNLIGYAYTTSITMEKSFEKHFNVSGDGSKEAYLH
LLSVRAPEDSAVYYCAASQSSYSEAYFGAGTKLTVLEPDIPVTPPKVKVLPPSTKECEDR
NKKKKKTLVCVATDFYPDHVTVFWQLNGGANITDGVGTDN
>TRB01RV11-10 sequence 72-93
QLTCSHTIQNYNVILWYQQSTGDTAMNLIGYAYYKSITMEKSFEKHFNVSGDGSKEAYLH
LLSVRAPEDSAVYYCAASRDTGSVPYFGNGTKLTVLEPDIPVTPPKVKVLPPSTKECEDR
NKKKKKTLVCVATDFYPDHVTVFWQLNGGANITDGVGTDN
>TRB01RV11-15 sequence 2-380
QLTCSHTIQNYNMILWYQQSTGDTAMNLIGYAYTTSITMEKSFEKHFNVSGDGRKEAYLH

```
LLSLRAPEDSAVYYCAASNTGSVPYFGNGTKLTVLEPDIPVTPPKVKVLPPSTKECEDRN
KKKKKTLVCVATDFYPDHVTVFWQLNGGANITDGVGTDN
>Scf.RV11S4 sequence 41-125
QLTCSHTIQNYYVILWYQQSTGDTAVNLIGYAQYKSITMEKSFEKHFNVIGDGSKEAYLH
LLSLRAPEDSAVYYCAASRTDYNYNPAFFGAGTKLTVLEPDIPVTPPKVKVLPPSTKECE
DRNKKKKKTLVCVATDFYPDHVTVFWQLNGGANITDGVGTDN
>Scf.RV11S8 sequence 27-149
QLTCSHTIQNYNVILWYQQSTGDTAMNLIGYAYTTSITMEKSFEKHFNVSGDGSKEAYLH
LLSVRAPEDSAVYYCAASPTGGYEAHFGSGTKLTVLEPDIPVTPPKVKVLPPSTKECEDR
NKKKKKTLVCVATDFYPDHVTVFWQLNGGANITDGVGTDN
>Scf.RV11S9 sequence 52-106
QLTCSHTIQNYNMILWYQQSTGDTAMNLIGYAFTKSITMEKSFEKHFNVSGDGSKEAYLH
LLSLRAPEDSAVYYCAASQGGNQPSFFGNGTKLTVLEPDIPVTPPKVKVLPPSTKECEDR
NKKKKKTLVCVATDFYPDHVTVFWQLNEGANITDGVGTDN
```

Selected Translated Expressed sequences that have 100% sequence identity to several genomic sequences:

```
>23-8 matches TRB01RV3-9, TRB01RV3-10, TRB01RV3-11
VQLTCSHTIPSYNIILWYQQSAQNTALKLIGYVFLTSPTVEDSFKERFTVSGDAATGKMA
YLHIPKLRGTEDSAVYFCAATTYNYDPAFFGAGTKLTVLEPDIPVTPPKVKVLPPSTKEC
EDRNKKKKKTLVCVATDFYPDHVTVFWQLNGGANITDGVGTDN
>32-8 matches TRB01RV3-14, TRB01RV3-17, TRB01RV3-18, Scf.RV3S3, Scf.RV3S6, Scf.RV3.7
VQLNCSHTIPNYDTILWYQQSAQNTALKLIGYVRFTSPTVEDSFKERFTVSGDAATGKMA
YLHVPKLRGTEDSAVYFCAASTYTGSVPYFGNGTKLTVLEPDIPVTPPKVKVLPPSTKEC
EDRNKKKKKTLVCVATDFYPDHVTVFWQLNGGANITDGVGTDN
>30-8 matches TRB01RV3-16, TRB01RV3-19, TRB01RV3-21, Scf.RV3S4
VQLNCSHTIPNYDTILWYQQSAQNTALKLIGYVRFTSPTVEDSFKERFTVSGDAATGKMA
YLHIPKLRGTEDSAVYFCAATGETGSVPYFGNGTKLTVLEPDIPVTPPKVKVLPPSTKEC
EDRNKKKKKTLVCVATDFYPDHVTVFWQLNGGANITDGVGTDN
>9-11 matches TRBRV3-13, TRB01RV3-14, Scf.RV3S1
VQLNCSHTIPNYDTILWYQQSAQNTALKLIGYVLLTSPTMEDSFKERFTVSGDAATGKMA
YLHIPNLRGTEDSAVYFCAASTGGGSVPYFGNGTKLTVLEPDIPVTPPKVKVLPPSTKEC
EDRNKKKKKTLVCVATDFYPDHVTVFWQLNGGANITDGVGTDN
>31-8 matches Scf.RV3S2, Scf.RV3S5
VQLNCSHTIPSYNIILWYQQSAQNTALKLIGYVQFTSPTVEDSFKERFTVSGDAATGKMA
YLHIPNLRGTEDSAVYFCAASKQINYEAHFGSGTKLTVLEPDIPVTPPKVKVLPPSTKEC
EDRNKKKKKTLVCVATDFYPDHVTVFWQLNGGANITDGVGTDN
>11-256 matches TRB01RV11-4, TRB01RV11-9, Scf.RV11S1
QLTCSHTIQGYTMILWYQQSTGDTAVNLIGYAYTTSITMEKSFEKHFSVSGDGSKEAYLH
LLSLRAPEDSAVYYCAARDTGSVPYFGNGTKLTVLEPDIPVTPPKVKVLPPSTKECEDRN
KKKKKTLVCVATDFYPDHVTVFWQLNGGANITDGVGTDN
>51-106 matches TRB01RV11-5, Scf.RV11S3, Scf.RV11S7
QLTCSHTIQNYIMILWYQQSTGDTAVNLIGYAQYKSITMEKSFEKHFSVSGDGSKEAYLH
LLSLRAPEDSAVYYCAASGQNQPAFFGNGTKLTVLEPDIPVTPPKVKVLPPSTKECEDRN
KKKKKTLVCVATDFYPDHVTVFWQLNGGANITDGVGTDN
>29-146 matches TRB01RV11-7, TRB01RV11-11
QLTCSHTIQGYTMILWYQQSTGDTAMNLIGYAYYKSITMEKSFEKHFNVSGDGSKEAYLH
LLSVRAPEDSAVYYCAASRTGNQPAFFGNGTKLTVLEPDIPVTPPKVKVLPPSTKECEDR
NKKKKKTLVCVATDFYPDHVTVFWQLNGGANITDGVGTDN
>4-311 matches TRB01RV11-8, TRB01RV11-12, TBR01RV11-13, TRB01RV11-14
QLTCSHTIQSYYVILWYQQSTGDTAVNLIGYAQYKSITMEKSFEKHFSVSGDGSKEAYLH
LLSLRAPEDSAVYYCAARQGALNYEAHFGSGTKLTVLEPDIPVTPPKVKVLPPSTKECED
RNKKKKKTLVCVATDFYPDHVTVFWQLNGGANITDGVGTDN
```

1.9 Linking scaffold NW_025550634.1 to chromosome 1

Alignment of unique scaffold V11 sequences using TRB01RJ7 and TRB01RJ8 joining sequences. Names of expressed sequences reflect the collapsed sequence number e.g. 1667 with number of supporting reads 35. Sequences can be found in SF3.

```
                      *        20         *        40         *        60
Scf.RV11S9 : -------VHQTPIAVLKGSKDNVQLTCNHTIQNYNMILWYQQSTGDTAMNLIGYAFTKSITME :  56
1667-35    : -----------------------....S................................. :  40
1827-33    : -----------------------....S................................. :  40
305-53     : -----------------------....S................................. :  40
2417-29    : -----------------------....S................................. :  40
Scf.RV11S4 : GQSLSSN.........................YV...........V......QY...... :  63
2340-29    : -----------------------....S......YV...........V......QY...... :  40
2380-29    : -----------------------....S......YV...........V......QY...... :  40
937-42     : -----------------------....S......YV...........V......QY...... :  40
```

```
                       *          80          *         100          *        120
Scf.RV11S9 : KSFEKHFNVSGDGS--KEAYLHLLSLRAPEDSAVYYCAAS---------------------- :  94
1667-35    : ..............--.......................QGS---LNQPYFSSGTKLTVLEP :  98 1RJ7
1827-33    : ..............--......................GTG----LNQPYFSSGTKLTVLEP :  97
305-53     : ..............--.......................-QG-N-PEKAEFGQGTRLTVLEP :  98 1RJ8
2417-29    : ..............--......................D-KS-N-PEKAEFGQGTRLTVLEP :  98
Scf.RV11S4 : .........I....--.......................---------------------- : 101
2340-29    : .........I....--.......................PGA-SNLNQPYFSSGTKLTVLEP : 100 1RJ7
2380-29    : .........I....--.......................RGD---LNQPYFSSGTKLTVLEP :  98
937-42     : .........I....--.......................RD--SNPEKAEFGQGTRLTVLEP :  99 1RJ8

                                  *        140          *        160          *        180
Conserved domain for all:_DIPVTPPKVKVLPPSTKECEDRNKKKKKTLVCVATDFYPDHVTVFWQLNGGANITDGVGTDN : 160
```

Alignment of Scf.TRBRV11.9 with all regional TRB01 joining sequences

```
                           *        20         *        40         *        60         *        80         *
Scf.TRBRV11S9 : GQSLSSKVHQTPIAVLKGSKDNVQLTCNHTIQNYNMILWYQQSTGDTAMNLIGYAFTKSITMEKSFEKHFNVSGDGSKEAYLHLLSLRAPEDSA :  94

                  100         *       120         *       140         *       160         *       180
Scf.TRBRV11S9: VYYCAAS---------------------------------------------------------------------------------- : 101
1293-38      : .......H--LSGAYPAYFGEGTKLTVLEPDIPVTPPKVKVLPPSTKECEDRNKKKKKTLVCVATDFYPDHVTVFWQLNGGANITDGVGTDN : 161 1RJ9
1897-33      : .......Q--GSGAYPAYFGEGTKLTVLEPDIPVTPPKVKVLPPSTKECEDRNKKKKKTLVCVATDFYPDHVTVFWQLNGGANITDGVGTDN : 161
24-153       : ......RQ---GTPEKAEFGQGTRLTVLEPDIPVTPPKVKVLPPSTKECEDRNKKKKKTLVCVATDFYPDHVTVFWQLNGGANITDGVGTDN : 160 1RJ8
305-53       : .......Q---GNPEKAEFGQGTRLTVLEPDIPVTPPKVKVLPPSTKECEDRNKKKKKTLVCVATDFYPDHVTVFWQLNGGANITDGVGTDN : 160
1667-35      : .......QGS---LNQPYFSSGTKLTVLEPDIPVTPPKVKVLPPSTKECEDRNKKKKKTLVCVATDFYPDHVTVFWQLNGGANITDGVGTDN : 160 1RJ7
112-77       : ......KQ---GPYSEAYFGAGTKLTVLEPDIPVTPPKVKVLPPSTKECEDRNKKKKKTLVCVATDFYPDHVTVFWQLNGGANITDGVGTDN : 160 1RJ6
982-41       : .......Q--AGPYSEAYFGAGTKLTVLEPDIPVTPPKVKVLPPSTKECEDRNKKKKKTLVCVATDFYPDHVTVFWQLNGGANITDGVGTDN : 161
883-42       : .......Q---GKYSEAYFGAGTKLTVLEPDIPVTPPKVKVLPPSTKECEDRNKKKKKTLVCVATDFYPDHVTVFWQLNGGANITDGVGTDN : 160
273-55       : .......Q---GAYSEAYFGAGTKLTVLEPDIPVTPPKVKVLPPSTKECEDRNKKKKKTLVCVATDFYPDHVTVFWQLNGGANITDGVGTDN : 160
1172-39      : .......QG--SSASQAYFGGGTRLTVLEPDIPVTPPKVKVLPPSTKECEDRNKKKKKTLVCVATDFYPDHVTVFWQLNGGANITDGVGTDN : 161 1RJ5
1220-39      : ......RQG--SSASQAYFGGGTRLTVLEPDIPVTPPKVKVLPPSTKECEDRNKKKKKTLVCVATDFYPDHVTVFWQLNGGANITDGVGTDN : 161
1809-34      : .......R---ASASQAYFGGGTRLTVLEPDIPVTPPKVKVLPPSTKECEDRNKKKKKTLVCVATDFYPDHVTVFWQLNGGANITDGVGTDN : 160
3-371        : .....VG----RE--EAHFGSGTKLTVLEPDIPVTPPKVKVLPPSTKECEDRNKKKKKTLVCVATDFYPDHVTVFWQLNGGANITDGVGTDN : 157 1RJ4
130-73       : .......Q-D--TNYEAHFGSGTKLTVLEPDIPVTPPKVKVLPPSTKECEDRNKKKKKTLVCVATDFYPDHVTVFWQLNGGANITDGVGTDN : 160
772-44       : .......Q----ANYEAHFGSGTKLTVLEPDIPVTPPKVKVLPPSTKECEDRNKKKKKTLVCVATDFYPDHVTVFWQLNGGANITDGVGTDN : 159
202-60       : .......Q-FRDSNYEAHFGSGTKLTVLEPDIPVTPPKVKVLPPSTKECEDRNKKKKKTLVCVATDFYPDHVTVFWQLNGGANITDGVGTDN : 162
446-49       : .......R-QGRSYNPAFFGAGTKLTVLEPDIPVTPPKVKVLPPSTKECEDRNKKKKKTLVCVATDFYPDHVTVFWQLNGGANITDGVGTDN : 162 1RJ3
907-42       : ......----GRNYNPAFFGAGTKLTVLEPDIPVTPPKVKVLPPSTKECEDRNKKKKKTLVCVATDFYPDHVTVFWQLNGGANITDGVGTDN : 159
639-45       : .......--QQGSYNPAFFGAGTKLTVLEPDIPVTPPKVKVLPPSTKECEDRNKKKKKTLVCVATDFYPDHVTVFWQLNGGANITDGVGTDN : 161
90-82        : .....VD----REDQPAFFGNGTKLTVLEPDIPVTPPKVKVLPPSTKECEDRNKKKKKTLVCVATDFYPDHVTVFWQLNGGANITDGVGTDN : 159 1RJ2
33-138       : ......RT-GAS-NQPAFFGNGTKLTVLEPDIPVTPPKVKVLPPSTKECEDRNKKKKKTLVCVATDFYPDHVTVFWQLNGGANITDGVGTDN : 161
265-55       : ......RT-G---NQPAFFGNGTKLTVLEPDIPVTPPKVKVLPPSTKECEDRNKKKKKTLVCVATDFYPDHVTVFWQLNGGANITDGVGTDN : 159
148-68       : .......R---DTGSVPYFGNGTKLTVLEPDIPVTPPKVKVLPPSTKECEDRNKKKKKTLVCVATDFYPDHVTVFWQLNGGANITDGVGTDN : 160 1RJ1
50-108       : ......----GQTGSVPYFGNGTKLTVLEPDIPVTPPKVKVLPPSTKECEDRNKKKKKTLVCVATDFYPDHVTVFWQLNGGANITDGVGTDN : 159
143-70       : ......R--GQGLGSVPYFGNGTKLTVLEPDIPVTPPKVKVLPPSTKECEDRNKKKKKTLVCVATDFYPDHVTVFWQLNGGANITDGVGTDN : 161
656-45       : ......---GQGAGSVPYFGNGTKLTVLEPDIPVTPPKVKVLPPSTKECEDRNKKKKKTLVCVATDFYPDHVTVFWQLNGGANITDGVGTDN : 160
```

1.10 Alignment of selected TRB01 and scaffold variable nucleotide sequences

```
                         *        20         *        40         *        60         *        80         *
Scf.RV3S1    : ggactgtcacaaacagacaaagtccagcagactcctactgcaatactaaaaggacctgaagataacgttcaactgaactgcagccatact :  90
TRB01RV3-14  : .......................................................................................... :  90
TRB01RV3-13  : .......................................................................................... :  90
TRB01RV3-22  : ..g...............................................t....................................... :  90
TRB01RV3-21  : ..g..........t............................................................................ :  90
TRB01RV3-18  : ..g..........t............................................................................ :  90
TRB01RV3-17  : ..g..........t............................................................................ :  90
TRB01RV3-15  : ..g..........t............................................................................ :  90
Scf.RV3S6    : ..g..........t............................................................................ :  90
Scf.RV3S3    : ..g..........t............................................................................ :  90
Scf.RV3S7    : ..g..........t............................................................................ :  90
Scf.RV3S4    : ..g..........t............................................................................ :  90
Scf.RV3S5    : ..g....................................................................................... :  90
Scf.RV3S2    : ..g....................................................................................... :  90
Scf.RV10S3   : ...agt..t.tgggcagt.......tc...gt...ct.a..tc..t.gg..a.t..ca.t.t..ca..gat...c..a............ :  90
Scf.RV10S2   : ...agt..t.tgggcagt.......tc...gt...ct.a...c..t.gg..a.t..ca.t.t..ca..gat...c..a............ :  90
TRB1RV10-9   : ...agt..t.tgggcagt.......tc...gt...ct.a...c..t.gg..a.t..ca.t.t..ca..gat...c..a............ :  90
Scf.RV10S1   : ...agt..t.tgggcagt.......tca..gt...ct.a...c..t.gg..a.t..ca.t.t..ca..gat...c..a............ :  90
TRB01RV10-8  : ...agt..t.tgggcagt.......tca..gt...ct.a...c..t.gg..a.t..ca.t.t..ca..gat...c..a............ :  90
TRB01RV11-4  : ..g.a...t.tt.gcag.........t....................t......t..a.......t..a.....c.c.....a....... :  90
Scf.RV11S1   : -.g.a...t.tt.gcag.........t....................t......t..a.......t..a.....c.c.....a....... :  89
Scf.RV11S5_P : ---------------------.....t....................t......t..a.......t..a.....c.c.....a....... :  69
TRB01RV11-9  : ..g.a...t.tt.gcag......t..t..........t....g....t......t..a.......t..a.....c.c.....a....... :  90
TRB01RV11-6  : ..g.a...t.tt.gcag......t..t..........t....g....t......t..a.......t..a.....c.c.....a....... :  90
Scf.RV11S8   : -.g.a...t.tt.gcag......t..t..........t....g....t......t..a.......t..a.....c.c.....a....... :  89
Scf.RV11S9   : ..g.a...t.tt.gcag......t..t..........t....g....t......t..a.......t..a.....c.c.....a....... :  90
TRB01RV11-5  : ..g.a...t.tt.gcag......t..t..........t....g....t......t..a.......t..a.....c.c.....a....... :  90
Scf.RV11S7   : ..g.a...t.tt.gcag......t..t..........t....g....t......t..a.......t..a.....c.c.....a....... :  90
Scf.RV11S3   : ..g.a...t.tt.gcag......t..t..........t....g....t......t..a.......t..a.....c.c.....a....... :  90
TRB01RV11-8  : ..g.a...t.tt.gcag......t..t..........t....g....t......t..a.......t..a.....c.c.....a....... :  90
Scf.RV11S4   : ..g.a...t.tt.gcag...c..t..t..........ta...g....t......t..a.......t........c.c.....a....... :  90
Scf.RV11S6_P : ..g.a...t.tt.gcag......t..t..........t....g....t......t..a.......t..a.....c.c.....a....... :  90
Scf.RV11S2_P : ..g.a...t.tt.gcag......t..t..........t....g....t......t..a.......t..a.....c.c.....a....... :  90
```

```
                       100         *       120         *       140         *       160         *       180
Scf.RV3S1     : attccaaactacgacactatactgtggtatcagcagtcagcacaaaacactgctctgaaactcattgggtatgtgctactcaccagtcca : 180
TRB01RV3-14   : .......................................................................................... : 180
TRB01RV3-13   : .......................................................................................... : 180
TRB01RV3-22   : .......c....a...gg...........c..............................................a.t........... : 180
TRB01RV3-21   : ...........................................................................ag.t........... : 180
TRB01RV3-18   : ...........................................................................ag.t........... : 180
TRB01RV3-17   : ...........................................................................ag.t........... : 180
TRB01RV3-15   : ...........................................................................ag.t........... : 180
Scf.RV3S6     : ...........................................................................ag.t........... : 180
Scf.RV3S3     : ...........................................................................ag.t........... : 180
Scf.RV3S7     : ...........................................................................ag.t........... : 180
Scf.RV3S4     : ...........................................................................ag.t........... : 180
Scf.RV3S5     : .......g....a...t............c..............................................a.t........... : 180
Scf.RV3S2     : .......g....a...t............c..............................................a.t........... : 180
Scf.RV10S3    : ...agttc....t.t......t.......c.........at.gctg.t...aa.........g.....a.t.a..t.ttat.ag.a.... : 180
Scf.RV10S2    : ...agttc....t.t......t.......c.........at.gctg.t...aa.........g.....a.t.a..t.ttat.ag.a.... : 180
TRB01RV10-9   : ...agttc....t.t......t.......c.........at.gctg.t...aa.........g.....a.t.a..t.ttat.ag.a.... : 180
Scf.RV10S1    : ...agttc....t.t......t.......c.........at.gctg.tt..aa.........g.....a.t....t.ttat.ag.a.... : 180
TRB01RV10-8   : ...agttc....t.t......t.......c.........at.gctg.tt..aa.........g.....a.t....t.ttat.ag.a.... : 180
TRB01RV11-4   : ....a.gg....act.tg.....a.....c..a......a..ggtg........g....c.....a..t....catatac...gtcaat. : 180
Scf.RV11S1    : ....a.gg....act.tg.....a.....c..a......a..ggtg........g....c.....a..t....catatac...gtcaat. : 179
Scf.RV11S5_P  : ....a.gg...gact.tg.....a.....c..a......a..ggtg........g....c.....a..t....catatac...gtcaat. : 159
TRB01RV11-9   : ....a.gg....act.tg.....a.....c..a......a..ggtg........g....c.....a..t....catatac...gtcaat. : 180
TRB01RV11-6   : ....a.......a.tgtg.....a.....c..a......a..gg.g........a....c........t....catatac...gtcaat. : 180
ScfRV11S8     : ....a.......a.tgtg.....a.....c..a......a..gg.g........a....c........t....catatac...gtcaat. : 179
ScfRV11S9     : ....a.......a.t.tg.....a.....c..a......a..gg.g........a....c........t....cat.tac..agtcaat. : 180
TRB01RV11-5   : ....a..g....t.tgtg.....a.....c..a......a..ggtg........g....c........t....ca.a.ta..agtcaat. : 180
Scf.RV11S7    : ....a..g....t.tgtg.....a.....c..a......a..ggtg........g....c........t....ca.a.ta..agtcaat. : 180
Scf.RV11S3    : ....a..g....t.tgtg.....a.....c..a......a..ggtg........g....c........t....ca.a.ta..agtcaat. : 180
TRB01RV11-8   : ....a.......att.tg.....a.....c..a......a..ggtg........g....c........t....ca.a.ta..agtcaat. : 180
Scf.RV11S4    : ....a.......t.tgtg.....a.....c..a......a..gg.g........g....t........t....ca.a.ta..agtcaat. : 180
Scf.RV11S6_P  : ....a.......a.t.tg.....a.....c..a......a..gg.g........a....c........t....catatta..agtcaat. : 180
Scf.RV11S2_P  : ....a.gg....act.tg.....a.....c..a......a..gg.g........a....c........t....catatta..agtcaat. : 180
```

1.11 Expressed support for TRB01 and TRB09 from other salmonids

Salmonid expressed match to TRB01 constant domain

```
>BX869299.3 Oncorhynchus mykiss cDNA clone, mRNA sequence
ATTATCTGTTCTCTGTATCTCTGTTAATTTTCCCTAGAGGATGAATGACAGAGAGGTTTGAGGTATTTTT
GGAATAGGAGAGTAGCGATGTGACACTGGAAGTGTGCCCTACTTTGGAAATGGAACAAAGCTCACTGTTT
TAGAGCCAGACATCCCTGTCACTCCACCCAAAGTCAAAGTCCTTCCACCCTCCACTAAGGAGTGTGAAGA
TAGAAACAAGAAGAAGAAGAAGACGTTGGTGTGTGTGGCCACCGACTTCTACCCCGACCACGTCACTGTG
TCCTGGCAGTTAAATGGAGGCGCCAACATCACCGATGGAGTGGGGACCGACAACACTGCCTTGAGGGATG
AAAACAGACGCTACAGTATCACCAGCAGACTGAGAGTCCCAGCCAAGACATGGAACACGGCCTCTAACAG
ATTCACCTGCACCGTCCGCTTCTTCAATGGGACCGATGACGTGTATGTTGCAGATCACATTAACGGAGAA
GAAGGTGCTGATGGAGGGATGACGACAGAGTACTACGTGAAGAGCACCCAGACTGCCAAGCTGGCCTACA
GCATCTTCATCGCTAAGAGTACCTTCTACGGCCTGGTCGTCATGGCTCTGATTTGGAAGTTTCAGCGCTC
CTCAGATAAACAGATGTAATTGATCCCTGACCGAGACCCAGCCACCCGTGGAAGCTGAGCTCGGTAGACT
GGAGGGCGTCATGTGTGGCATAAGGCTCTGGTGAAAAGTAGTGCACTTTNAAGTAGTGCACTTTAAAGTA
GTGCACTTTTAAGTAGTGCACTATAAAGGGAATAGGATGCCATTTG
>GFIN01056176.1 TSA: Oncorhynchus mykiss transcribed RNA sequence
ATCTCTCCTCCACTGAGGAAACATATTCATCTTCTCCATCAAGTCTTCTTCACTATGATCAGAGTTCTGA
TTCACCTGGCAGCTCTACCACTCTGGGTGACAGGGCAGTCTCTTAGCAGCAAAGTCCATCAGACTCCTAT
TGCAATACTAAAAGGATCTAAAAATAATGTCCAACTCACCTGCAACCATACTATTCAAAACTACAATACA
ATACTGTGGTACCAACAGTCAACAGGAGACACTGCTATGAAACTCATTGGTTATGCATTTGCCGAGTCAA
TAACCATGGAGAAATCATTTGAAAAGCACTTCAATGTGAGTGGAAATGGTAGGAAAGAGGCTTATCTTCA
TCTACTGAGTCTGAGATCACCTGAAGACAGTGCAGTGTATTACTGTGCAGCCAGCCAGGACTATGAGGCA
CATTTCGGTTCAGGCACCAAATTAACAGTTCTCGAGCCAGACATCCCTGTCACTCCACCCAAAGTAAAAG
TCCTTCCACCCTCCACTAAGGAGTGTGAAGATAGAAACAAGAAGAAGAAGAAGACGTTGGTGTGTGTGGC
CACCGACTTCTACCCCGACCACGTCACTGTGTTCTGGCAGTTAAATGGAGGCGCCAACATCACCGATGGA
GTGGGGACCGACAACACTGCCTTGAGGGATGAAAACAGACGCTACAGTATCACCAGCAGACTGAGAGTCC
CAGCCAAGACATGGAACACGGCCTCTAACAGATTCACCTGCACCGTCCGCTTCTTCAATGGGACCGATGA
CATATATGTTGCAGATCACATGAACGGAGAAGAAGGTGCTGATGGAGGGATGACGACAGAGTACTACGTG
AAGAGCACCCAGACTGCCAAGCTGGCCTACAGCATCTTCATCGCTAAGAGTACCTTCTACGGCCTGGTCG
TCATGGCTCTGATTTGGAAGTTTCAGCGCTCCTCAGATAAACAGATGTAATTGATCCCTGACCGAGACCA
GGCCACCCGTGGAAGCTGAGCTCGGTGGACTGGAGGGCGTCATGTGTGGCATAAGGCTCTGGTGAAAAGT
AGAGCACTATAAAGTAGTGCACTATAAAGTAGTAGAATAGGATGCCATTTGGGACGAAGAACAAACCCAT
TTCTGTCATGTGATTACAACGTACAGTGTGCTGTCTATTATCTGTCACTTACTATACAATGTGAGGATGT
ATTTTATCGTCCCCAATAAACAGAATCATTATTCATGTATTCACTGAGTGTACAAAACATTAAGAACACC
TTCCTCATCCTCCCCCTTGAATTTGTCGGGGCATGGACTCTACAAGGTGTTGAAAGCGTTCCACAGGGAT
ACTGGCCCATGTTNNNNNNACAAGGTGTCTAAAGCGTTCCACAGGGAT
>GFIS01070448.1 TSA: Salmo trutta transcribed RNA sequence reverse complement
ATGATTTTTGAACTGTCTTTATTTATTAAAAAAAAGTGAATGATAGACTAAAAGCAAGCATTCCTAAAGT
ATTTCTGATGCCAAATGCATAGAACAAGGCAGTAGTGGCTGGTACATTACTGCTCAGTATGTCTGCACTG
TGACAACTATGATCCTGCGTTCTTTGGAGCAGGGACCAAACTGACTGTTCTGGGTAAGAGGAATGTTCAC
CATGGTAGAAGTAATGGGACTCTATGTGTGGTCTAGTAGCATAGTGATGTGATGAGTTAGTTGTGGATTC
AGTACATTAGATTAGTTCATACTAGTGTTTCCTGCCAGTCAATGCTTTTTGATGTTAAGACCGTACACTG
```

TGCCAACTATGAGGCACATTTTGGTTCAGGCACCAAATTAACAGTTCTCGAGCCAGACATCCCTGTCACT
CCACCCAAAGTCAAAGTCCTTCCACCCTCCACTAAGGAGTGTGAAGATAGAAACAAGAAGAAGAAGAAGA
CATTGGTGTGTGTGGCCACCGACTTCTACCCCGACCACGTCACTGTGTTCTGGCAGTTAAATGGAGGCGT
CAACATCACCGATGGAGTTGGGACCGACAACACGGCCTTGAGGGATGGAAACAGACGTTACAGTATCACC
AGCAGACTGAGAGTCCCAGCCAAGAAATGGAACAAGGCCTCTAACAGATTCACCTGTACCGTCCGCTTCT
TCAATGGGAACGATGACATATATGTTGCAGATCACATTAACGGAGAAGAAGGTCAAGGTGGTGATGGAGA
GATGACGACAGAGGACTATGTGATGAGCACCAAGACTGCCAAGCTGGCCTACAGCATCTTCATCGCTAAG
AGTACCGTCTACGGCCTGGTCGTCATGGCTCTGATTTGGAGGCTTCAGGTGAGTTCACTGCAACTATGAT
GAGGGACATCACACAGCCATGTCTCTCTCTTCTTCGATCTATGTCTTGTGCTAGCTAGCCTGGTCCCAGA
TCTGTTTGTGTGCTGTCTTGCCAACTCTTATTGTCAGCATGACAATGACATAATGTAGGAGTTGGCAGGA
AAGCACAAACAGATCTGGGACCAGGCTAGCTTTGTGCTAATAAAGGTTAAAAAATGGCACCATACTGTCT
TTATCCTGTATCTCTGTGTTTCCCCATACAGACACCATACTGTCTTTATCCTGTATCTCTG
>GFIS01033762.1 TSA: Salmo trutta transcribed RNA sequence
TCTGTTAAAGACAAACTATTAGCAAATACAGTACTACTCCCTCTGTTAAAGGCAAACTACAGGAAATACA
GTACTTGCTAGCGAGCATCAGCCTGGTTTTCTGTCTGTGAGTATGTGGTGTGCTCTGGTGCTTATCCTGC
CTACTTTGGAGAGGGAACTAAGCTAACAGTTCTGGAGCCAGACATCCCTGTCACTCCACCCAAAGTCAAA
GTCCTTCCACCCTCCACTAAGGAGTGTGAAGATAGAAACAAGAAGAAGAAGAAGACATTGGTGTGTGTGG
CCACCGACTTCTACCCCGACCACGTCACTGTGTTCTGGCAGTTAAATGGAGGCGTCAACATCACCGATGG
AGTTGGGACCGACAACACGGCCTTGAGGGATGGAAACAGACGTTACAGTATCACCAGCAGACTGAGAGTC
CCAGCCAAGAAATGGAACAAGGCCTCTAACAGATTCACCTGTACCGTCCGCTTCTTCAATGGGAACGATG
ACATATATGTTGCAGATCACATTAACGGAGAAGAAGGTCAAGGTGGTGATGGAGGGATGACGACAGAGGA
CTATGTGATGAGCACCAAGACTGCCAAGCTGGCCTACAGCATCTTCATCGCTAAGAGTACCTTCTACGGC
CTGGTCGTCATGGCTCTGATTTGGAGGCTTCAGCGCTCCTCAGATAAACAGATG
>GFIS01033761.1 TSA: Salmo trutta transcribed RNA sequence
AGATTCTGTTTATTGGGGATGATAAAATACATCCTCACATTGTATAGTGACAGTTAATAGACAGCACACT
GTACATTGTAATCACATGATAGACATGGGTATGTTCTCCGTCCCAAATGGCACCCTATTCCATTTATAGT
GCACTACTTTATAGTGCACTACTTTATAGTGCACTACTTTATAGTGCACTACTTTTCACCAGAGCCTTAT
GCCACACATGACGCCCTCCAGTCCACCGAGCTCAGCTTCCACGGGTGGCCGGGTCTCGGTCAGTGATCAA
TTACATCTGTTTATCTGAGGAGCGCTGAAGCCTCCAAATCAGAGCCATGACGACCAGGCCGTAGACGGTA
CTCTTAGCGATGAAGATGCTGTAGGCCAGCTTGGCAGTCTTGGTGCTCATCACATAGTCCTCTGTCGTCA
TCTCTCCATCACCACCTTGACCTTCTTCTCCGTTAATGTGATCTGCAACATATATGTCATCGTTCCCATT
GAAGAAGCGGACGGTACAGGTGAATCTGTTAGAGGCCTTGTTCCATTTCTTGGCTGGGACTCTCAGTCTG
CTGGTGATACTGTAACGTCTGTTTCCATCCCTCAAGGCCGTGTTGTCGGTCCCAACTCCATCGGTGATGT
TGGCGCCTCCATTTAACTGCCAGAACACAGTGACGTGGTCGGGGTAGAAGTCGGTGGCCACACACACCAA
TGTCTTCTTCTTCTTCTTGTTTCTATCTTCACACTCCTTAGTGGAGGGTGGAAGGACTTTGACTTTGGGT
GGAGTGACAGGGATGTCTGGCTCTAGAACAGTGAGTCTGGTTCCTTGACCAAACTCTGCCTTCTCCGTGT
TCCATAGAGAGATACCATGGTTTACATGACAGGCTCTGTAGTATGACCCGGTTCTGACCCAGTTCTACTA
CTTCCCTTGGTCTGAACTATTAAACGGGTTCTTCCAACTGATT
>GGDU01175447.1 TSA: Oncorhynchus tshawytscha Gill transcribed RNA sequence
TATCTCTGAGTATCTCTGTTCATTTTCCCTAGAGGATGAATGAGAGAGAGAGATCTGAGGTAGTATCTGT
TCTCTGTATCTCTGAGTATCTCTGTTCATTTTCCCTAGAGGATGAATGACAGAGAGATCTGAGGTATTTT
TGGAATAGGAGAGTAACAATGTGACACTGGAAGTGTGCCCTACTTAGGAAATGGAACAAAGCTCACTGTT
TTAGAGCCAGACATCCCTGTCACTCCACCCACAGTCAAAGTCCTTCCACCCTCCACTAAGGAGTGTGAAG
ACAGGAACAAGAAGAAGAAGAAGACGTTGGTGTGTGTGGCCACCGACTTCTACCCCGACCACGTCACTGT
GTTCTGGAAGTTAAATGGAGGCGCCAACATCACCGATGGAGTGGGGACCGACAACACTGCCTTGAGGGAT
GAAAACAGACGCTACAGTATCACCAGCAGACTGAGAGTCCCAGCCAAGACATGGAACACGGCCTCTAACA
GATTCACCTGCACCGTCCGCTTCTTCAATGGGACCGATGACATATATGTTGCAGATCACATTGACGGAGA
AGAAGGTGGTGATGGAGGGATGACGACAGAGTACTACGTGAAGAGCACCCAGACTGCCAAGCTGGCCTAC
AGCTTCTTCATCGCTAAGAGTACCTTCTACGGCCTGGTCGTCATGGCTCTGATTTGGAAGTTTCAGCGAT
CCTCAGATAAACAGATGTAATTGATCCCTGACCGAGACCCGGCCACCTGTGGAAGCTGAGCTCGGTGGAC
TGGAGGGAGTCATGTTTGGCATAAGGCTCTGGTGAAAAGTAGTGCACTATAAAGTAGTGCACTATAAAGG
GAATAGGATGCCAT
>GGDU01175450.1 TSA: Oncorhynchus tshawytscha Gill transcribed RNA sequence
AGTTCATACTAGTGTTACTCCACTCAATGCTTTTTGATGTTAAGCCTGAAACACTGTGCCAACTATGAGG
CACATTTCGGTTCAGGCACCAAATTAACAGTTCTCGAGCCAGACATCCCTGTCACTCCACCCACAGTCAA
AGTCCTTCCACCCTCCACTAAGGAGTGTGAAGACAGGAACAAGAAGAAGAAGAAGACGTTGGTGTGTGTG
GCCACCGACTTCTACCCCGACCACGTCACTGTGTTCTGGAAGTTAAATGGAGGCGCCAACATCACCGATG
GAGTGGGGACCGACAACACTGCCTTGAGGGATGAAAACAGACGCTACAGTATCACCAGCAGACTGAGAGT
CCCAGCCAAGACATGGAACACGGCCTCTAACAGATTCACCTGCACCGTCCGCTTCTTCAATGGGACCGAT
GACATATATGTTGCAGATCACATTGACGGAGAAGAAGGTGGTGATGGAGGGATGACGACAGAGTACTACG
TGAAGAGCACCCAGACTGCCAAGCTGGCCTACAGCTTCTTCATCGCTAAGAGTACCTTCTACGGCCTGGT
CGTCATGGCTCTGATTTGGAAGTTTCAGCGATCCTCAGATAAACAGATGTAATTGATCCCTGACCGAGAC
CCGGCCACCTGTGGAAGCTGAGCTCGGTGGACTGGAGGGAGTCATGTTTGGCATAAGGCTCTGGTGAAAA
GTAGTGCACTATAAAGTAGTGCACTATAAAGGGAATAGGATGCCAT
>GFIT01033054.1 TSA: Salvelinus fontinalis transcribed RNA sequence
GGCGTATCTTCATCTACTGAGTCTGAGAGCACCTGAAGACAGTGCAGTGTATTACTGTGCAGCCGAGGGG
AATATGAGGCACATTTTGGTTCAGGCACCAAATTGACAGTTCTCGAGCCAGGCCTCACTATCTCTCCACC
CAAAGTGAAAGTCCTTCCACCCTCCACTAAGGAGTGTGAAGATAGAAACAAGAAGAAGAAGAAGACCCTG
GTGTGTGTGGCCACCGACTTCTACCCCGACCACGTCACCGTGTTCTGGCAGTTGAATGGAGGCGCCAACA
TCACCGATGGAGTTGGGACCGACAACACGGCCTTGAGTGGTAAAAACAAACTCTACAGTATCACCAGCAG
ACTGAGAGTCCCAGCCAAGAAATGGAACAAGGCCTCTAACAGATTCACCTGCACCGTCCGCTTCTTCAAT
GGGACCTCTGACATATATGTTGCAGATCACATTAACGGAGAAGAAGGTCAAGGTGGTGATGGAGGGATTA
CGACAGAGTACTATGTGAGGAGCACTAAGACTGCCAAGCTGGCCTACAGCATCTTCATCGCTAAGAGTAC
CTTCTACGGCCTGGTCGTCATGGCTCTGATTTGGAAGTTTCAGCGCTCCTCAGATAAACAGATGTAATTG
ATCACTGACCGAGGCCCGGCCACCCGTGGAAGCTGAGCTCGGTGGACTGGACTGCGTCAAGTGTGGCATA
AGGCTCTGGTGAAAAGTAGTGCACTTTAAAGTAGTGCACTATAAAGGGAATAGGGTGCCATCTGGGACGA

```
AGAACAAACCCATGTCTGTCATGTGATTACAATGTACAGTGTGCTTTCTATTAACTGTCACTATACAATG
TGAGGATGTATTTTATCGTCCCCAATAAACAGAATCTTTATTAATGTATTCACTGAGTGGACAAAACATT
AAGAACACCTTCCTCACATTGAGTTGTATCCCCTTTTGCCCTCAGAACAGACTCAATTCGTCAGGTCATG
GACTCTACAAGGTGTCGAAAGCGTTCCACGAGGAATGCTGGCCCATGTTGACTCCAATGCTTC
```

Salmonid expressed match to TRB09 constant domain sequence

```
>U18122.1 Oncorhynchus mykiss T-cell receptor beta chain constant region mRNA, partial cds
GATCCAAACATCAAAGTCACTGAACCCACAGTGAAAGTCCTAGCACCCTCCGCTAAGAAGTGTGAAGACA
GAAACAAGAAGAAGAAGAAGACCCTAGTGTGTGTAGCCACCCGCTTCTACCCCGACCACGTCACCGTCTT
CTGGCAGGTCAACAATGTCAACAGAACTGAAGGTGCCGGGACCGACAACAGGGCCTTGTGGGATAAAGAC
GGTTTATACAGTATCACCAGCAGACTGAGAGTCCCAGCCAATGAATGGCACAAACCAGAGAACAGATTCA
CCTGCATTGTCAGCTTTTACGATGGGACTGACAATATAAGAGTGAATGACACCATTAGTGGAGATCTCCA
AGGTCAAAGTGGGGGAGAGATAACGACAGATTACTATGTGAAGAGCACCCAGACTGCCAAGCTGGCCTAC
AGCATCTTCATCGCTAAGAGTACCTTCTATGGCCTGGTCGTCATGGTTATGATTTGGAAGTTCCAGGGCT
CCTCAGAGAAACAGATTTAACGGAATACAAGCGGGGGAAGGAGGATGTCCTGCATGGGCGTTGATTCTTC
CATGAGATCAAGAACGGACTGGGAAATGGGCAATTCTGGCAAAATGCCAACCGATTCACTTTACGCAGTG
GGCCTGTCTAAATTGGCTTTTTTTTTTTAGCAAAATAATAATAATTTGTCTAGTAAAGAGGGTCTTAAAGA
AAAACATGGGCAGGTGTGTTAGAAATGCCTGGGCCGGTTTCTGGTCCCAGTTTCCCCCTGCATGTAATTG
TATTGTCCATTGTGCTTATTGTTGCCAGTATCTCTTTATTGCCCCGTCTATGTGAATATGTGCTTTGAAG
AATCTCCTATGAACATGAACTGGCAATTTAAATGTATATTTGAATAAATTCAGCCAAACCAATCTTTCCA
TTAAAAAA
>GGDU01676935.1 TSA: Oncorhynchus tshawytscha Stomach transcribed RNA sequence
CCAGGAGACACTCATATCACATCATCATCGTCTTCCCCAGCGTATTCATCATCTTCATCATTATCTTTTT
CTTCATCATGATGTTCAGAGTTCTGATTCACCTGGCAGCTCTACCACTCTGGGTGACAGATCCAAACATT
AAAGTCACTGAACCCACAGTGAAAGTGCTAGCACCCTCCGCTAAGGAGTGTGAAGATAGAAACAAGAAGA
AGAAGAAGACCCTAGTGTGTGTAGCCACCCGCTTCTACCCCGACCACGTCACGGTCTTCTGGCAAGTCAA
CAATGTCAACAGAACTGAAGGTGCCGGGACCGACAACAGGGCCTTGTGGGATAAAGATGGTTTATACAGT
ATCACCAGCAGACTGAGAGTCCCAGCCAATGAATGGCACAAACCAGAGAACAGATTCACCTGCAATGTCA
GCTTTTACAATGGGACTGACAATATACAAGTGAATGACACCATTCGTGGAGATCTCCAAGGTCAAAGTGG
GGGAGAGATAACGACAGATTACTATGTGAAGAGCACCCAGACTGCCAAGCTGGCCTACAGCATCTTCATC
GCTAAGAGTACCTTCTATGGCCTGGTCGTCATGGTTATGATTTGGAAGTTCCAGGGCTCCTCAGAGAAAC
AGATTTAACGGAATACAAGCGGCCCAAGGAGGATGTCCTGCATGGGTGTTGATTCTTCCATGTGCTCAAG
AACGGACTGGCAAATGGGCAATTCTGGCAAATGCCAGATGGACCAATTCATCTTTAGCCCAGTGGGCCTG
TCTAAATGATATTATGTTTTTAGCAAAATAATAATAATTTGTCTAGTAAAGAGGGTCTTAAAGAAAAACA
TGGGCAGGTGTGTTAGAAATGCCGGGGCCGGTTTCTGGTCCCAGTTTGCCCCTGCGTGTAATTGTATTGT
CCATTGTGCTTATTGTTGCCAGTATCTCTTTATTGCCCCGTCTATGTGAAGATGTGCTTTTAAGAATCTC
CTATGAACATGAACTTGCAATTTAAATGTATATTTGAATAAATTCAGCCAAAACAACCTTTCCATTAACT
TACAAAATCAACGTCTGTTTGTTTTTCCAGTAATTCCAGTAGAGTATGTATCAATACACTTTGCTTTCCA
ATTTCTAAATGTTAGTTTGTGAATAATAAACTATATAATTAGGGACAGATCAAAATGTACCTCTCCACAG
TTAAATATTTTCACGACCCTCCCTCCTCATAGAATTAACTCAATATAATGTTTACGACCACAAATAACAA
GAACTGTAGCGACCCTGTTTTTATCAACGTGGATATTGACTTTGCCACTTCAGTCTGCTTTTCTGGCACA
GTAGATGCCACGCAGGGCTTTGGGCCAGAAGGTTGAGGGTTCACCGACCACCACAGACGAGCTCCCTCTC
CCTGCCTGTTTCATCACATTACGATCAGAGACGGAGGCAGACCATGATCAGCTAGGGGGAAATCAGATTT
TCAACATTTTGATGCCATATTTATATAAAATATATTCAACGAATCTTCCACCGACAGGTTTAACAACCAA
TAAACAAAACAAACCAACTTGTGGCACTTCAATATCATGGTTTGTGTTGTGAACAAACAGAAAACACACC
CCTCCCTGTCTTGTAGACTTACCCAGTATTGAAGGCTTGGCTTGACAATCTCCTAGTGTCATTAAACTTT
ATAGTGTTTCGTAACAATGTCTCTTTTCATTCATCAATTGGCCACTGCACAGTTTGGATTGATTGATTGT
TATTATTCGTCATTTAAAAAAAATACATATATAAACACAAATACTTTTTTTTTAAGTGACCGG
>GFIS01070449.1 TSA: Salmo trutta NST_TRBC.2.2 transcribed RNA sequence
ACCCCATAACAATTAACCCTTATACTGGCCAGGACGTTAACATTTTGCTAAAAAAAAAGCCAATTTAGAC
AGGCCCACTGGGCTAAAGATGAATCAGTCCATCTGGCATTTGCCAGAATTGCCCATTTGCCAGTCCGTCC
TTGATCACATGGAAGAATCAACACTCGTGCAGGACATCCTCCTTGGGCCGCTTGTATTCTGTTAAATCTG
TTTCTCTGAGGAGCCCTGGAACTTCCAAATCATAACCATGACGACCAGGCCGTAGAAGGTACTCTTAGCG
ATGAAGATGCTGTAGGCCAGCTTGGCAGTCCGGGTGCTCTTCACATAGTAATCTGTCGTTATCTCTCCCC
CACTTTGACCTTGGAGATCTCCACTGATGGTGTGTTTCACATTTATATATCCATTCCCATTGTAAAAGCT
GACAATGCAGGTGAATCTGTTGTCTGGGTTTTGCCAGTCTTTGGCTGGGACTCTCAGTCTGCTGGTGATA
CTGTATAAACTATCTTTATCCCACAAGGCCTTGTTGTCGGTCCCGGCGCCTTCAGTTCTGTTGACATTGT
TGACTTGCCAGAAGACCGTGACATGGTCGGGGTAGAAGCGGGTGGCTACACACACCAGGGTCTTCTTCTT
CTTGTTGTTTCTATCTTTACACTCCTTAGCCGAGGGTGCTAGGACTTTCACTGTGGGTTCAGTGACTTTG
ATGTTTGGATCTAAAACGGTGAGTTTGGTGCCGGCTCCAAAATATGCCTCCGAGTACGAGCTGGCCTGTC
GGCTCACAGCACAGAAGTACATGCCGCTGTCTCCTGTCTCCAACTTCTTCACTGTGAAAGATCCACTCTC
AACTACAGTCTTCTTGGCTGAGAATTTGTCTTCACTGAAATCCCCTGAATACTCAGGTTTGGAATTGGGA
ATAGTGAAGGCTACCTGCTTCATTCCCTCTCCTGGAAGCTGTCTGTACCAGTACATCTGGATGTGGCCAG
GTCCCTTAGTGTGACTGCAGTTCATCAGAGCATCACTGTCTTTCAGTCCCCAGAGTATGGTGGGTATCTG
AGTGACTTCACTCCCCTCAACAAGACCTGCAGCACAGAGCAGTGAGACAGTAACAGTGAAGAGAAATGTG
AACATGTTTGATCACGTTATATGTGAGAGTGCTGGAAAGAGTCTGGTATCAATGTATATAAAACAAGAGG
AGTGTTACATAGATGAATAGATGACAATCAGTAGACCACGGG
>GDQG01028527.1 TSA: Oncorhynchus kisutch transcribed RNA sequence
GAAGCCTCCTCTAATTCATGTGGAATGGTTTGTCTGTCTCATTCTTTCTGTCTCCTTAACAGTGAGTCAA
CATGATCAGAATTCTTATCTCCATCACCATGGGTTACACAGCCTGGGCTGCAGGTTCTTCTCTCAGTAAC
CAGGTACACCAGAGTCCTGCATCTCTGTACAAGTACCAGGGAGAGTCGGCTAAGATGAAGTGTTCACATA
GTATATCAGGCTATAATCGTATCCTCTGGTACAAGAAATCCAACTCCAGAGAATTTGTGTATTTAGGATA
CCTGAATGTGAAAACTGGATTTCCTGAGGTTGGATTTGATATAGAAGGAGATGCTAATGCAGGTGGTACC
AGCACCTTAACCATCAAACAACTAACTCCAAATAGCAGTGCTGTGTATTACTGTGCTTGGGGGGCGAACT
```

```
CGGAGGCATATTTTGGAGCCGGCACCAAACTCACCGTTTTAGATCCAAACATCAAAGTCACTGAACCCAC
AGTGAAAGTCCTAGCACCCTCCGCTAAGGAGTGTGAAGATAGAAACAAGAAGAAGAAGAAGACGTTGGTG
TGTGTGGCCACCGACTTCTACCCCGACCACGTCACTGTGTTCTGGAAGTTAAATGGAGGCGCCAACATCA
CCGATGGAGTGGGGACCGACAACACTGCCTTGAGGGATGAAAACAGACGCTACAGTATCACCAGCAGACT
GAGAGTCCCAGCCAATGAATGGCACAAACCAGAGAACAGATTCACCTGCAGTGTCAGCTTTTACAATGGG
ACTGACAATATATATGTGAATGACACCATTAGTGGAGATCTCCAAGGTCAAACTGGGGGAGAGATAACGA
CAGATTACTATGTGAAGAGCACCCAGACTGCCAAGCTGGCCTACAGCATCTTCATCGCTAAGAGTACCTT
CTACGGCCTGGTCGTCGTGGCTCTGATTTGGAAGTTTCAGCGCTCCTCAGATAAACAGATGTAATTGATC
CCTGACCGAGACCAGGCCACCTGTGGAAGCTGAGCTCAGTGGACTGGAGGGAGTCATGTGTGGCATAAG
>GFIT01064185.1 TSA: Salvelinus fontinalis transcribed RNA sequence
TGAATGACAGAGAGATCTGAGGTAGTATCTGTTCTCTTTATCTCTGAGTATCTCTGTTCATTTTCCCTAG
AGGGTGAATGACAGAGAGTTCTGAGGTATTTTTGGAATAGGAGAGTAGCGATGTGACACTGGAAGTGTGC
CCTACTTTGGGAATGGAACCAAACTCACTGTTTTAGATCCAAACATCAAAGTCACTGAACCCACAGTGAA
AGTCCTTCCACCCTCCACTAAGGAGTGTGAAGACAGAAACAAGAAGAAGAAGAAGACCCTGGTGTGTGTG
GCCACCGACTTCTACCCCGACCACGTCACCGTGTTCTGGCAGTTGAATGGAGGCGCCAACATCACCGATG
GAGTTGGGACCGACAACACGGCCTTGAGGGGTAAAAACAGACTCTACAGTATCACCAGCAGACTGAGAGT
CCCAGCCAAGAAATGGAACAAGGCCTCTAACAGATTCACCTGCACCGTCCGCTTCTTCAATGGGACCACT
GACATATATGTTGCAGATCACATTAACGGAGAAGAAGGTCAAGGTGGTGATGGAGGGATTACGACAGAGT
ACTATGTGAGGAGCACTAAGACTGCCAAGCTGGCCTACAGCATCTTCATCGCTAAGAGTACCTTCTACGG
CCTGGTCGTCATGGTTATGATTTGGAAGTTCCAGG
```

1.12 Location of TRB01C sequences in North American Atlantic salmon

tBlastN of Atlantic salmon TRB01C deduced amino acid sequence against Atlantic salmon North American St. John River breed genome GCA_021399835.1.

Match #1 chr.23 CM037960.1:

```
Query  1        EPDIpvtppkvkvlppstkECEDRNkkkkkTLVCVATDFYPDHVTVFWQLNGGANITDGV  60
                EPDIPVTPPKVKVLPPSTKECEDRNKKKKKTLVCVATDFYPDHVTVFWQLNGGANITDGV
Sbjct  4758752  EPDIPVTPPKVKVLPPSTKECEDRNKKKKKTLVCVATDFYPDHVTVFWQLNGGANITDGV  4758931

Query  61       GTDNTALRDGNRRYSITSRLRVPAKKWNKASNRFTCTVRFFNGNDDIYVADH  112
                GTDNTALRDGNRRYSITSRLRVPAKKWNKASNRFTCTVRFFNGNDDIYVADH
Sbjct  4758932  GTDNTALRDGNRRYSITSRLRVPAKKWNKASNRFTCTVRFFNGNDDIYVADH  4759087
```

Match #1 chr.23 CM037960.1:

```
Query  1        EPDIpvtppkvkvlppstkECEDRNkkkkkTLVCVATDFYPDHVTVFWQLNGGANITDGV  60
                EPDIPVTPPKVKVLPPSTKECEDRNKKKKKTLVCVATDFYPDHVTVFWQLNGGANITDGV
Sbjct  3862933  EPDIPVTPPKVKVLPPSTKECEDRNKKKKKTLVCVATDFYPDHVTVFWQLNGGANITDGV  3862754

Query  61       GTDNTALRDGNRRYSITSRLRVPAKKWNKASNRFTCTVRFFNGNDDIYVADH  112
                GTDNTALRDGNRRYSITSRLRV AKKWNKASNRFTCTVRFFNGNDDIYVADH
Sbjct  3862753  GTDNTALRDGNRRYSITSRLRVLAKKWNKASNRFTCTVRFFNGNDDIYVADH  3862598
```
