# Supplementary material for: Tetraploid Ancestry Provided Atlantic Salmon With Two Paralogue Functional T Cell Receptor Beta Regions Whereof One Is Completely Novel
Source: Front Immunol. 2022 Jun 17;13:930312. doi: 10.3389/fimmu.2022.930312 (PMC9247247; doi:10.3389/fimmu.2022.930312)

SF2. Phylogeny of deduced Atlantic salmon TRBV amino acid sequences

TRBV subgroups are shown on the right hand side. The optimal tree with the sum of branch length = 8.17563612 is shown. The analysis involved 102 amino acid sequences. There were a total of 112 positions in the final dataset. Pseudogene sequences are not included. Some internal bootstrap values are omitted for clarity. See main text for details on method.

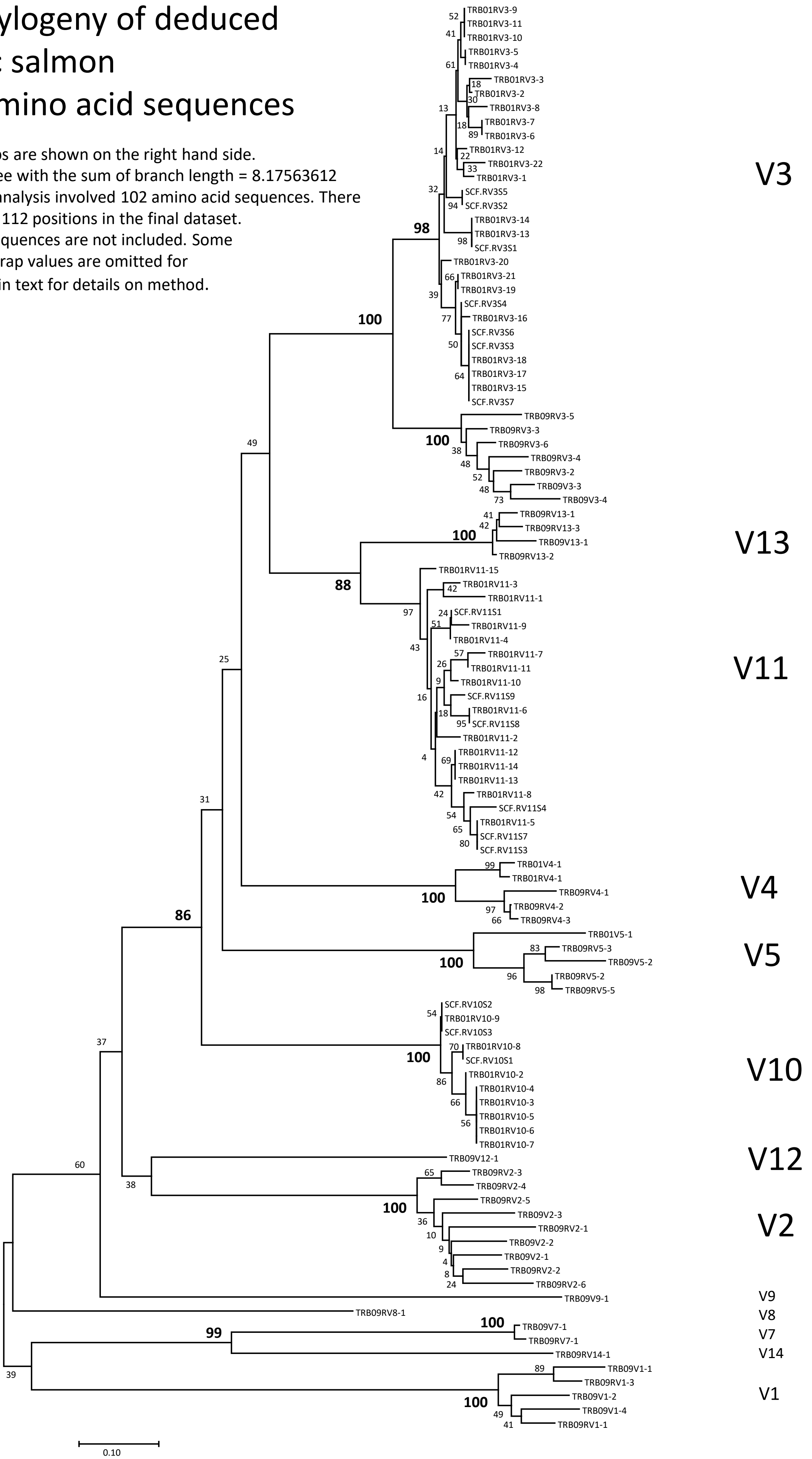

Supplement: Supplementary file 2 [file DataSheet_2.pdf]
